# Ultrafast charge transfer coupled with lattice phonons in two-dimensional covalent organic frameworks

**DOI:** 10.1038/s41467-019-09872-w

**Published:** 2019-04-23

**Authors:** Tae Wu Kim, Sunhong Jun, Yoonhoo Ha, Rajesh K. Yadav, Abhishek Kumar, Chung-Yul Yoo, Inhwan Oh, Hyung-Kyu Lim, Jae Won Shin, Ryong Ryoo, Hyungjun Kim, Jeongho Kim, Jin-Ook Baeg, Hyotcherl Ihee

**Affiliations:** 1 Department of Chemistry, Korea Advanced Institute of Science and Technology (KAIST), Daejeon, 34141 Republic of Korea; 2 KI for the BioCentury, Korea Advanced Institute of Science and Technology (KAIST), Daejeon, 34141 Republic of Korea; 3 Center for Nanomaterials and Chemical Reactions, Institute for Basic Science (IBS), Daejeon, 34141 Republic of Korea; 4 Artificial Photosynthesis Research Group, Korea Research Institute of Chemical Technology (KRICT), Daejeon, 34114 Republic of Korea; 5 Korea Institute of Energy Research (KIER), Daejeon, 34129 Republic of Korea; 6 Department of Chemical Engineering, Kangwon National University, Gangwon-do, 24341 Republic of Korea; 7 Department of Chemistry, Inha University, Incheon, 22212 Republic of Korea; 8 Present Address: Chemical Sciences and Engineering Division, Argonne National Laboratory, Lemont, Illinois 60439 USA; 9 Present Address: Memory Business, Samsung Electronics, Gyeonggi-do, 18448 Republic of Korea

**Keywords:** Metal-organic frameworks, Photocatalysis, Electron transfer

## Abstract

Covalent organic frameworks (COFs) have emerged as a promising light-harvesting module for artificial photosynthesis and photovoltaics. For efficient generation of free charge carriers, the donor–acceptor (D-A) conjugation has been adopted for two-dimensional (2D) COFs recently. In the 2D D-A COFs, photoexcitation would generate a polaron pair, which is a precursor to free charge carriers and has lower binding energy than an exciton. Although the character of the primary excitation species is a key factor in determining optoelectronic properties of a material, excited-state dynamics leading to the creation of a polaron pair have not been investigated yet. Here, we investigate the dynamics of photogenerated charge carriers in 2D D-A COFs by combining femtosecond optical spectroscopy and non-adiabatic molecular dynamics simulation. From this investigation, we elucidate that the polaron pair is formed through ultrafast intra-layer hole transfer coupled with coherent vibrations of the 2D lattice, suggesting a mechanism of phonon-assisted charge transfer.

## Abstract (web summary)

The donor–acceptor (D-A) conjugation has been adopted for two-dimensional (2D) covalent organic frameworks (COFs) for efficient generation of free charge carriers. Here, the authors investigate the dynamics of photogenerated charge carriers in 2D D-A COFs by combining femtosecond optical spectroscopy and non-adiabatic molecular dynamics simulation.

## Introduction

Covalent organic frameworks (COFs) with periodically ordered architecture have attracted much interest as a promising functional material for artificial photosynthesis and photovoltaic devices. In COFs, organic molecules are covalently bonded to each other to form two-dimensional (2D) or three-dimensional porous crystalline structures^1–6^. Specifically, 2D COFs consist of π-conjugated aromatic molecules forming a 2D layer and the 2D layers stack up on top of each other to form a three-dimensional structure. Electronic interaction among the building blocks of a 2D COF and inter-layer π–π interaction among the stacked layers of COFs provide efficient pathways for photogenerated charge carriers. Due to their robust structure and high charge mobility through the framework, the COFs have the potential to serve as a primary light-harvesting module in photovoltaic and optoelectronic devices^2–5,7–10^.

In artificial photosynthetic systems such as organic polymer solar cells, photons are converted into electrical energy via a series of steps^11–17^. First, photons are absorbed by chromophores to generate charge carriers bound by Coulombic interaction (i.e., excitons). Then, the excitons diffuse toward a heterojunction, e.g., the polymer/fullerene interface, where an exciton is separated into free charge carriers (i.e., an electron and a hole). This process of charge separation in artificial light-harvesting systems occurs on ultrafast time scales ranging from tens of femtoseconds (fs) to several picoseconds^12–16^, as commonly observed in natural photosynthesis^18–20^.

For excitons to be separated into free charge carriers, the exciton-binding energy should be overcome. In this regard, for high-performance solar cells, it is desirable to have a light-harvesting system where excitations of low exciton-binding energy are generated. As a means of lowering the exciton-binding energy, researchers have recently developed 2D COFs consisting of two types of repeat units with different electron affinities^7,8,21,22^, borrowing the concept of low-bandgap polymers employing a donor–acceptor (D-A) conjugation scheme^12,14^. In those D-A COFs, the repeat units with low and high electron affinities can serve as an electron donor and an electron acceptor, respectively. As a result of the D-A conjugation, photoexcitation of 2D D-A COFs would lead to the formation of a polaron pair, which is a precursor to free charge carriers and has lower binding energy than an exciton, as in the low-bandgap D-A copolymers. Although the character of the primary excitation species is crucial for determining optoelectronic properties of a material, excited-state dynamics leading to the creation of a polaron pair in the 2D D-A COFs have not been investigated yet. According to previous studies of photoinduced charge carrier dynamics in D-A COFs^21,22^, it was suggested that light absorption induces charge transfer between each donor and acceptor units within 1.8 ps and the charges stay separated for up to 10 μs due to charge delocalization in two separate donor and acceptor arrays aligned perpendicular to stacked 2D layers. This result shows a unique molecular configuration of D-A COFs where the D-A interfaces serve as a well-ordered columnar heterojunction throughout the three-dimensional framework.

In this work, by applying both time-resolved spectroscopic experiment and theoretical simulation, we investigate the earliest stage (on sub-ps time scale) of charge carrier dynamics in 2D D-A COFs consisting of 3,4,9,10-perylenetetracarboxylic acid diimide (PDI) and free-base porphyrin. As the two constituent units have different electron affinities from each other, the 2D D-A COFs contain the D-A conjugation through π-electronic interactions between the building blocks. To elucidate the dynamics of photogenerated charge carriers in the 2D D-A COFs, fs-transient absorption (fs-TA) spectroscopy and non-adiabatic molecular dynamics (NA-MD) simulation are employed in this work. From the fs-TA measurement, we find that excited charge carriers are transferred between PDI and free-base porphyrin on ultrafast time scale with 124 fs time constant, leading to the formation of polaron pairs in the 2D lattice of COFs. From the systematic theoretical simulation based on time-dependent density functional theory (DFT), the ultrafast charge transfer can be specifically assigned to the migration of holes from PDI to free-base porphyrin unit. In addition, we demonstrate that this intra-layer charge transfer is coupled with lattice vibrations manifested as coherent oscillations of TA spectra, which is a compelling evidence for a mechanism of phonon-assisted charge transfer in the periodically ordered structure of COFs.

## Results

### Femtosecond transient absorption spectroscopy

**Fig. 1:**
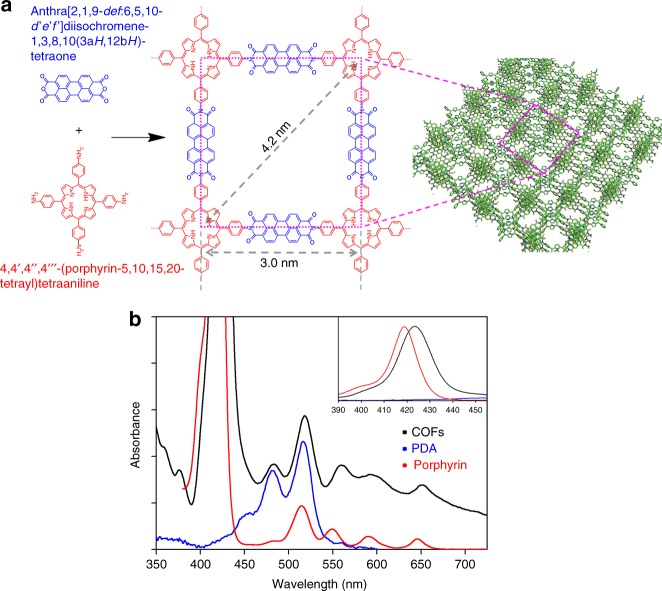
Molecular structure and steady-state absorption spectra. **a** Molecular structure of two-dimensional covalent organic frameworks (COFs). For the clear visualization, the repeat units in 2D sheets are truncated from the stacked structure of COFs (green skeleton). The COFs consist of two units, 3,4,9,10-perylenetetracarboxylic acid diimide (PDI) and free-base porphyrin, which are colored in blue and red, respectively. **b** Electronic absorption spectrum of PDI-porphyrin COFs dispersed in DMF (black), together with the normalized absorption spectra of their precursor molecules, free-base porphyrin (red), and perylene-3,4,9,10-tetracarboxylic dianhydride called as PDA (blue)

Figure 1a shows the molecular structure of synthesized COFs composed of PDI and free-base porphyrin (i.e., PDI-porphyrin COFs). The 2D D-A COFs were synthesized by direct polycondensation of the building blocks and the details for the synthesis and characterization of the COFs are described in Methods, Supplementary Notes 1–3, and Supplementary Figs. 1–5. The porous structure of COFs was characterized by transmission electron microscopy (TEM), nitrogen isotherm, and powder X-ray diffraction (PXRD). The spacing between 2D layers is clearly visualized by a selected area electron diffraction image and the line spacing was determined to be 0.330 nm as shown in Supplementary Fig. 1. The pore size distribution from the nitrogen isotherm measurement shows a peak at 3.9 nm, which is similar to the distance between the farthest perylene moieties (along the diagonal) of the smallest rectangle unit in a 2D layer. High-resolution-TEM (HR-TEM) images show 1.5 nm and 1.0 nm line spacings, which correspond to (200) or (020), and (220) planes, respectively. As discussed in the Supplementary Note 2, HR-TEM data support the staggered structure along the *c*-axis with slipping along *a*-axis and/or *b*-axis. Moreover, the PXRD pattern is also more consistent with the staggered structure as shown in Fig. 1a. The absorption spectrum of PDI-porphyrin COFs dispersed in *N,N*-dimethylformamide (DMF) has absorption features in the entire visible region, including vibronic progression, as shown in Fig. 1b and Supplementary Fig. 6. The intense absorption peak at 424 nm wavelength is mainly attributed to the B state (or Soret band) of free-base porphyrin unit, which has an absorption peak at 419 nm. The broad absorption from 470 to 700 nm with the peaks at 484, 518, 560, 592, and 652 nm can be assigned to the absorption arising from the electronic interaction between the S_0_ → S_1_ transition of PDI (peaked at 481 and 516 nm) and the Q_y_ and Q_x_ transitions of free-base porphyrin (peaked at 513, 548, 590, and 646 nm). The electronic interaction between PDI and free-base porphyrin units is manifested as spectral red shift and broadening of the absorption spectrum of COFs compared with the absorption spectrum of free-base porphyrin, as shown in Fig. 1b and Supplementary Fig. 6.

**Fig. 2:**
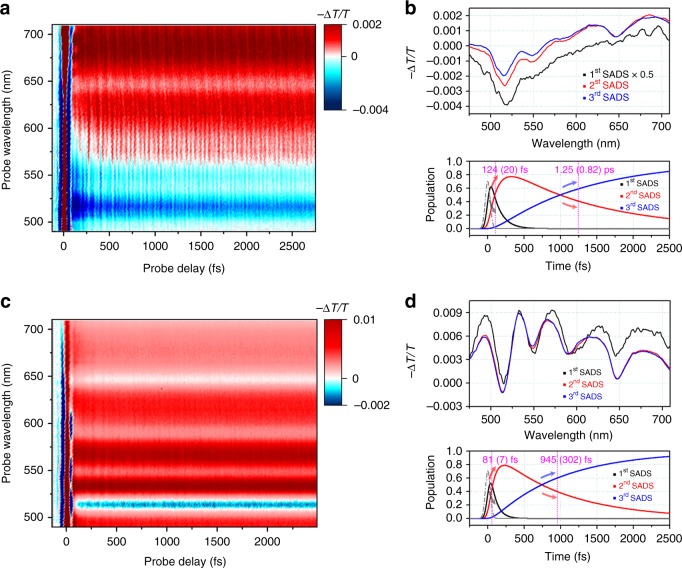
Two-dimensional transient absorption (TA) spectra. **a** Broadband TA spectra of COFs. **b** The upper panel shows the species-associated difference spectra (SADS) of COFs determined from the global kinetic analysis of TA spectra based on the sequential kinetics with the time constant of 124 fs and 1.25 ps. The amplitude of the first SADS was scaled for clear comparison with other SADS. The lower panel shows time-dependent population change of the SADS. The instrumental response function (dashed lines) is plotted together. **c** TA spectra of free-base porphyrin. **d** The upper panel shows the SADS of free-base porphyrin. The lower panel shows time-dependent population change of the SADS

To investigate the dynamics of excited charge carriers of COFs dispersed in DMF, we performed fs-TA measurement. For excitation of COFs, we used the 40 fs pulses of 523 nm center wavelength with the bandwidth of 20 nm full-width-at-half-maximum (FWHM). This photoexcitation condition is predominantly resonant with the electronic transition of PDI units, as supported by the theoretical absorption spectrum calculated by time-dependent DFT calculation that will be discussed later. TA spectra, −∆*T*/*T*(*λ*, *t*), are shown in Fig. 2a. The ground-state bleaching (GSB) and the stimulated emission (SE) contributions are manifested as negative spectral features from 500 to 560 nm, and the excited-state absorption (ESA) contribution is seen as a broad positive feature in the 560–710 nm region. According to previous studies^23–25^, the broad ESA band of free-base porphyrin overlaps with the SE of PDI, making it difficult to resolve the spectral features of porphyrin and PDI in the one-dimensional pump-probe spectrum. To overcome the spectral congestion, we performed global kinetic analysis of the TA spectra based on singular value decomposition (SVD). The details of the global kinetic analysis are described in the Supplementary Note 4 and Supplementary Fig. 7. Based on the result of SVD analysis, we applied a sequential kinetic model as follows:

$${\mathrm{A}}\mathop{\longrightarrow}\limits^{{124\, \mathrm{fs}}}{\mathrm{B}}\mathop{\longrightarrow}\limits^{{1.25\, \mathrm {ps}}}{\mathrm{C}}$$

where A is an initially excited state, and B and C are the excited states involved in the dynamics of excited charge carriers. The spectral features of each state are described by species-associated difference spectra (SADS) obtained from the global kinetic analysis, as shown in Fig. 2b. The transitions among those states were determined to occur with the time constants of 124 (±20) fs and 1.25 (±0.82) ps. As can be seen in Fig. 2b, the first SADS shows strong GSB and SE bands in the spectral region from 490 to 625 nm and these spectral features are in good agreement with the steady-state absorption/emission spectra (Supplementary Fig. 8) and broadband TA spectrum of PDI monomers reported previously^26–28^. Thus, the A state is likely to be associated with PDI units of 2D COFs. The second SADS compared with the first SADS shows that the transition from A to B accompanies quenching of SE and/or spectral broadening of ESA. The SE at 490–625 nm is quenched further with the transition from B to C as can be seen in the spectral and temporal changes from the second SADS to third SADS shown in Fig. 2b and Supplementary Fig. 9. The decay of C state cannot be determined from the global kinetic analysis due to the limited time range of our TA measurement. At the longest time delay of our TA data, the third SADS shows non-zero amplitude, indicating that the ground state is not fully recovered.

To examine the identity of the B state, an additional fs-TA measurement was performed on free-base porphyrin dissolved in DMF. We used the same excitation pulse as the one used for COFs to excite the Q_y_(1,0) transition of free-base porphyrin, of which the absorption spectrum is shown in Supplementary Fig. 10. The global analysis of the TA spectra of free-base porphyrin shows that the first SADS and the second SADS decay with the time constants of 81 fs and 945 fs, respectively, as shown in Fig. 2d. The ultrafast decay of the first SADS and concomitant growth of the second SADS mainly involve the amplitude change in the spectral range of 610–710 nm. When comparing the SADS of free-base porphyrin with its steady-state emission spectrum (Supplementary Fig. 8), we can infer that the change of SADS at 610–710 nm is likely to be associated with the growth of SE contribution arising from the Q_x_(0,0) state of free-base porphyrin, which can be reached by Q_y_-to-Q_x_ internal conversion and vibrational relaxation, as reported previously^23,29,30^. We note that the spectral feature of the valley shape around 645 nm in the second SADS of free-base porphyrin (Fig. 2d) was also observed in the second SADS of COFs (Fig. 2b), indicating that the dip at 645 nm in the second SADS of COFs corresponds to the SE contribution arising from the free-base porphyrin units (Supplementary Fig. 8). Thus, the B state must be associated with porphyrin units of 2D COFs.

Based on the results obtained from the TA measurements on COFs and free-base porphyrin, in the TA spectra of COFs, the quenching of SE at 490–625 nm arising from PDI units concurs with the growth of SE at 645 nm arising from free-base porphyrin units. Thus, the A → B transition of COFs with the time constant of 124 fs can be attributed to the ultrafast transfer of excited charge carriers from PDI to free-base porphyrin. Meanwhile, the excitation at 523 nm can be resonant with both the S_0_ → S_1_ transition of PDI (peaked at 516 nm) and the Q_y_ transition of free-base porphyrin (peaked at 513 nm). Although the relaxation of excited charge carriers generated from direct excitation of free-base porphyrin unit can occur, it cannot account for the large change of TA spectra induced by the A → B transition shown in Fig. 2b, d. In addition, the relaxation of photogenerated carriers localized in the PDI unit can occur due to the existence of defect sites that trap charge carriers. However, according to a previous TA study of perylene bisimide that is analogous to the PDI moiety^31^, the relaxation of excited population in the S_1_ state does not accompany any significant change of TA spectra in the time window up to 2 ps. By considering our fs-TA experiment on free-base porphyrin and the previous spectroscopic study of perylene bisimide^31^, we can confirm that the spectral change accompanying the A → B transition is associated with charge transfer between the building units of COFs rather than relaxation of charge carriers at local chromophores.

### Coherent wavepacket features in spectroscopic data

**Fig. 3:**
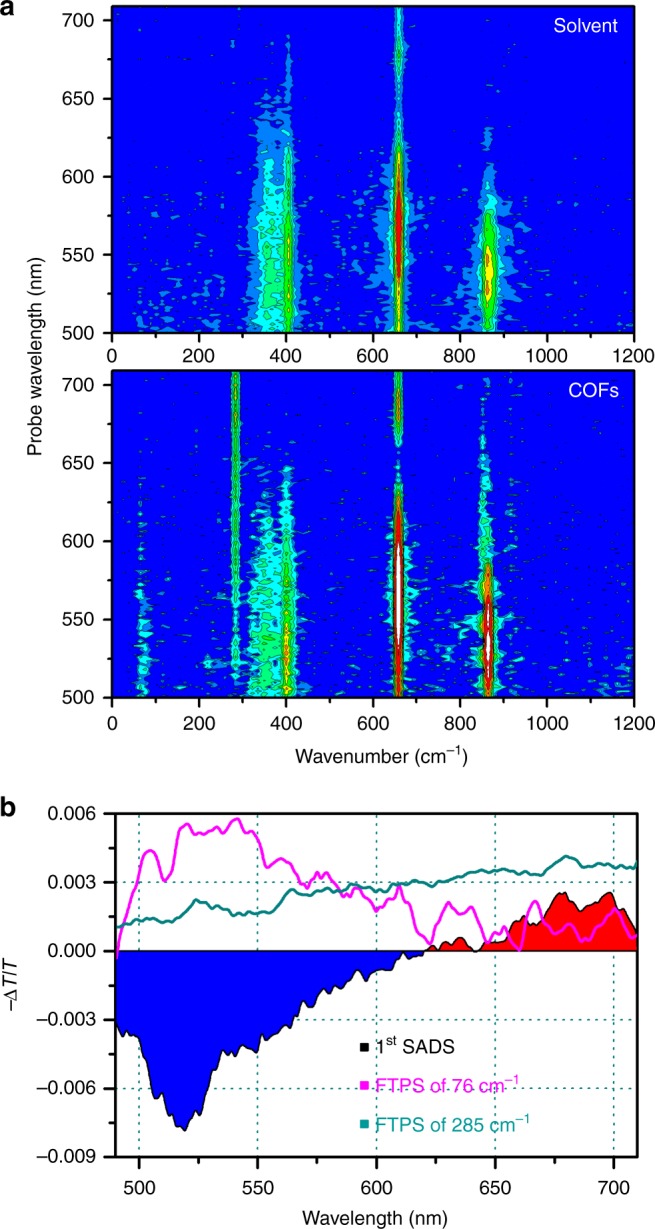
Wavelength-resolved Fourier transform power spectra. **a** Wavelength-resolved Fourier transform power spectra (FTPS) of neat DMF (top) and COFs dispersed in DMF (bottom) with the probe spectral window of 500–710 nm. The Fourier power spectra of COFs have peaks at 76 and 285 cm^−1^, which are absent in the FTPS of pure solvent. **b** Comparison of SADS (black) with the slices of wavelength-resolved FTPS at selected oscillation frequencies. In the SADS, the blue- and the red-shaded area indicate negative and positive amplitude of the TA signal, respectively. The amplitude of the 76 cm^–1^ oscillation is dominant in the spectral range of ground-state bleaching and stimulated emission of the first SADS, whereas the 285 cm^–1^ mode is manifested in the entire spectral range of probe pulse

The TA spectra of COFs shown in Fig. 2a exhibits temporal oscillations superimposed on the monotonous decay arising from the population dynamics of excited states. To extract only the temporal oscillations, we subtracted the excited-state population dynamics, which was fit by the global kinetic analysis, from the TA data. The residuals in the time domain were Fourier transformed to obtain the Fourier transform power spectra (FTPS) shown in Fig. 3a.

Although the excitation pulse is resonant only with the electronic transition of COFs, the solvent still gives non-resonant, impulsive stimulated Raman response and thus also contributes to the FTPS. To distinguish the response of neat solvent from that of COFs dispersed in the solvent, we performed a separate fs-TA measurement on neat DMF. As shown in Fig. 3a and Supplementary Fig. 11, several peaks were observed in the region of 350–1000 cm^−1^ over a broad range of probe wavelengths in the FTPS of both COFs and DMF, and these features are reproduced by the theoretical spectrum reconstructed from four Gaussian functions peaked at 364, 405, 659, and 861 cm^−1^. The frequencies of these peaks observed in the FTPS of DMF are very similar to the vibrational frequencies of 354, 405, 667, and 866 cm^−1^ observed in the Raman spectrum of DMF and thus those peaks can be assigned to the vibrations of C–N out-of-plane bending, CH_3_–N–CH_3_ in-plane bending, O=C–N in-plane bending, and N–CH_3_ symmetrical stretching of DMF, respectively^32^. In addition, the excitation pulse at 523 nm can excite the Q_y_ transition of free-base porphyrin moiety. As shown in Supplementary Fig. 12, the FTPS from the separate fs-TA measurement of free-base porphyrin shows several major peaks, of which vibrational frequencies of 85 and 321 cm^−1^ are distinctly different from the frequencies of 354, 405, 667, and 866 cm^−1^ observed in the neat DMF. Based on the previously reported resonance Raman and theoretical studies^33,34^, the two peaks at 85 and 321 cm^−1^ can be assigned to the torsional motion of side phenyl rings and the in-plane stretching of porphyrin, respectively. From the comparison of FTPS of COFs, neat solvent, and free-base porphyrin in the low-frequency region of <300 cm^−1^, we found that the COFs exhibit two prominent peaks at 76 and 285 cm^−1^, which are absent in the FTPS of neat DMF and free-base porphyrin monomer.

In the 2D plot of temporal oscillations shown in Supplementary Fig. 13, we can see that the coherent oscillations persist over the entire time range of our measurement, which is in stark contrast to the electronic (or vibronic) coherences that generally undergo rapid dephasing^18,35–37^. As the long-lived oscillations of 76 and 285 cm^−1^ frequencies show nearly common phase over the entire range of probe spectrum, without any distinct nodal point (along the axis of probe wavelength) around the probe wavelength where the maximum of steady-state absorption spectrum of COFs is located, we can rule out the possibility that any of the oscillations arise from the ground-state vibrational coherence^38–41^. In Fig. 3b, we compared the slices of wavelength-resolved FTPS at selected oscillation frequencies with the SADS of COFs. The FTPS slice at 76 cm^−1^ has a similar spectral shape as the SE signal of the first SADS, whereas the FTPS slice at 285 cm^−1^ has non-zero amplitude in the entire spectral range of the second SADS. We note that the first SADS in the 490–625 nm region is dominated by the SE of PDI units. Based on this result, we infer that the long-lived oscillations of 76 and 285 cm^−1^ frequencies can be assigned to molecular vibrations of excited-state COFs, specifically the former is related with the PDI units.

### Ab initio non-adiabatic molecular dynamics simulation

NA-MD simulations based on time-dependent Kohn–Sham DFT (KS-DFT)^42,43^ was performed to investigate the ultrafast dynamics of charge carriers observed in the fs-TA measurement. The structure of COFs was modeled by using a periodic unit cell of monolayer (Supplementary Fig. 14) and computational details are described in Methods. To circumvent the problem of underestimating the optical bandgap for the KS energy states (Supplementary Fig. 15), scissor operations were applied to the virtual KS orbital levels (Supplementary Figs. 16 and 17) in order to reproduce the major peaks in the experimental absorption spectrum in the 500–700 nm region. The KS levels were labeled using the highest occupied molecular orbital (HOMO)−*n* and lowest unoccupied mmolecular orbital (LUMO )+*m* notation, which denote the occupied and virtual KS states, respectively, of the simulated COF system, where the larger *n* (or *m*) means the state located farther away from the Fermi level. Due to the quasi fourfold rotational symmetry with respect to the center of free-base porphyrin unit in the 2D COFs, the nearly degenerate KS states were allowed and distinguished using the prime notation; e.g., HOMO−*n*′ or LUMO+*m*′. The low-energy singly excited states, which were chosen as the active space of our surface-hopping (SH) simulations, are labeled as S_a_ to S_x_ (note that the excited states are labeled in terms of LUMO energy in ascending order); the locations of an electron and a hole in each excited state are summarized in Supplementary Table 1.

**Fig. 4:**
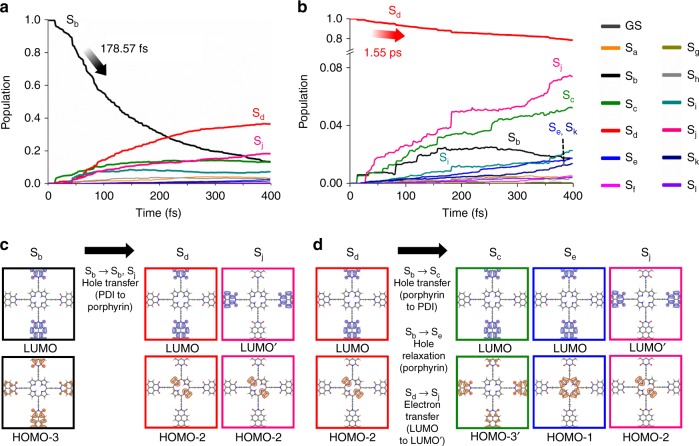
Ab initio non-adiabatic molecular dynamics simulations. **a** Time evolution of electronic populations starting from the initially excited S_b_ state, where the charge carriers are localized in the PDI moieties. **b** Time evolution of electronic populations starting from the hole-transferred state (S_d_), describing the dynamics of charge carriers after PDI-to-porphyrin hole transfer. **c** Evolution of electron-hole distribution starting from the initially excited S_b_ state, visualized with the Kohn–Sham (KS) orbitals of COFs. In each of HOMO and LUMO, the densities of holes (orange) and electrons (blue) are depicted. All possible electron-hole pairs in the active space are listed in Supplementary Table 2. **d** Evolution of electron-hole distribution starting from the hole-transferred state S_d_. In each of HOMO and LUMO, the densities of holes (orange) and electrons (blue) are depicted

From the NA-MD simulation, we found that the photoexcitation at 523 nm employed in the fs-TA measurement induces the following: (1) an electronic transition from HOMO-3 to LUMO, which corresponds to the S_0_-to-S_b_ transition involving the KS orbitals mainly located along the vertical axis of COFs, and (2) an electronic transition from HOMO-3′ to LUMO′, which corresponds to the S_0_-to-S_i_ transition involving the KS orbitals mainly located along the horizontal axis of COFs. For clarity, the directions of vertical and horizontal axes in the structure of COFs are indicated in Fig. 1a. We note that all of the orbitals involved in these transitions are localized at the PDI unit of COFs. The NA-MD simulation results show that the S_b_ state decays with a time constant of 179 fs (Fig. 4a and Supplementary Table 2) and, as a result, the populations of S_d_ and S_j_ states increase predominantly. The S_d_ and S_j_ states have the charge-transfer character with the hole located at porphyrin (HOMO-2) and the electron located at PDI (LUMO in the vertical direction and LUMO′ in the horizontal direction, respectively). Therefore, the transition from S_b_ to S_d_ or S_j_ involves hole transfer from PDI to porphyrin. The S_i_ excited state, which consists of KS orbitals mainly located along the horizontal axis, also relaxes to S_d_ and S_j_ with the time constant of 173 fs, which is a time scale comparable with that of relaxation dynamics of S_b_ state (179 fs time constant) located along the vertical axis, as shown in Supplementary Fig. 18 and Supplementary Table 2.

From the additional set of NA-MD simulations, the relaxation dynamics of the S_d_ state was also investigated. The S_d_ state decays slowly with a time constant of 1.55 ps (Fig. 4b). Concomitantly, the populations of S_b_, S_c_, S_e_, S_i_, S_j_, and S_k_ states increase noticeably. The S_b_, S_c_, and S_i_ states have both the hole (HOMO-3 and HOMO-3′) and the electron (LUMO and LUMO′) located at the PDI moieties, indicating the back transfer of holes from porphyrin (HOMO-2) to PDI (HOMO-3 and HOMO-3′) by the transition from S_d_ to S_b_, S_c_, and S_i_. The S_e_ and S_k_ states have the hole at porphyrin (HOMO-1) and the electron at PDI (LUMO and LUMO′), indicating the relaxation of holes from HOMO-2 to HOMO-1 (at porphyrin) by the transition from S_d_ to S_e_ and S_k_. In the case of the S_j_ state, the electron is located at the horizontal PDI moieties and the hole is at the porphyrin units, indicating the transfer of electrons from vertical PDI (LUMO) to horizontal PDI (LUMO′) by the transition from S_d_ to S_j_.

In addition to the charge carrier dynamics of COFs, vibrational normal modes of the COF monolayer were investigated in order to examine the structural motions associated with the charge carrier dynamics. For readers with interest, all calculated vibrational motions are provided using the animated GIFs in the Supplementary Data 1–13 To investigate the vibrational modes related with the coherent oscillations observed in the fs-TA data, the calculated vibrational modes with the frequencies near 76 and 285 cm^−1^ are summarized in Supplementary Table 3, and the histogram of calculated vibrational modes with frequencies less than 300 cm^−1^ is shown in Supplementary Fig. 19.

## Discussion

The combined results of the fs-TA measurement and the NA-MD simulation provide comprehensive information on the charge carrier dynamics of COFs with the D-A conjugation. In the fs-TA measurement, it was observed that SE is quenched with 124 fs time constant by the A → B transition, which is assigned to charge transfer from PDI to porphyrin. In order to examine the excitation condition of fs-TA experiment, the theoretical linear absorption spectrum of COFs was calculated from the KS-DFT levels in conjunction with the ground-state MD simulation. As shown in Supplementary Fig. 17, the excitation at 523 nm can be resonant with both the HOMO-3 → LUMO transition localized in the PDI moiety (peaked at 514 nm) and the HOMO-1 → LUMO+1′ transition localized in the porphyrin moiety (peaked at 513 nm). As the transition probability of HOMO-3 → LUMO is twice larger than that of HOMO-1 → LUMO+1′, the photoexcitation at 523 nm is likely to dominantly excite the optical transition localized in the PDI moiety. According to the NA-MD simulation on the charge carrier dynamics of COFs, the population decay of initially excited S_b_ state, which corresponds to the transition of HOMO-3 → LUMO, leads to concurrent growth of the populations of S_d_ and S_j_ states with the time constant of ~180 fs, matching the time scale of the ultrafast quenching of SE observed in the fs-TA data. As S_d_ and S_j_ states have holes at the porphyrin units and electrons at the PDI units as shown above, the transition from S_b_ to S_d_ or S_j_ induces hole transfer from PDI to porphyrin, resulting in the formation of a polaron pair in the 2D lattice of D-A COFs. Thus, the NA-MD simulation not only confirms the assignment of A → B transition but also reveals that it is the hole carrier that is transferred from PDI to porphyrin.

The ultrafast charge transfer identified in the present work may originate from the quasi-degeneracy of HOMO-3 and HOMO-2 orbitals, which constitute S_b_ and S_d_/S_j_ states, respectively, and are located along the vertical axis of COFs. The similar energies of the two orbitals are expected to increase the rate of hole transfer in the vertical direction. Such quasi-degeneracy is also observed between HOMO-3′ and HOMO-2 orbitals located along the horizontal axis of COFs, suggesting ultrafast hole transfer (occurring with the transition from S_i_ to S_d_ and S_j_) in the horizontal direction as well.

In addition to the ultrafast dynamics of hole transfer, the kinetic component of 1.25 ps time constant is identified in the TA spectra and assigned to the B → C transition. According to the NA-MD simulation, the relaxation of S_d_ occurs with 1.55 ps time constant, which matches the time scale of the B → C transition. Therefore, this kinetic component can be assigned to the relaxation of the polaron pair states, i.e., (1) porphyrin-to-PDI back transfer of hole carriers (to S_b_/S_c_/S_i_), (2) hole relaxation at porphyrin (to S_e_/S_k_), and (3) electron transfer from vertical to horizontal PDI (to S_j_)^8,22^. We note that a the current NA-MD simulation of COFs was implemented for single-layered COFs, only intra-layer charge transfer, not inter-layer charge transport, was considered. Meanwhile, in a previous TA study on D-A COFs composed of zinc phthalocyanine and naphthalene diimide (i.e., ZnPc-NDI COFs)^22^, the earliest kinetic component with 1.8 ps time constant was observed and assigned to photoinduced electron transfer from ZnPc to NDI unit and subsequent inter-layer charge delocalization along separate donor and acceptor arrays perpendicular to stacked 2D layers. Therefore, considering the similarity of the time scales of the kinetic components obtained from the two TA studies (1.25 ps vs. 1.8 ps), the 1.25 ps component may be attributed to inter-layer charge transport.

Although the pore size of COFs determined from the nitrogen isotherm shown in Supplementary Fig. 5 is close to that of theoretically modeled structure shown in Supplementary Fig. 2 (see Supplementary Note 3), we cannot completely rule out the presence of defects such as mislocation of building units in a 2D layer or less-ordered stacking between the 2D layers. As the defects in the 2D layer may retard or block the excitons initially formed in the PDI moieties from being split into charge carriers (i.e., electrons and holes) through charge transfer between PDI and porphyrin, the spectroscopically measured rates of charge transfer can be regarded as the lower limit for the defect-free material. Considering the prominent change of TA spectra associated with the ultrafast charge transfer between the building units in an individual 2D layer, the observed dynamics is highly unlikely to originate from the defects in a 2D layer. The analyses of TEM images and PXRD pattern show that the synthesized COFs adopts the staggered structure along the *c*-axis with slipping along *a*-axis and/or *b*-axis, as shown in Supplementary Fig. 2b. Such staggered inter-layer structure would result in inefficient charge migration between the stacked 2D layers. Moreover, considering that the TA measurement was conducted for the COF sample dispersed in solution phase, it is possible that the stacked layers in the solid phase can be separated into much thinner stacks or even single layers. All these considerations lead us to conclude that the electrons and holes generated from the photoinduced charge transfer are likely to undergo charge recombination only within individual 2D layers.

**Fig. 5:**
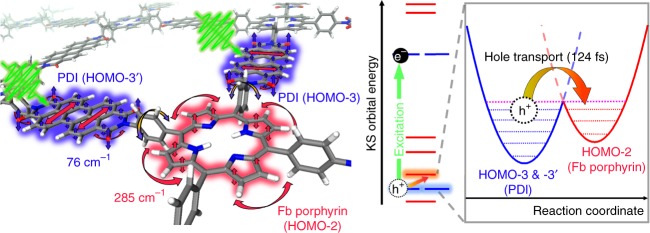
Schematic of charge carrier dynamics in photoinduced COFs. Upon photoexcitation of COFs, ultrafast hole transfer through the splitting of excitons occurs from PDI to free-base porphyrin (Fb porphyrin) unit with the time constant of 124 fs. According to the NA-MD simulation, the quasi-degenerate electronic states of HOMO-3 (or -3′) and HOMO-2 facilitate such ultrafast transfer of charge carriers. The coherent oscillations with the frequencies of 76 cm^−1^ and 285 cm^−1^ superimposed on the decay of TA spectra are associated with coherent lattice phonons of COFs. Especially, the change in the dihedral angle of benzene linker (yellow arrow) can be related with the motions that enhance π-interaction between PDI and porphyrin units, resulting in the ultrafast transfer of charge carriers

The coherent oscillations of 76 cm^−1^ and 285 cm^−1^ frequencies observed in the fs-TA data reflect lattice vibrations coupled with the electronic transition of COFs. The detailed motions of those lattice vibrations can be examined with vibrational normal mode analysis of COFs. As the calculated lattice vibrations in the reticular structure of COFs show complex patterns of motions, we describe those motions in terms of vibrations of constituent units of COFs. The vibrational modes of 74.02 cm^−1^ and 77.12 cm^−1^ have the closest frequencies to the 76 cm^−1^ oscillation identified in the fs-TA measurement and these modes correspond to collective motions including (i) saddling motion of PDI moiety, (ii) in-plane and out-of-plane motions of free-base porphyrin moiety, and (iii) torsional motion of benzene ring that covalently links PDI and free-base porphyrin. The vibrational modes of 282.08 cm^−1^ and 286.26 cm^−1^ frequencies have the closest frequencies to the 285 cm^−1^ oscillation identified in the fs-TA data, and they contain (i) in-plane distortion of PDI moiety without any rotating motion of benzene linker and (ii) in-plane and out-of-plane motions of free-base porphyrin moiety. According to the vibrational normal mode analysis, the distinct difference between the 76 cm^−1^ mode and the 285 cm^−1^ mode is the change of the dihedral angle between the benzene ring and the layer plane of PDI and porphyrin; this angle changes in the 76 cm^−1^ mode, whereas it is almost kept constant in the 285 cm^−1^ mode. The torsional motion of the benzene linker in the 76 cm^−1^ mode can enhance its π-interaction with the PDI and porphyrin by planarization of PDI–benzene–porphyrin units and resultantly facilitates charge transfer between PDI and porphyrin. Therefore, the coherent oscillation of 76 cm^−1^ frequency observed in the fs-TA data is evidence of the intra-layer charge transfer mediated by the torsional motions of intervening π-bridge units. Such an ultrafast intramolecular charge transfer driven by the torsional motion of π-bridge has been also observed in various donor-bridge-acceptor copolymers^44,45^. Based on the population dynamics and the vibrational normal modes of COFs obtained from the quantum mechanical simulation, we conclude that the ultrafast dynamics observed in the fs-TA signal of 2D COFs originate from the ultrafast formation of polaron pair through hole migration between the constituent donor and acceptor units of COFs and such hole transfer is mediated by motions of lattice phonons. We summarize the results of the fs-TA measurement and the NA-MD simulations in Fig. 5, representing the phonon-assisted charge transfer in the 2D D-A COFs.

In this work, by combining fs-TA spectroscopy and NA-MD simulation, we directly observed ultrafast dynamics of phonon-assisted, PDI-to-porphyrin hole transfer in 2D COFs consisting of covalently linked electron donor and acceptor units. The key findings of this work can be utilized for the design of COF-based light-harvesting module applicable to high-performance optoelectronic devices. For example, the 2D D-A COFs can be combined with COFs consisting of only free-base porphyrins. The free-base porphyrin COFs in the form of well-stacked 2D layers exhibit high hole mobility through a periodically ordered macrocycle channel^8,21^. Such hybrid COF architecture will be able to achieve efficient charge separation and charge transport of hole carriers, which are desirable for highly efficient photovoltaic devices.

## Methods

### Synthesis of COFs

For the synthesis of COFs, perylene-3,4,9,10-tetracarboxylic dianhydride and 5,10,15,20-tetrakis(4-aminophenyl)-21*H*,23*H*-porphyrin were prepared separately and then linked together by the polymerization process. In a 500 ml two-neck round-bottle flask, perylene-3,4,9,10-tetracarboxylic dianhydride (4.00 g, 15 mmol) was dissolved in anhydrous pyridine (90 mL). To this solution, a solution of 5,10,15,20-tetrakis(4-aminophenyl)-21*H*,23*H*-porphyrin (3.23 g, 25 mmol) in pyridine (40 mL) was added drop wise. After overnight stirring at 130 °C in argon atmosphere, the reaction mixture was cooled down to room temperature. The reddish-purple precipitate was filtered and washed with copious amount of distilled water, methanol, and chloroform. After drying in the oven at 110 °C, pale purple solid product was obtained. The details for the synthesis and the characterization of COFs are described in the Supplementary Note 1−3.

### fs-TA spectroscopy

TA spectra were measured with fs laser pulses using a visible pump–broadband probe scheme. The output pulses at 800 nm wavelength from a Ti:sapphire amplified laser (Coherent Legend Elite) were split into pump and probe beams. On the pump arm, the 800 nm laser pulses were converted into the pump pulses of 523 nm wavelength and 20 nm bandwidth using a home-built, all-reflective-optic noncollinear optical parametric amplifier. The pump pulses were sent through a pair of Brewster-cut fused-silica prisms to pre-compensate for the dispersion obtained from transmissive optics and compressed to near-transform-limited pulses at the sample position. On the probe arm, the 800 nm laser pulses were sent into a *c*-cut sapphire window of 3 mm thickness and converted into white light continuum spanning from visible to near-infrared wavelengths by self-phase modulation. The visible portion (460–720 nm) of the white light continuum was used as broadband probe pulses without further compensation of the dispersion. The probe pulses were time-delayed with respect to the pump pulses using a motorized translation stage (Newport, M-ILS150HA). By recording “pump-on” and “pump-off” probe spectra, the differential transmission (∆*T*/*T*) spectrum was obtained as a function of time. The spectra of transient signal and the reference were detected by a spectrometer (Andor, SR303i) equipped with a Si CCD (Andor, DU420A). In all the measurements, the polarization of the pump pulses was set to be at a magic angle (54.7°) relative to the probe polarization in order to prevent polarization-dependent signals. For the TA measurement, 2D COFs were suspended in DMF, which causes the exfoliation of the 2D COF layers from the bulk sample through sonication process. This exfoliation procedure improves solubility of COFs that is well suited for the TA spectroscopic measurement^22,46^. The pulse energy of the pump pulses was varied from 63 to 125 nJ to check the excitation energy dependence of TA signal. As the decay profiles of TA data were not dependent on the fluence of pump pulse shown in Supplementary Fig. 20, we did not consider the contribution of exciton–exciton annihilation in the time window of our TA measurement. For the broadband TA measurement, the excitation energy was set to 125 nJ at the sample position in order to achieve a high signal-to-noise ratio. The broadband TA data were measured at various time delays in the time range from −440 to 2728 fs with a time step of 8 fs. To avoid unwanted scatterings of the excitation beam mixed into the TA signal, we cautiously positioned the sample cell in order to reduce any unwanted scattering off the surfaces of the cell. From impulsive stimulated Raman scattering measurement of tetrachloromethane (CCl_4_), the instrumental response function of the TA measurement was determined to be 68 fs from the FWHM of cross-correlation function of the pump and the probe pulses measured at the sample position. The solvent signals were measured with neat DMF in the identical quartz cell as used in the measurement of the COFs sample dissolved in DMF.

### Quantum mechanical simulation

We performed NA-MD simulations as implemented in the PYXAID code^42,43^. This method employs the classical path approximation (CPA)^47,48^—assuming the evolution of nuclear dynamics being independent of the electrons, which allows the practical investigation of the real-time relaxation dynamics of the photoexcited systems with periodic boundary conditions by means of the SH with KS-DFT states. We note that the validity of CPA has been tested against a number of systems, in which electronic excitation barely affects the nuclear dynamics according to previous studies^49–52^. In this regard, the charge-transfer dynamics, accompanied by the structural motion, can be properly explained by the NA-MD simulation based on the PYXAID code.

Using Quantum ESPRESSO program^53^, we first optimized a unit cell of COFs (Supplementary Fig. 14) consisting of the two PDIs and one free-base porphyrin subunit, and then performed a ground-state MD simulation for 500 fs. We employed the Perdew–Burke–Ernzerhof (PBE) exchange-correlation (*xc*) functional^54^ with a plane-wave cutoff energy of 35 Ry and only the gamma point is sampled in the reciprocal space. After applying scissor operations to the virtual states to reproduce the experimental absorption energy (Supplementary Fig. 17), the NA-MD simulations were then performed using the PYXAID code. Active space for the SH dynamics was chosen as the range from HOMO-4 to LUMO+1′ after a thorough convergence check upon the increase of active space size (Supplementary Table 4). All possible single excitation states in the active space are labeled from S_a_ to S_x_ as listed in Supplementary Table 1.

To obtain statistically meaningful population dynamics of excited states, five 400 fs NA-MD trajectories were obtained and averaged. The time constant, *τ*, for the decay of population was determined by fitting the temporal change of excited-state population with an exponential-decay function (1) as follows:

1 $${\mathrm{P}}\left( t \right) = \exp \left( { - \frac{t}{\tau }} \right)$$

We also found that the overcoherence problem of fewest-switches SH^49,55^ should be corrected to obtain a reasonable time constant (Supplementary Fig. 21) and thus we employed decoherence-induced SH^56^ scheme. For the calculations of PXRD patterns and vibrational modes, we used Vienna ab initio simulation package^57,58^ with the choice of same PBE xc-functional and the plane-wave cutoff energy of 450 eV.

## Supplementary information

Supplementary Information

Peer Review File

Description of Additional Supplementary Files

Supplementary Data 1

Supplementary Data 2

Supplementary Data 3

Supplementary Data 4

Supplementary Data 5

Supplementary Data 6

Supplementary Data 7

Supplementary Data 8

Supplementary Data 9

Supplementary Data 10

Supplementary Data 11

Supplementary Data 12

Supplementary Data 13

Supplementary Data 14

## Data Availability

The data that support the findings of this study are available from the corresponding author upon reasonable request.
