# Supplementary material for: Ultrafast charge transfer coupled with lattice phonons in two-dimensional covalent organic frameworks
Source: Nat Commun. 2019 Apr 23;10:1873. doi: 10.1038/s41467-019-09872-w (PMC6478948; doi:10.1038/s41467-019-09872-w)
Supplement: Supplementary file 1 — Supplementary Information [file 41467_2019_9872_MOESM1_ESM.pdf]

Supplementary Information

Ultrafast Charge Transfer Coupled with Lattice Phonons in Two-dimensional Covalent Organic Frameworks

Tae Wu Kim,[†,∆,‖,#] *Sunhong Jun,*[‖,%] *Yoonhoo Ha,*[†] *Rajesh K. Yadav,*[§] *Abhishek Kumar,*[§] *Chung-Yul Yoo,*[‡] *Inhwan Oh,*[†,∆,‖] *Hyung-Kyu Lim,*[⊥] *Jae Won Shin,*[‖] *Ryong Ryoo,*[†,‖] *Hyungjun Kim,* [†,*] *Jeongho Kim,*[¶,*] *Jin-Ook Baeg,*[§,*] *and Hyotcherl Ihee*[†,∆,‖,*]

[†] Department of Chemistry, Korea Advanced Institute of Science and Technology (KAIST), Daejeon 34141, Republic of Korea

[∆]KI for the BioCentury, Korea Advanced Institute of Science and Technology (KAIST), Daejeon 34141, Republic of Korea

[‖] Center for Nanomaterials and Chemical Reactions, Institute for Basic Science (IBS), Daejeon 34141, Republic of Korea

[§]Artificial Photosynthesis Research Group, Korea Research Institute of Chemical Technology (KRICT), Daejeon 34114, Republic of Korea

[‡]Korea Institute of Energy Research (KIER), Daejeon, 34129, Republic of Korea

[⊥]Department of Chemical Engineering, Kangwon National University, Gangwon-do, 24341, Republic of Korea

[¶]Department of Chemistry, Inha University, Incheon 22212, Republic of Korea

Corresponding Authors

*Hyungjun Kim: linus16@kaist.ac.kr

*Jeongho Kim: jkim5@inha.ac.kr

*Jin-Ook Baeg: jobaeg@krict.re.kr

*Hyotchel Ihee: hyotcherl.ihee@kaist.ac.kr

Table of Contents

Supplementary Note 1. Synthesis of covalent organic frameworks 4

Supplementary Note 2. Characterization of covalent organic frameworks 5

Supplementary Note 3. Simulation of powder X-ray diffraction patterns of covalent organic frameworks 8

Supplementary Note 4. Global kinetic analysis of the broadband transient absorption spectra 9

Supplementary Note 5. Wavelength-resolved Fourier transform power spectra extracted from the broadband TA spectra...11

Supplementary Fig. 1. Transmission electron microscopy measurement of COFs. 13

Supplementary Fig. 2. Powder X-ray diffraction patterns of COFs. 15

Supplementary Fig. 3. X-ray photoelectron spectroscopy spectra of COFs...................17

Supplementary Fig. 4. FTIR spectrum and thermogravimetric analysis of COFs. 18

Supplementary Fig. 5. Nitrogen-adsorption isotherms and pore size distribution of COFs. 19

Supplementary Fig. 6. Steady-state absorption spectra of synthesized COFs compared with their precursors. 20

Supplementary Fig. 7. Singular value decomposition analysis of the chirp-corrected transient absorption spectra of COFs...22

Supplementary Fig. 8. Species-associated difference spectra of COFs and free-base porphyrin and steady-state absorption and emission spectra of precursor molecules. 23

Supplementary Fig. 9. Double difference spectra between temporally adjacent species-associated difference spectra of COFs..24

Supplementary Fig. 10. Molecular structure and steady-state absorption spectra of free-base porphyrin...25

Supplementary Fig. 11. Wavelength-resolved Fourier transform power spectra for the oscillatory component of COFs..26

Supplementary Fig. 12. Wavelength-resolved Fourier transform power spectra of free-base porphyrin...27

Supplementary Fig. 13. Two-dimensional plot of residuals obtained by subtracting the electronic population dynamics from the TA data of COFs...29

Supplementary Fig. 14. Periodic molecular structure in a unit cell................................30

Supplementary Fig. 15. Calculated Kohn-Sham DFT energy levels of the COF unit cell. 31

Supplementary Fig. 16. Time evolution of the computed Kohn-Sham DFT energy levels. 32

Supplementary Fig. 17. Linear absorption spectrum of COFs simulated by time-dependent DFT. 33

Supplementary Fig. 18. *Ab initio* Non-adiabatic molecular dynamics simulations for photoexcited charges carrier. 35

Supplementary Fig. 19. Histogram of calculated vibrational modes around 76 cm^{-1} and 285 cm^{-1} frequencies...36

Supplementary Fig. 20. Excitation energy dependence of TA signal.............................37

Supplementary Fig. 21. Time evolution of the surface hopping populations of the electron-hole pair states localized in the PDI subunit. 38

Supplementary Table 1. List of indexes of the singly excited electron-hole pairs in the active space of the KS-DFT orbital ranging from the HOMO-4 to LUMO+1′...............39

Supplementary Table 2. Populations of singly-excited states that were sampled 400 fs after the excitation into S_b, S_d, and S_i states...46

Supplementary Table 3. List of the vibrational normal modes of COFs around 76 cm^{-1} and 285 cm^{-1} frequencies...49

Supplementary Table 4. Time constants for the population decays of excited states after the PDI-centered excitation...50

Supplementary References...51

Supplementary Note 1. Synthesis of covalent organic frameworks

Perylene-3,4,9,10-tetracarboxylic dianhydride, anhydrous pyridine, propionic acid, nitrobenzaldehyde, acetic anhydride, aqueous NH_3, $SnCl_2{\cdot}2H_2O$, and HCl were purchased from Sigma-Aldrich. All the solvents were of HPLC grade and were used without further purification. Ultra-pure water was obtained using a Millipore System (Shinhan Science & Tech).

4,4',4'',4'''-(Porphyrin 5,10,15,20-tetraryl)tetranitrobenzene (1).[1,2] In a 500-ml round bottle flask, 4-nitrobenzaldehyde (11.0 g) and acetic anhydride (12.0 ml) were dissolved in propionic acid (300 ml). The temperature of the solution was then heated to 145 ºC. To this solution, pyrrole (5.0 ml) was added drop wise. The reaction mixture was then refluxed for half an hour and the resulting mixture was removed from the oil bath and cooled to give a precipitate, which was collected by filtration, washed with copious amount of water and methanol, and dried in oven at 110 ºC. The resulting dark purple powder was dissolved in pyridine (80 mL) and refluxed at 115 ºC for 1 h. After cooling, the precipitate was collected by filtration and washed with acetone to give **1** as a purple crystal in 10 % yield.

5,10,15,20-tetrakis(4-aminophenyl)-21*H*,23*H*-porphyrin[4,4',4'',4'''-(Porphyrin 5,10,15,20-tetraryl)tetraaniline]. In a 500-ml two-neck round bottle flask, product **1** (2 g) was dissolved in HCl (200 mL) at 70 ºC, to which was $SnCl_2{\cdot}2H_2O$ (9.0 g) was added slowly. The resulting mixture was stirred at 70 ºC for half an hour and then cooled to 0 ºC. After this procedure, the resultant mixture was neutralized by aqueous NH_3. Following neutralization, the resulting gray crystalline product was collected by filtration and dissolved in acetone. Finally, the solvent was removed by rotary evaporation and dried under vacuum to yield 5,10,15,20-tetrakis(4-aminophenyl)-21*H*,23*H*-porphyrin as a purple crystal. Yield: 85%. ^1H NMR ($CDCl_3$, 300 MHz, ppm): δ -2.7 (s, 2H), 4.0 (s, 8H), 7.0 - 8.1 (m, 16H), 8.8 (s, 8H).

Anal. Calculated for $C_{44}H_{34}N_8$: C, 78.31; H, 5.08; N, 16.61%. Found: C, 77.86; H, 5.27; N, 16.29%.

Synthesis of PDI-porphyrin COFs. In a 500-ml two-neck round bottle flask, perylene-3,4,9,10-tetracarboxylic dianhydride (4.00 g, 15 mmol) was dissolved in anhydrous pyridine (90 mL). To this solution, a solution of 5,10,15,20-tetrakis(4-aminophenyl)-21*H*,23*H*-porphyrin (3.23 g, 25 mmol) in pyridine (40 mL) was added drop wise. After overnight stirring at 130 ºC in argon atmosphere, the reaction mixture was cooled down to room temperature. The reddish-purple precipitate was filtered and washed with copious amount of distilled water, methanol and chloroform. After drying in the oven at 110 ºC, pale purple solid product was obtained (2.21 g, 81 % based on PDI).

Supplementary Note 2. Characterization of covalent organic frameworks

Transmission electron microscopy. To examine the topography of the synthesized COFs, we performed the measurement using transmission electron microscopy (TEM). TEM image was collected by TECNAI G2 F30 microscope (FEI company) operated at 300 kV. Selected area electron diffraction (SAED) pattern, measured with the same instrument, was radially averaged by using the home-built Matlab code in order to assess the *d*-spacing value between the diffraction peaks. An aberration-corrected environmental TEM (Titan ETEM G2, FEI) operated at 300 kV was used for the high-resolution TEM (HR-TEM) characterization. As shown in Supplementary Fig. 1a, the TEM image of COFs powder shows rod-like morphology and significant Bragg peaks in the selected area electron diffraction (SAED) pattern, which clearly shows the interlayer distance between 2D layers. HR-TEM images shown in Supplementary Fig. 1d – 1i clearly show the regular pattern in the 2D layers. In particular, from the TEM image shown in Supplementary Fig. 1e, it can be learned that the spacing between periodic lines is about 1.0 nm and its FT image shown in Supplementary Fig.

1f has Bragg spots, which correspond to (220) planes. The 1.0 nm spacing corresponds to half the distance between the farthest perylene moieties (along the diagonal) of the smallest rectangle formed by 2D layers stacked in the staggered conformation (that is, the diagonal length of the rectangle is 1.5 nm times the square root of 2). In addition, we obtained the Fourier-transformed (FT) image (Supplementary Fig. 1i) with Bragg spots for the lines with the spacing of 1.5 nm, which corresponds to (200), or (020), planes, although its HR-TEM image in Supplementary Fig. 1h is too noisy to observe the 1.5-nm spacing in real space. The 1.5 nm spacing corresponds to half the distance between the nearest perylene moieties that are periodically aligned along *a*-axis or *b*-axis in the 2D layers. Thus, these TEM images strongly support the staggered structure along the *c*-axis with slipping along *a*-axis and/or *b*-axis.

Powder X-ray diffraction. Powder X-ray diffraction (PXRD) measurements were performed by X-ray diffractometer (D/MAX-2500, Rigaku Denki). The PXRD patterns were recorded in the range of $2\theta = 3 – 30^{o}$ with Cu K_{α} radiation (λ = 1.5406 Å) at 40 kV and 200 mA. The crystalline nature of COFs was confirmed by the observed PXRD pattern together with the theoretical X-ray diffraction profile reconstructed from the Pawley refinement as shown in Supplementary Fig. 2a – 2b. The PXRD peak at $2\theta \sim 24.3^{o}$ indicates that the 2D layers are stacked along the (001) plane with the inter-layer distance of ~3.6 Å while exhibiting a typical van der Waals contact of carbon-based layered materials[3-6]. Simulated PXRD patterns using stacked 2D layers with different periodic orderings show significant Bragg peaks representing the crystallinity of COFs. The relative amplitude of X-ray intensity in the simulated PXRD patterns is different from that obtained from the experiment, which implies preferred orientation of powder sample with anisotropic packing of crystallites (details in the Supplementary Note 3).

X-ray photoelectron spectroscopy. We analyzed the atomic species and chemical bonds constituting the COFs using X-ray photoelectron spectroscopy (XPS). XPS spectra were recorded on Axis Nova photoelectron spectrometer (KRATOS). As can be seen in the XPS spectrum in Supplementary Fig. 3, the C 1s peaks at 281.3 eV, 282.4 eV, and 284.9 eV can be assigned to C−C, C−N, and C=O bonds, respectively. The N 1s peaks around 394 eV and 397 eV correspond to pyridinic and pyrrolic nitrogen atoms in the COFs, respectively. The O 1s peak at 528.6 eV indicates the presence of C=O bond[7].

Fourier transform infrared spectroscopy. Fourier transform infrared spectroscopy (FTIR) spectra were obtained with an ALPHA-T FT-IR spectrometer (Bruker). In the infrared spectrum in Supplementary Fig. 4a, the peaks at 1770 cm^{-1} and 1700 cm^{-1} are assigned to the symmetric and asymmetric vibrations of the C=O bond, and a peak around 1350 cm^{-1} corresponds to the stretching vibration of the C–N–C linkage. Besides, any peak around ~3400 cm^{-1} corresponding to N–H stretching in the NH_2 group of 5,10,15,20-tetrakis(4-aminophenyl)porphyrin was not observed, further supporting the formation of COFs with the desired structure.

Thermogravimetric analysis. The thermogravimetric analysis was performed on Q500 analyzer (TA Instruments) in the temperature range from 25 °C to 900 °C at a heating rate of 5 °C min^{-1} under Ar flow. As shown in Supplementary Fig. 4b, the COFs showed high thermal stability without significant decomposition up to 450 °C, as expected for COFs[8,9].

Porosity measurements. The porosity measurements were performed using the ASAP 2420 system (Micromeritics). The Brunauer-Emmett-Teller (BET) surface area of COFs was found to be 309.4 m^2/g. The nitrogen sorption isotherm measured at 77 K exhibits a type IV isotherm indicative of mesopores. The high adsorption at a high relative pressure (P/P_0 ~1) further corresponds to the presence of a mesoporous sample (Supplementary Fig. 5). Moreover, the curve of pore size distribution reveals a pore diameter of ~3.86 nm indicative

of uniformly mesoporous COFs as shown in the inset of Supplementary Fig. 5. Overall, the results from the porosity measurements are in good agreement with those from the previously reported mesoporous COFs in literature[10,11].

Elemental analysis. The elemental analysis for the samples was performed using the FLASH EA-2000 Organic Elemental Analyzer (Thermo Scientific). The experimental data agree well with the expected analysis values; Anal. Calculated for $(C_{92}H_{42}N_8O_8)_n$: C 79.64; H 3.03; N 8.08 %. Found 78.65; H 3.08; N 8.24 %.

Supplementary Note 3. Simulation of powder X-ray diffraction patterns of covalent organic frameworks

To gain the structural information from the experimental PXRD data, the simulation of powder X-ray diffraction patterns of COFs was performed. This simulation was based on the structural modelling of stacked 2D sheets. To do so, the periodic single-layer 2D sheet was optimized by using the Vienna ab initio simulation package (VASP)[12,13] with the density functional theory (DFT). The project-augmented-wave (PAW) method[14] was applied to describe the wave functions in the core regions in order to have comparable accuracy to all-electron calculations. The detailed procedure of structure optimization is described in the following section. Based on this single-layer structure, the lattice parameters of eclipsed structure (space group *P1*, a = 29.84 ± 0.01 Å, b = 29.84 ± 0.01 Å, c = 3.65 ± 0.01 Å, $\alpha = \beta = \gamma = 90^o$) and staggered structure (space group *P1*, a = 29.84 ± 0.01 Å, b = 29.84 ± 0.01 Å, c = 7.30 ± 0.01 Å, $\alpha = \beta = \gamma = 90^o$) were determined by Pawley refinement of the PXRD pattern. From the optimized single-layer structure and refined lattice parameters, the plausible stacked nanostructures were constructed and then the theoretical X-ray diffraction patterns were calculated from those structures as shown in Supplementary Fig. 2c. The Pawley refinement and the simulation of PXRD patterns were carried out using Rietan-FP and VESTA software

package[15]. Regardless of the stacked structural shape of COFs, the predicted PXRD patterns clearly show the intense (200) peak at 2θ = 5.58°. In terms of relative amplitudes of diffraction peaks, the discrepancy between the experimental and the simulated patterns may imply the preferred orientation of powder sample with anisotropic packing of crystallites, which have been reported in the literature[16,17]. Since the TEM measurement supports the staggered structure, we examined which structure between the eclipsed and staggered structures works better for the Pawley refinement as shown in Supplementary Fig. 2a – 2b. The result shows that the PXRD pattern is indeed more consistent with the mixed staggered structures with the A-stacking, B-stacking and AB-stacking. Thus, considering the analyses of TEM images and PXRD pattern, we can conclude that the synthesized COFs have a staggered conformation. As discussed in the main text, the absence of signature for the inter-layer charge migration in the femtosecond TA spectra is consistent with the staggered structure.

Supplementary Note 4. Global kinetic analysis of the broadband transient absorption spectra

We performed the global kinetic analysis of broadband TA spectra. This analysis consists of two steps: (i) A kinetic model consisting of a number of transient electronic states and the rate constants for transitions among the transient states are determined by singular value decomposition (SVD) of the TA spectra. (ii) Species-associated difference spectra (SADS), which are spectral characteristics of the transient electronic states, are extracted by principal component analysis (PCA) of the TA spectra.

Prior to the global analysis, the wavelength-dependent velocity mismatch of probe pulse was corrected using the coherent spike signal from pure solvent (DMF). We applied singular value decomposition (SVD) analysis[18] to the chirp-corrected TA spectra shown in

Supplementary Fig. 7a. By the SVD analysis, the data matrix of TA spectra, $\boldsymbol{A}$, can be decomposed into left- and right-singular vectors using the relationship of $\mathbf{A} = \mathbf{USV}^{\mathrm{T}}$, where $\boldsymbol{U}$ consists of the left-singular vectors (lSVs) corresponding to time-independent spectra as a function of wavelength, $\boldsymbol{V}$ consists of the right-singular vectors (rSVs) corresponding to time-dependent amplitude change of $\boldsymbol{U}$, and $\boldsymbol{S}$ contains the weights of the singular vectors. The lSVs contains the information on the spectra of distinct transient electronic states while the rSVs represent the population dynamics of those transient states. Thus, the SVD analysis allows us to obtain the kinetic components of excited electronic dynamics regardless of the kinetic model. Supplementary Fig. 7b shows the first three lSVs, while Supplementary Fig. 7c shows the first three rSVs scaled with singular values obtained from the SVD analysis. From the global fitting of the three rSVs by a sum of multiple exponentials sharing common relaxation times, the time constants of 124 (± 20) fs, and 1.25 (± 0.82) ps were determined. Upon photoexcitation, the 1st SADS is observed in the time range comparable with the instrument response function (IRF) of 68 fs. In addition, the TA spectra on picosecond time scale still have apparent signal in the entire wavelength window, indicating that the excited carrier dynamics of COFs might be still in progress toward the ground state. Therefore, we can make a sequential kinetic model based on three distinct SADS and two time constants (124 fs and 1.25 ps).

$$\mathbf{A} \xrightarrow{\mathbf{124}\ \boldsymbol{fs}} \mathbf{B} \xrightarrow{\mathbf{1.25}\ \boldsymbol{ps}} \mathbf{C}$$

Based on the above sequential kinetic model and the time constants estimated from the SVD analysis, we implemented principal component analysis (PCA) whereby the experimental TA spectra were decomposed into three SADS corresponding to the three states as follows:

$$TA_{Theory}(\lambda_i, t_j) = \sum_{k=1}^{3}\left[C_k^*(t_j)\right] SADS_{C_k}(\lambda_i), \quad (1)$$

$$C_k^*(t) = C_k(t) \otimes \mathrm{IRF}(t) = \int_{-\infty}^{\infty} C_k(T-t)\ \mathrm{IRF}(T)\ dT, \quad (2)$$

where $TA_{Theory}(\lambda_i, t_j)$ is the theoretical TA spectra at given wavelength (*λ*) and time-delay (*t*) values, $SADS_{C_k}(\lambda_i)$ is the species-associated difference spectra corresponding to the *k*-th intermediate state at a given *λ* value. $C_k(t)$ is the instantaneous population of the *k*-th intermediate at a given *t* value and then is convoluted with the IRF measured from the pure solvent, which is the convoluted kinetic profiles termed as $C_k^*(t)$. After that, we minimized the discrepancy between the theoretical and experimental TA spectra using Nelder-Mead simplex algorithm[19]. The whole analysis was performed by the home-built Matlab code.

Supplementary Note 5. Wavelength-resolved Fourier transform power spectra (FTPS) extracted from the broadband TA spectra

The residuals were obtained by subtracting the electronic population dynamics from the TA signals of PDI-porphyrin COFs, that is, the wavelength-resolved temporal oscillations (arising from vibrational coherences) as shown in Supplementary Fig. 13. The wavelength-resolved temporal oscillations in the time domain were directly Fourier transformed to give the wavelength-resolved Fourier transform power spectra (FTPS), which is the frequency-domain spectra shown in Fig. 3a. The FT window ranging from 104 fs to 2728 fs and the steps of $\boldsymbol{N}$ = 329 resulted in the frequency-domain spectra with a frequency range of 2077 cm^{-1} and a frequency resolution of ~12 cm^{-1}. A series of FT analyses were also performed for the TA data of pure solvent (DMF) and free-base porphyrin dissolved in DMF and, as a result, their wavelength-resolved FTPS were obtained. To find the center position of the FTPS with an accuracy better than the FT resolution of the raw data, we performed the cubic-spline interpolation of the frequency-domain spectra, in which a Gaussian function was used to fit each frequency mode. From the interpolated FTPS of DMF, we determined the frequencies of

ground-state vibrational coherences, 364 cm^{-1}, 405 cm^{-1}, 667 cm^{-1}, and 866 cm^{-1}, which well match Raman frequencies of DMF[20], as shown in Fig. 3 and Supplementary Fig. 11.

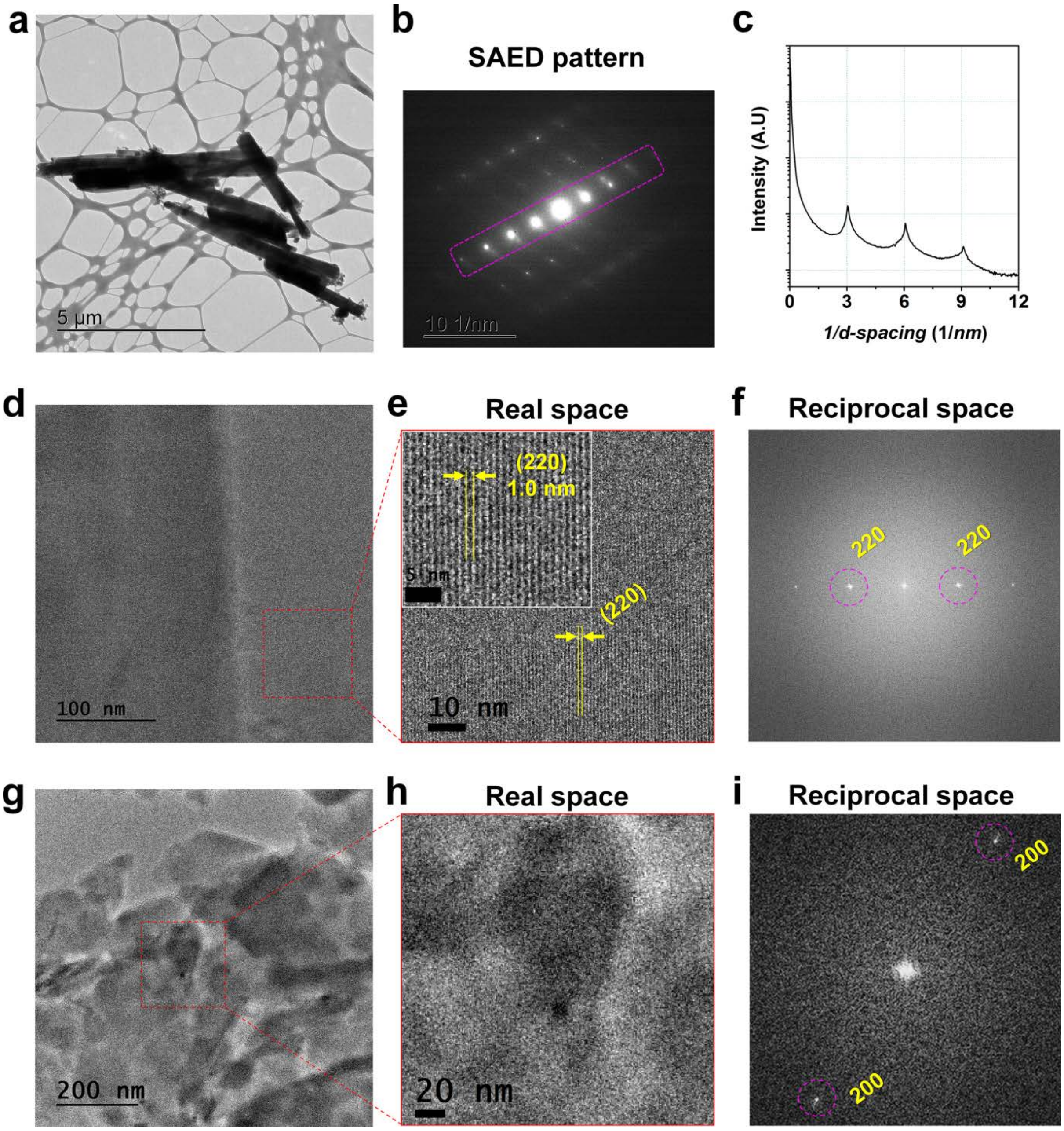

Supplementary Fig. 1. Transmission electron microscopy measurement of COFs. (a) TEM image of COFs in powder form. (b) A selected area electron diffraction (SAED) image. (c) The profile plot of the area indicated by the magenta rectangle in (b). The lattice spacing (*d*-spacing) of 0.330 nm (= 1/3.029 nm) is close to the distance between 2D layers obtained by powder X-ray diffraction (PXRD) patterns of COFs. (d) A high-resolution TEM (HR-TEM) image of COFs for (220) planes. (e) A magnified view of (d). Periodic lines

corresponding to the (220) planes are visible. The upper left inset shows a further magnified view. The line spacing is about 1.0 nm. (f) Fourier-transform (FT) of the HR-TEM image in (e). The Bragg peaks for (220) planes with 1.06 nm spacing are seen. (g) A HR-TEM image of COFs for (200), or (020), planes. (h) A magnified view of (g). (i) FT of the HR-TEM image in (h). The Bragg peaks for (200), or (020), planes with 1.5 nm spacing are seen.

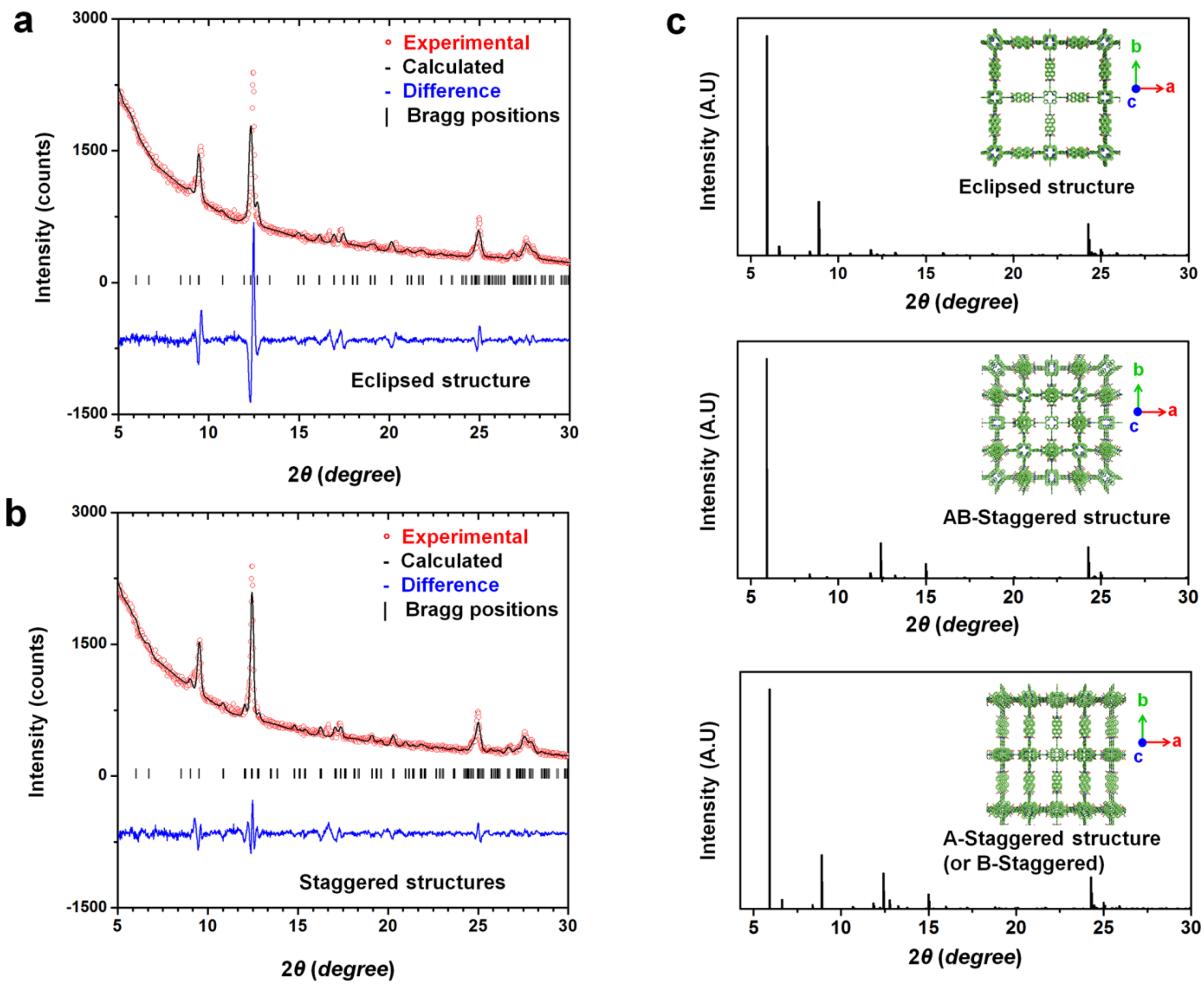

Supplementary Fig. 2. Powder X-ray diffraction (PXRD) patterns of COFs. (a, b) Experimental PXRD pattern of COFs. To confirm the assignment of the observed reflections, we performed the Pawley refinement of the experimental PXRD pattern (red circles) based on the (a) eclipsed and (b) mixed staggered structure, as shown in (c). The positions of Bragg reflections are marked by ticks for each of the eclipsed and staggered structures. The refined profile (black line) with the mixed staggered structure shows better agreement with the experimental PXRD pattern, leaving smaller discrepancy (blue line) between the experimental and theoretically refined patterns. This observation is consistent with the HR-TEM images shown in Supplementary Fig. 1. (c) Simulated PXRD patterns using the three different periodic orderings, i.e. eclipsed, staggered (AB stacking), and staggered (A or B stacking) conformations, of COFs. In each panel, the molecular frameworks of stacked 2D

sheets, constructed from the quantum mechanical optimized structure, are visualized along the direction of *c*-axis perpendicular to the paper plane. The relative intensities of Bragg peaks in the simulated PXRD patterns are different from those in the experimental PXRD pattern, which implies the preferred orientation of powder sample with anisotropic packing of crystallites.

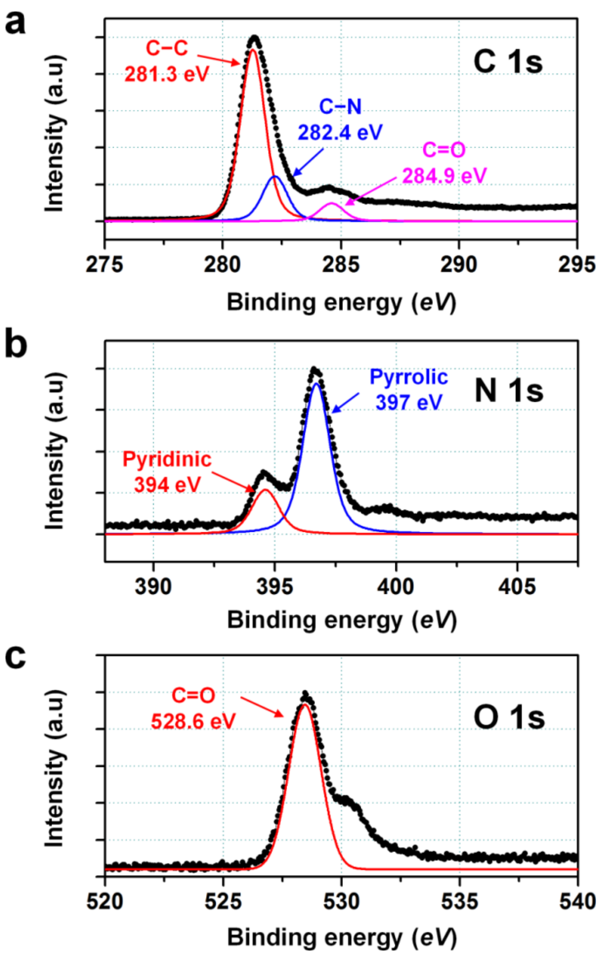

Supplementary Fig. 3. X-ray photoelectron spectroscopy (XPS) spectra of COFs. (a) XPS C 1s spectrum fitted with multiple Gaussian functions (color-coded), which are indexed to C−C, C−N, and C=O bonds, respectively. (b) XPS N 1s spectrum fitted with multiple Gaussian functions (color-coded), which are indexed to pyridinic and pyrrolic nitrogen, respectively. (c) XPS O 1s spectrum fitted with a Gaussian function (red line), which indicates the presence of C=O.

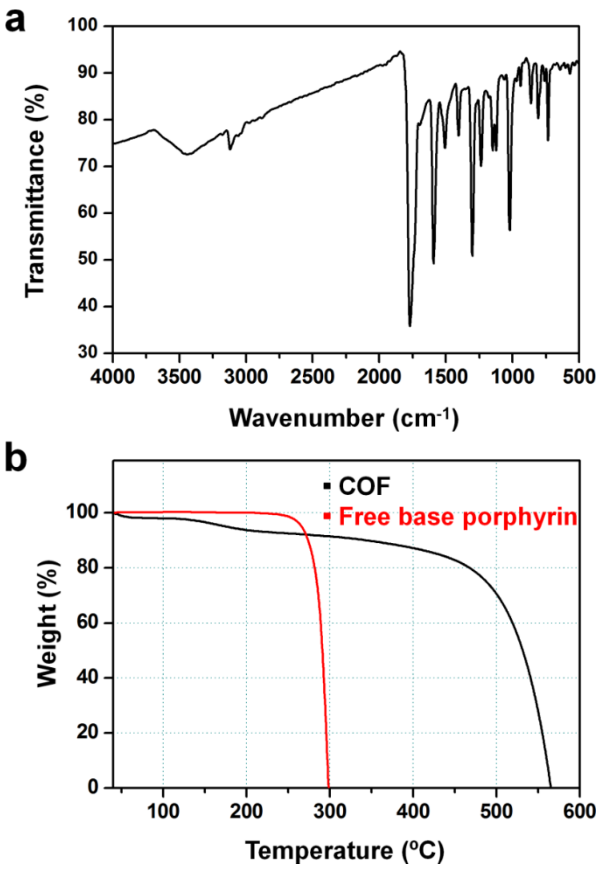

Supplementary Fig. 4. FTIR spectrum and thermogravimetric analysis of COFs. (a) FTIR spectrum of COFs. Any intense peak around ~3400 cm^{-1} related with the N–H stretching mode of NH_2 group of 5,10,15,20-tetrakis(4-aminophenyl)porphyrin is absent in COFs, which supports the formation of the desired structure. (b) Thermogravimetric analysis (TGA) of COFs (black) and free-base porphyrin (red). At 300 ºC, the weight loss of COFs is approximately 9 % while free-base porphyrin is completely destroyed at the same temperature. This shows the high thermal stability of COFs up to 450 ºC.

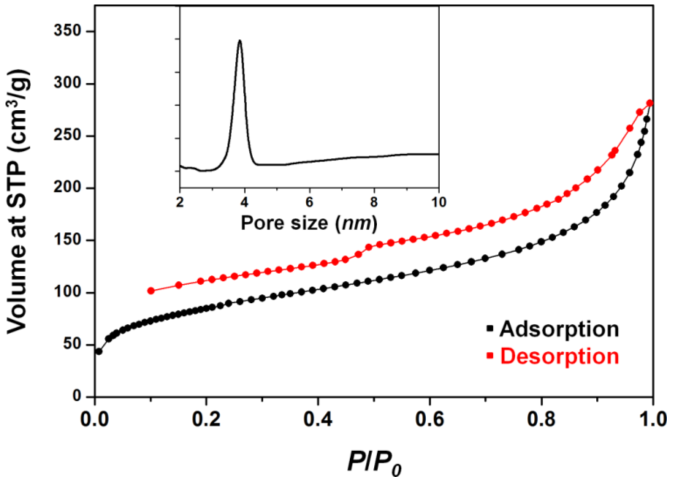

Supplementary Fig. 5. Nitrogen-adsorption isotherms and pore size distribution of COFs. Nitrogen adsorption-desorption isotherm of COFs was measured at 77 K. Inset shows pore size distribution of COFs.

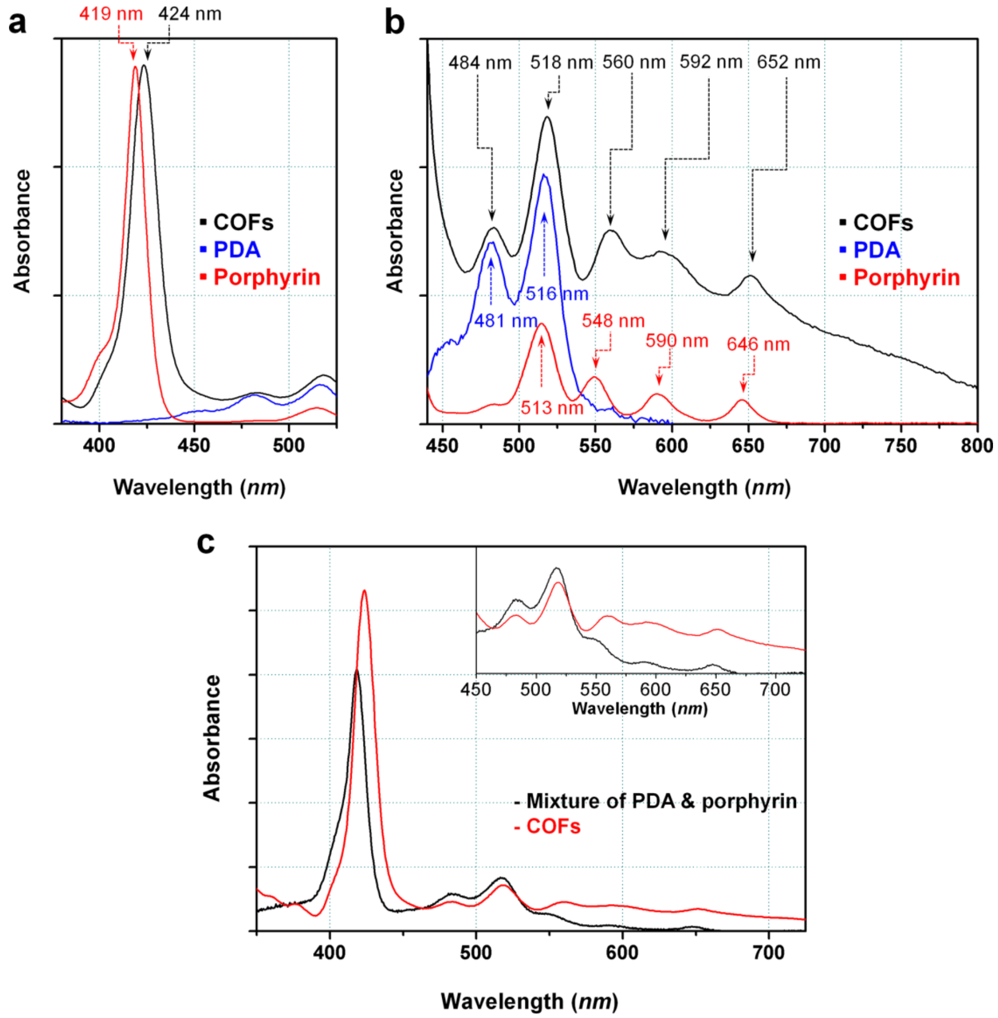

Supplementary Fig. 6. Steady-state absorption spectra of synthesized COFs compared with their precursors. Optical absorption spectrum of COFs dispersed in DMF (black), together with the normalized absorption spectra of their precursor molecules, free-base porphyrin (red) and perylene-3,4,9,10-tetracarboxylic dianhydride called as PDA (blue). (a, b) Absorption spectra of COFs and their precursors in the spectral range (a) from 380 nm to 525 nm, and (b) from 440 nm to 800 nm. The conjugation of PDA and free-base porphyrin moieties through the covalent bonds induces the spectral red shift of absorption maxima as well as broadening of the absorption band up to the near-infrared region. (c) Comparison of absorption spectrum of COFs (red) with that of a physical mixture of their precursor molecules (black). While the physical mixture has low absorption at 575 – 660 nm, the

synthesized COFs have strong absorption in a broad spectral range from 575 nm beyond 700 nm.

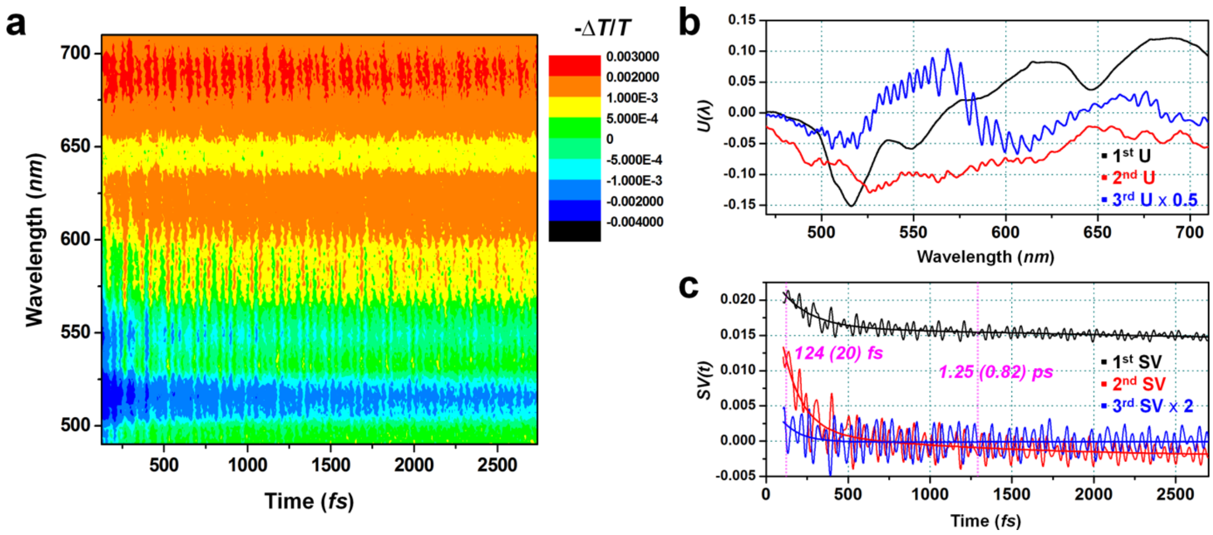

Supplementary Fig. 7. Singular value decomposition (SVD) analysis of the chirp-corrected transient absorption spectra of COFs. (a) The time-zero corrected transient absorption spectra. (b) The first three left-singular vectors (lSV or ***U***). (b) The most significant right-singular vectors (rSVs or ***V***) multiplied by their corresponding singular values (S). The rSVs were globally fitted by two exponential functions (solid lines), yielding the time constants of 124 fs and 1.26 ps.

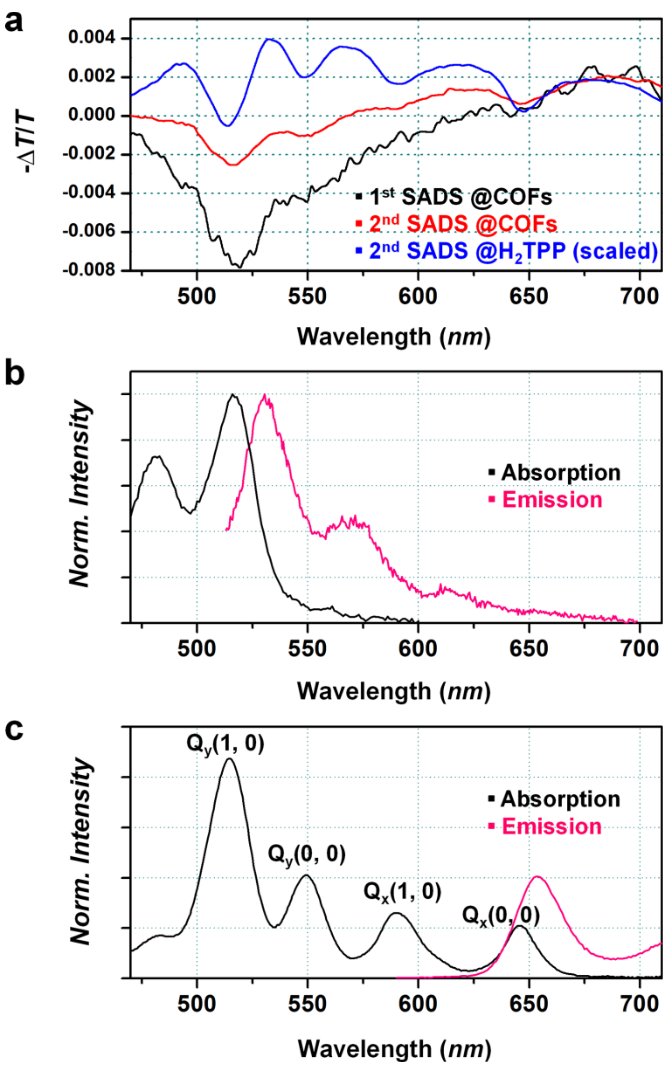

Supplementary Fig. 8. Species-associated difference spectra (SADS) of COFs and free-base porphyrin and steady-state absorption and emission spectra of precursor molecules. (a) 1st SADS and 2nd SADS of COFs are compared with 2nd SADS of free-base porphyrin (H_2TPP). (b) Linear absorption spectrum of PDI monomer (black line), together with the fluorescence emission spectrum (magenta line) measured with the diluted solution and the excitation at 483 nm. (c) Absorption (black line) and emission (magenta line) spectra of PDI monomer. The fluorescence emission spectrum was measured with the excitation at 400 nm. The front and back numbers in parenthesis are the vibrational quantum numbers of the dominant vibrational mode in the upper and lower electronic states, respectively.

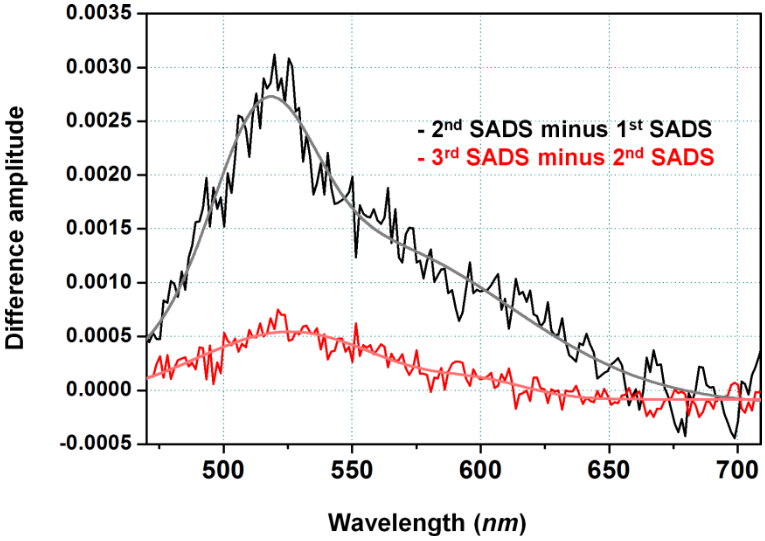

Supplementary Fig. 9. Double difference spectra between temporally adjacent species-associated difference spectra (SADS) of COFs. The difference of 2nd SADS and 1st SADS are scaled with the factor of 0.5 for clear comparison with that of 3rd SADS and 2nd SADS.

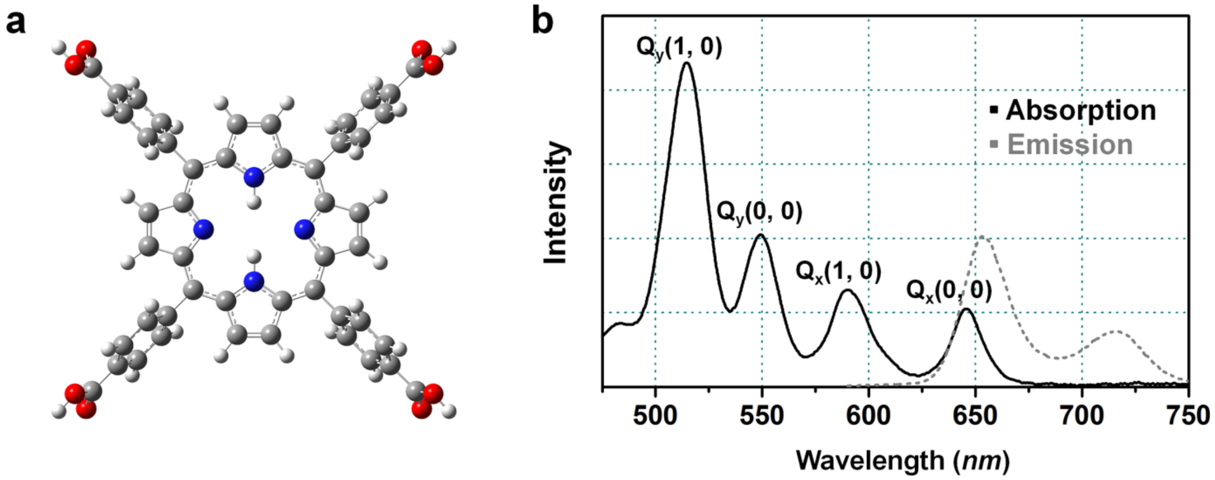

Supplementary Fig. 10. Molecular structure and steady-state absorption spectra of free-base porphyrin. (a) Molecular structure of free-base porphyrin. (b) Electronic absorption spectrum of free-base porphyrin (solid line), together with the fluorescence emission spectrum (dotted line) measured with the excitation at 400 nm. The front and back numbers in parenthesis are the vibrational quantum numbers of the dominant vibrational mode in the upper and lower electronic states, respectively.

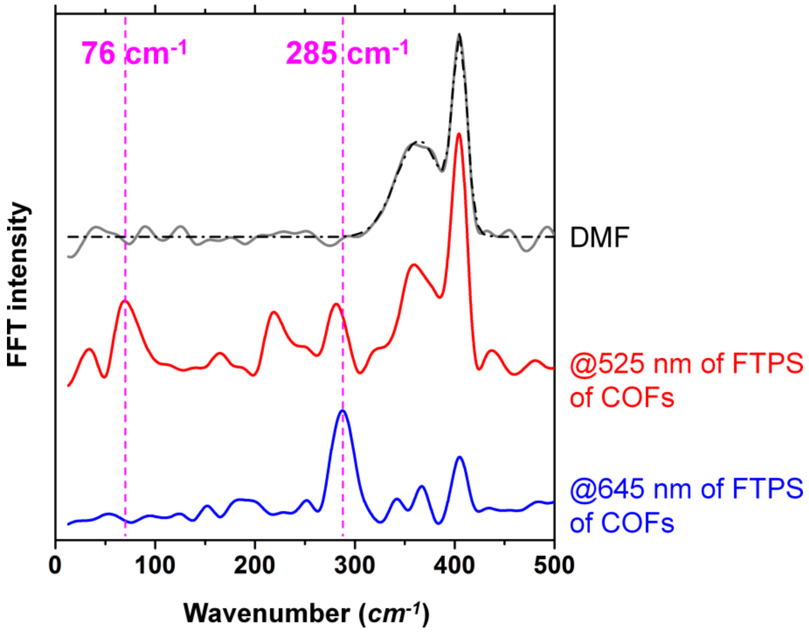

Supplementary Fig. 11. Wavelength-resolved Fourier transform power spectra (FTPS) for the oscillatory component of COFs at the selected probe wavelengths of 525 nm and 645 nm. The FTPS of pure solvent (DMF) shows a significant feature around 350 − 400 cm^{-1}, which is well matched with the theoretical spectrum (dashed line) reconstructed from two Gaussian functions centered at 364 cm^{-1} and 405 cm^{-1}. From the comparison of FTPS of COFs and pure solvent, it is evident that the FTPS spectra of COFs give rise to two prominent peaks of 76 cm^{-1} and 285 cm^{-1} in the low-frequency region, which is absent in the FTPS of pure solvent.

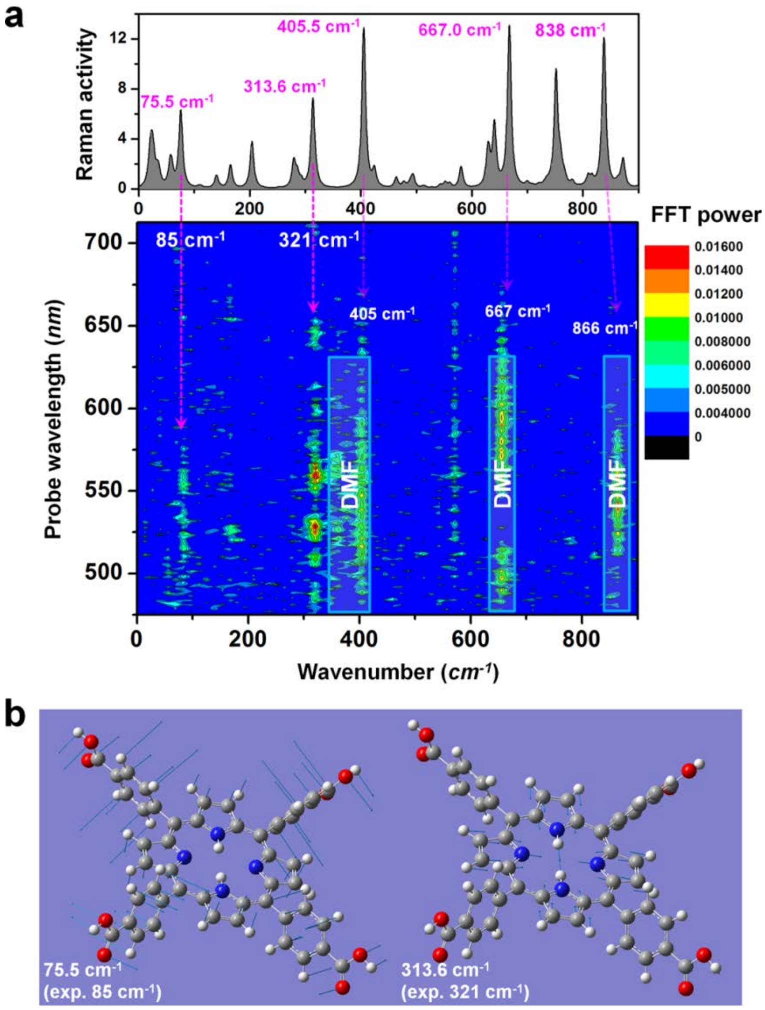

Supplementary Fig. 12. Wavelength-resolved Fourier transform power spectra (FTPS) of free-base porphyrin. (a) Contour map of wavelength-resolved FTPS in the range of 0 – 900 cm^{-1} at the probe spectral window of 475 nm – 710 nm. The vibrational coherences with the frequencies of 85 cm^{-1} and 321 cm^{-1} prominent in the low-frequency region arise from the solute, whereas the vibrational coherences of 405 cm^{-1}, 667 cm^{-1}, and 866 cm^{-1} frequencies arise from both the solvent and the solute. The calculated Raman spectrum of free-base porphyrin shown in upper panel is compared with the FTPS. The vibrational frequencies were calculated for the optimized structure with the D_{2h} symmetry using B3LYP/6-311G(d,p). (b) Calculated normal modes of 75.5 cm^{-1} and 313.6 cm^{-1} frequencies with the atomic displacements indicated (blue arrow). The 75.5 cm^{-1} mode mainly involves

torsional motion of side phenyl rings, while the 313.6 cm^{-1} mode corresponds to the in-plane motion of porphyrin ring.

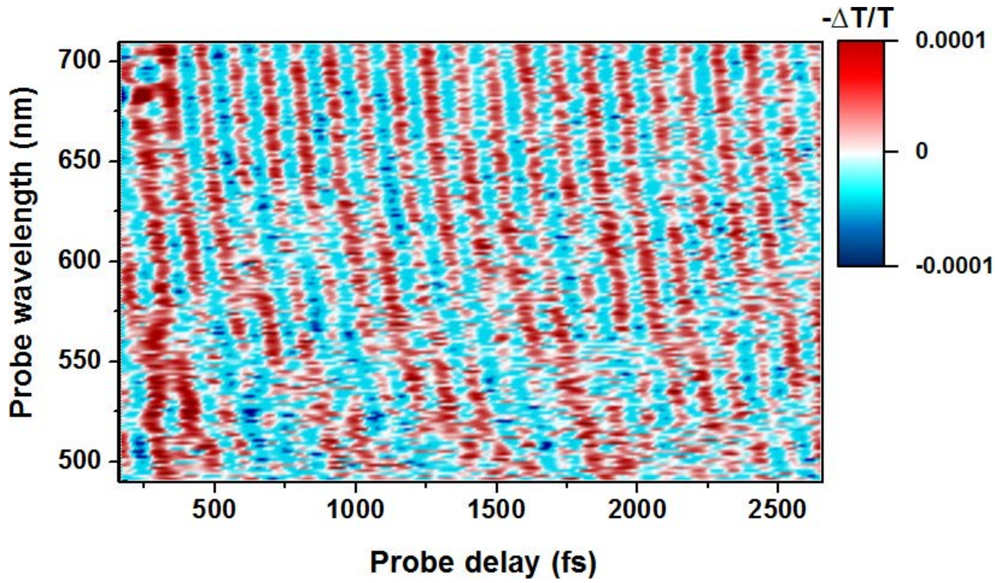

Supplementary Fig. 13. Two-dimensional plot of residuals obtained by subtracting the electronic population dynamics from the TA data of COFs. For clarity, the solvent contributions from the solvent (DMF) were filtered in the two-dimensional residual plot.

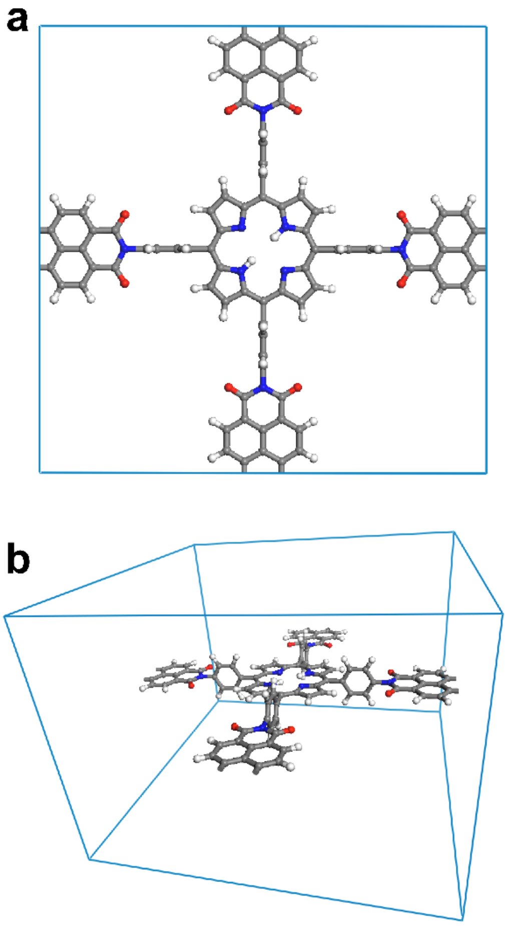

Supplementary Fig. 14. Periodic molecular structure in a unit cell. (a) Top and (b) perspective view of the unit cell of a COF monolayer consisting of 42 hydrogen, 92 carbon, 8 nitrogen, and 8 oxygen atoms.

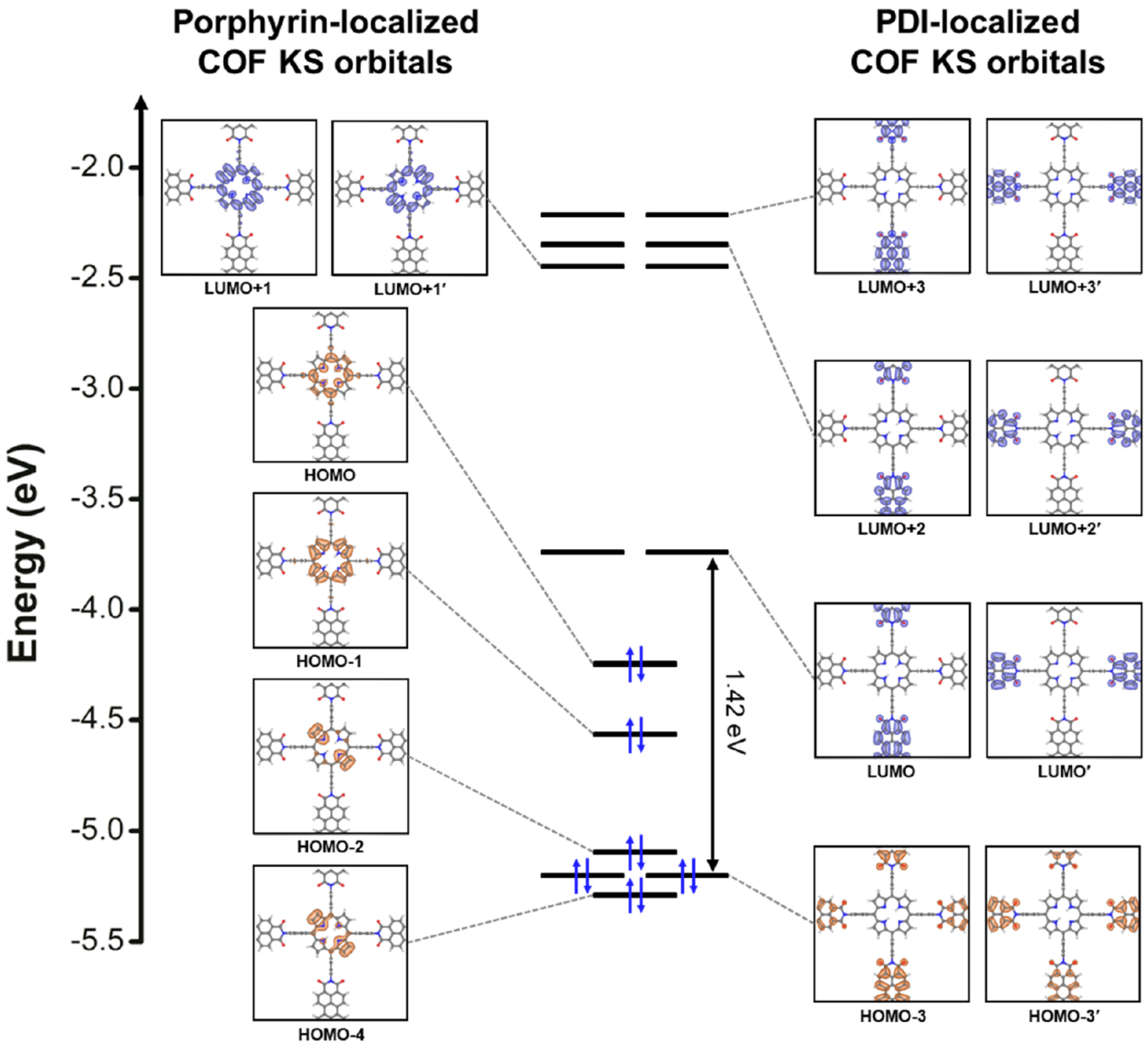

Supplementary Fig. 15. Calculated Kohn-Sham DFT energy levels of the COF unit cell ranging from HOMO-4 to LUMO+3′. Constituent orbitals localized at free-base porphyrin (left) and PDI (right) subunits were displayed on the sides with their isosurface images. Occupied KS levels were represented with pairwise up-down arrows. The computed DFT band gap of the PDI subunit was 1.42 eV.

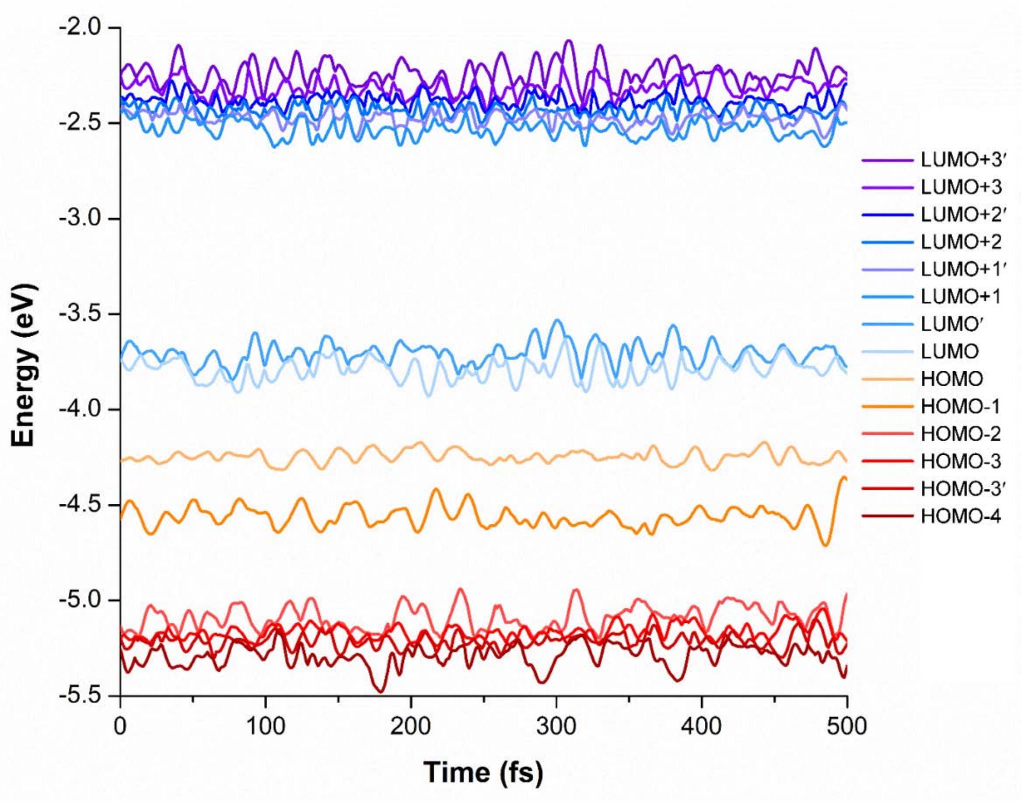

Supplementary Fig. 16. Time evolution of the computed Kohn-Sham DFT energy levels ranging from HOMO-4 to LUMO+3′ along the computed 500-fs trajectory.

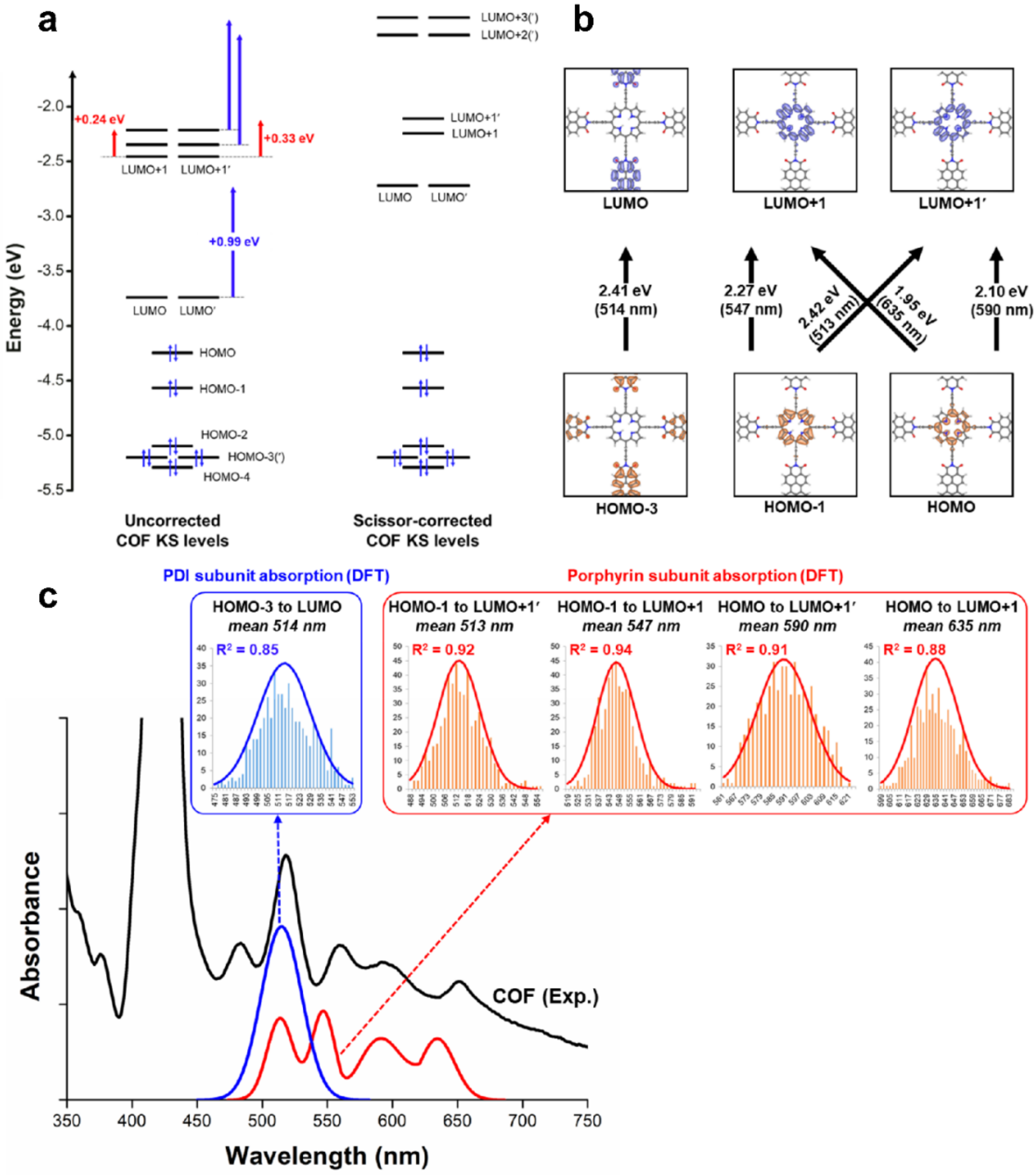

Supplementary Fig. 17. Linear absorption spectrum of COFs simulated by time-dependent DFT. (a) Scissor operation on KS-DFT energy levels of COFs. Rigid shift was applied to the KS states to reproduce the experimental energy gap. LUMO and LUMO′ localized at PDI subunit are upwardly shifted by 0.99 eV to match the experimental absorption at 514 nm, and LUMO+1 and LUMO+1′ are upwardly shifted by 0.24 eV and 0.33 eV, respectively, to match the experimental absorptions in the range of 513 – 635 nm. (b)

Scissor-corrected energy gaps of the subunits represented with the KS isosurface images. (c) Histograms of energy gaps of KS-DFT levels show a reasonable spectral feature compared with the experimental linear absorption spectrum in the range of 500 – 635 nm after applying the scissor operations. The histograms were fitted by Gaussian functions and were shown with a determination coefficient (R^2) indicating the fit quality. The value of R^2 closer to 1 means a perfect fit.

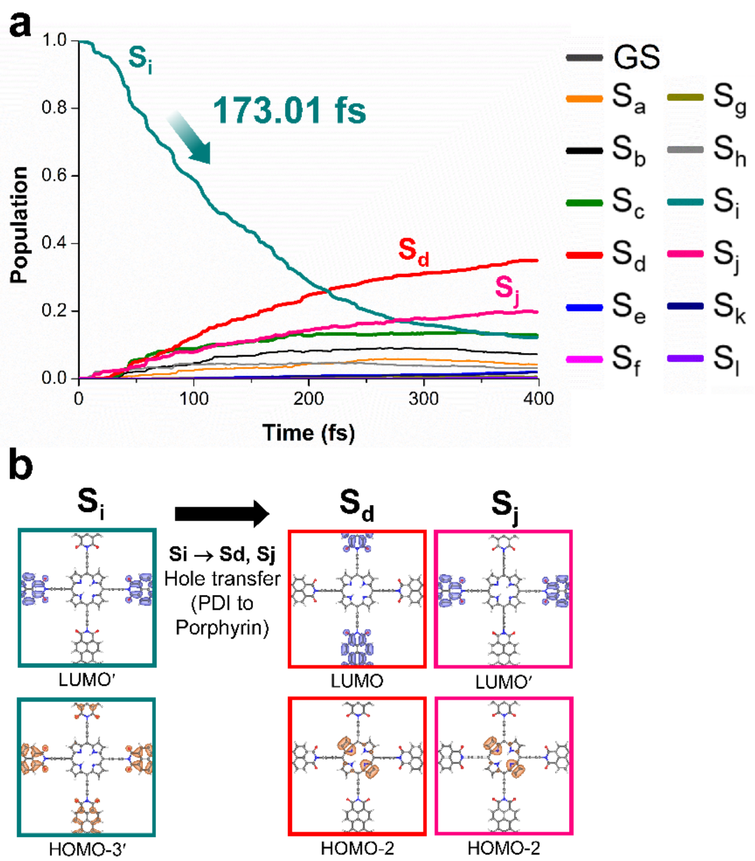

Supplementary Fig. 18. *Ab initio* Non-adiabatic molecular dynamics simulations for photoexcited charges carrier. (a) Time-evolution of population dynamics after an initial excitation into S_i state. The time constant for the population decay was obtained by using an exponential fit. (b) Evolution of electron-hole distribution starting from the initially excited S_i state, visualized with the Kohn-Sham (KS) orbitals of COFs.

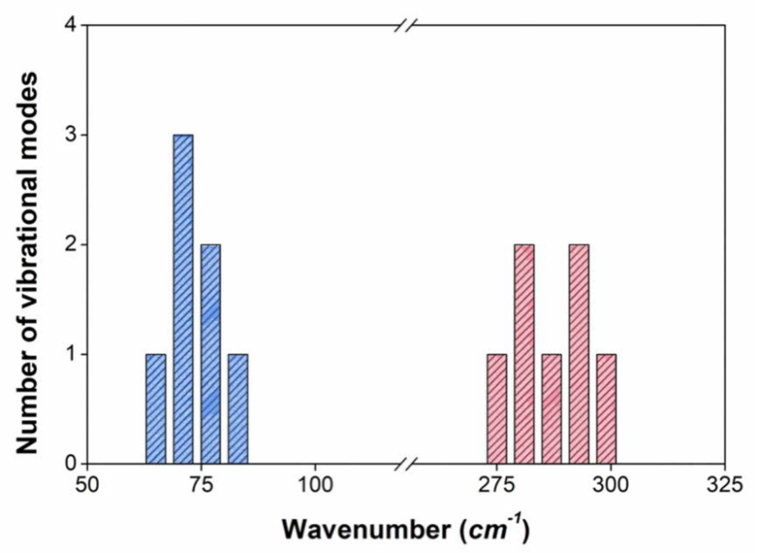

Supplementary Fig. 19. Histogram of calculated vibrational modes around 76 cm^{-1} and 285 cm^{-1} frequencies.

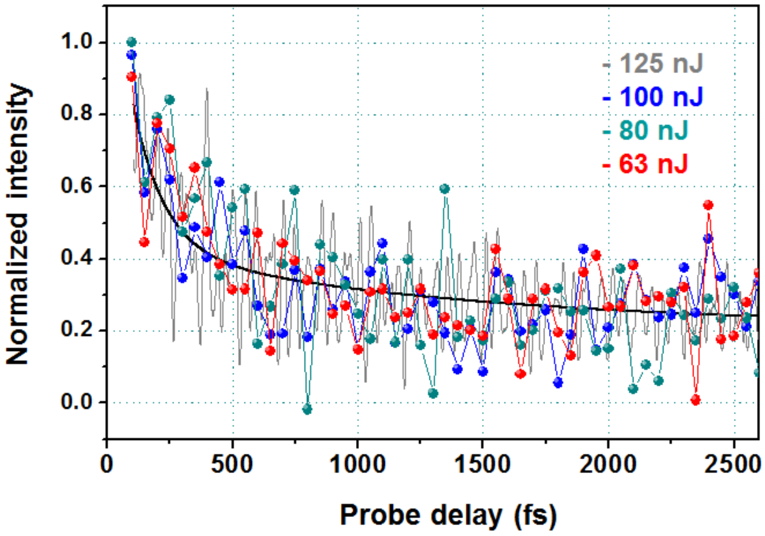

Supplementary Fig. 20. Excitation energy dependence of TA signal. Time profiles of TA intensity at the probe wavelength of 550 nm are shown as a function of excitation fluence at 523 nm. For the fluence of 125 nJ, the time interval between pump and probe pulses is 8 fs while, for the other cases (100 nJ, 80 nJ, and 63 nJ), the time interval of 50 fs was used. The bi-exponential fit (black line) with the time constants of 124 fs and 1.25 ps obtained from the global kinetic analysis is shown together for comparison. The TA signal at the probe wavelength of 550 nm mainly consists of stimulated emission feature of a single excited state. Since the decay dynamics of TA intensity is not dependent on the excitation fluence, we excluded the possibility of the recovery of the excited-state population back to the ground state in the time window of TA measurement.

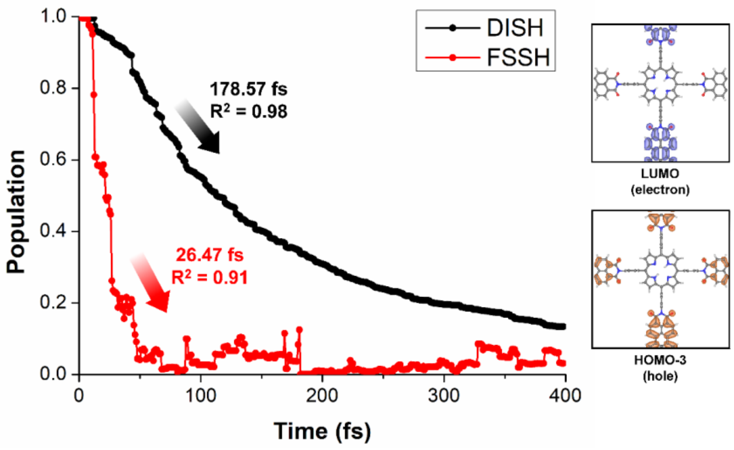

Supplementary Fig. 21. Time evolution of the surface hopping populations of the electron-hole pair states localized in the PDI subunit. These profiles were obtained from the NA-MD simulations using the FSSH and DISH schemes under the excitation of 2.4 eV.

Supplementary Table 1. List of indexes of the singly excited electron-hole pairs in the active space of the KS-DFT orbital ranging from the HOMO-4 to LUMO+1′.

Excited state	Hole	Electron
S_a	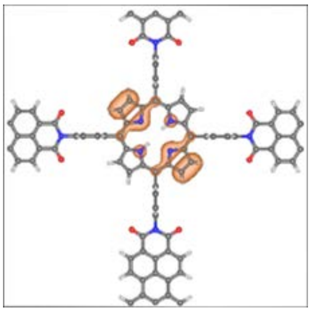 HOMO-4	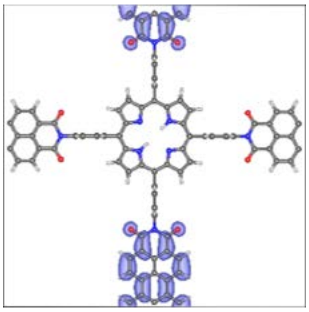 LUMO
S_b	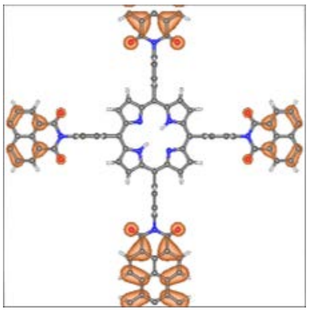 HOMO-3	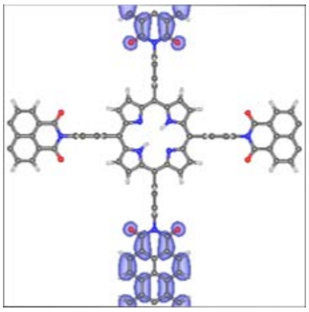 LUMO
S_c	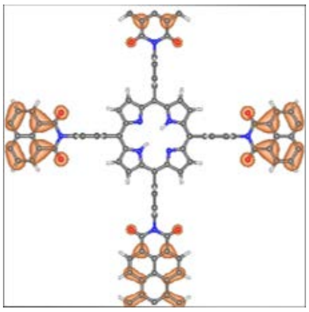 HOMO-3′	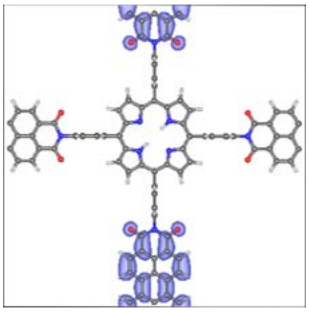 LUMO

S_d	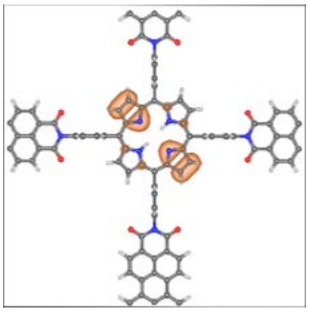 HOMO-2	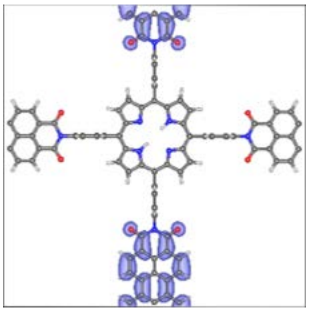 LUMO
S_e	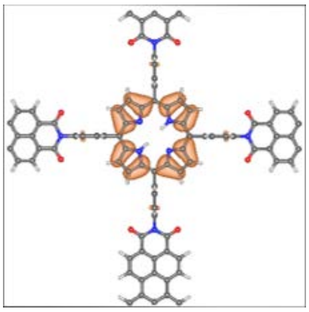 HOMO-1	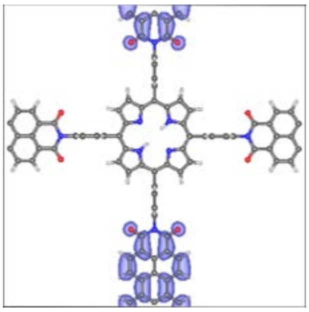 LUMO
S_f	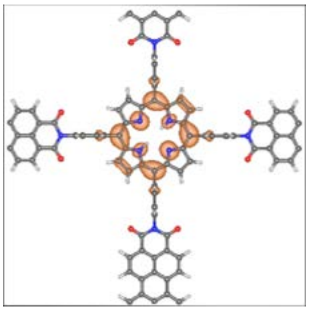 HOMO	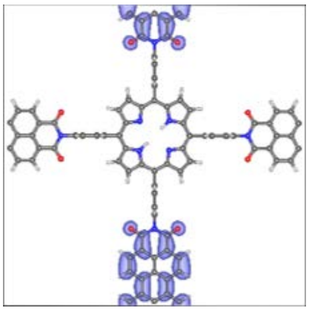 LUMO
S_g	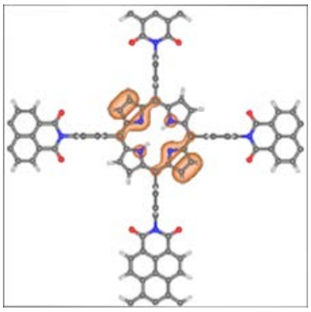 HOMO-4	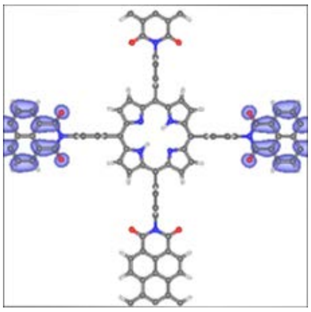 LUMO′

S_h	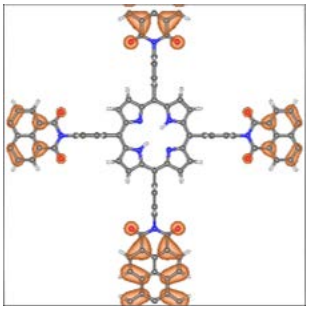 HOMO-3	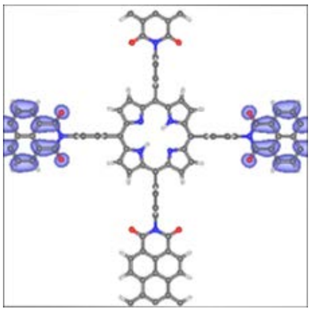 LUMO′
S_i	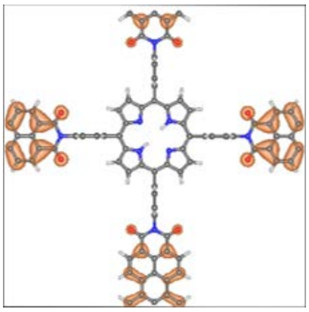 HOMO-3′	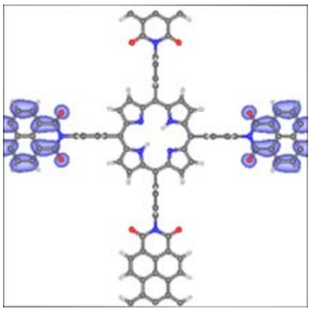 LUMO′
S_j	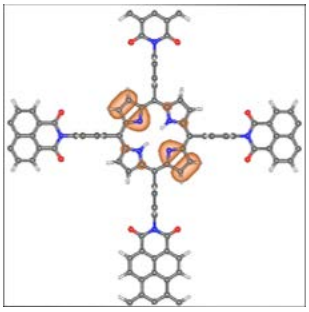 HOMO-2	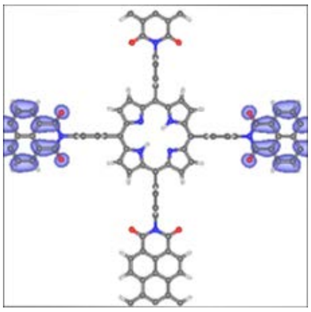 LUMO′
S_k	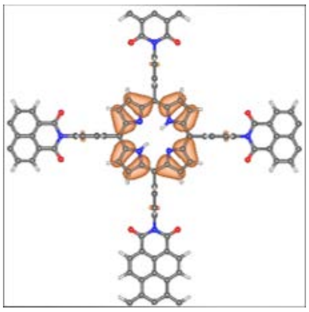 HOMO-1	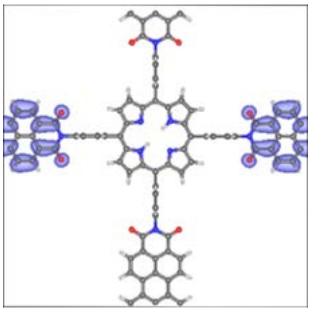 LUMO′

S_l	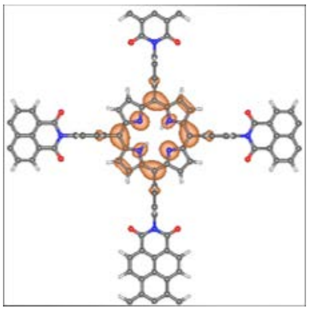 HOMO	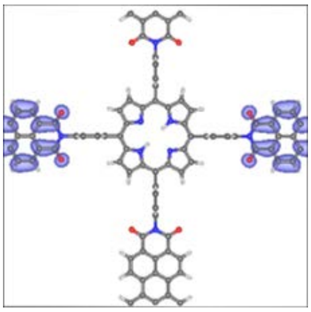 LUMO′
S_m	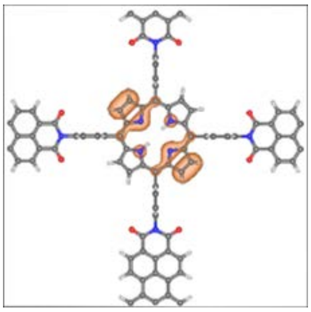 HOMO-4	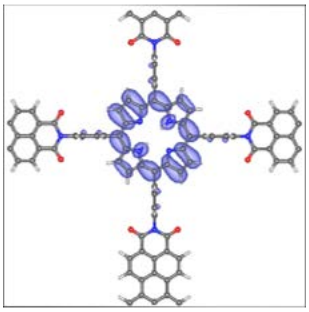 LUMO+1
S_n	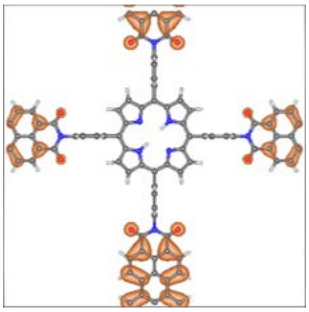 HOMO-3	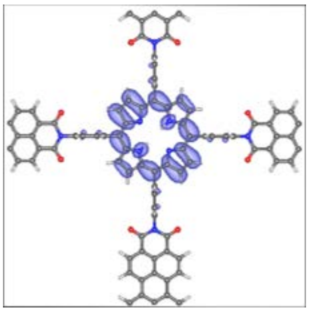 LUMO+1
S_o	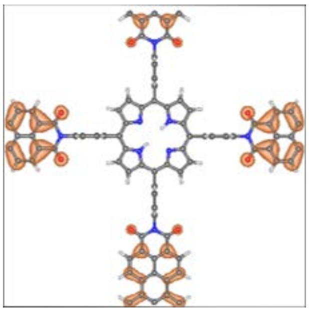 HOMO-3′	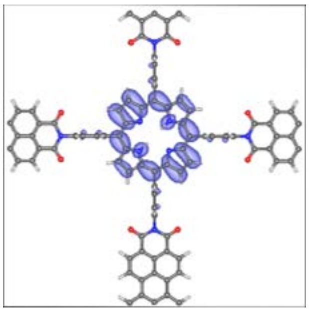 LUMO+1

S_p	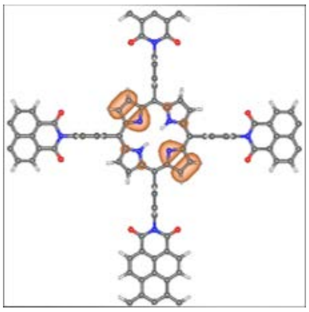<br>HOMO-2	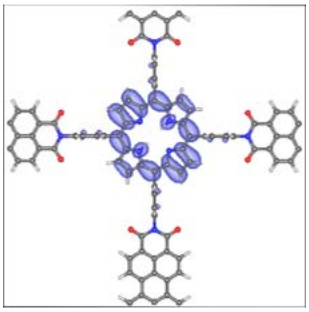<br>LUMO+1
S_q	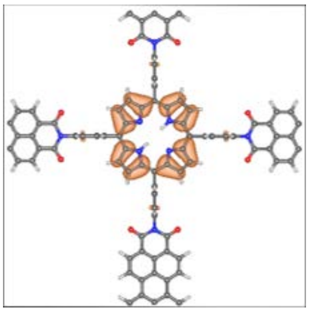<br>HOMO-1	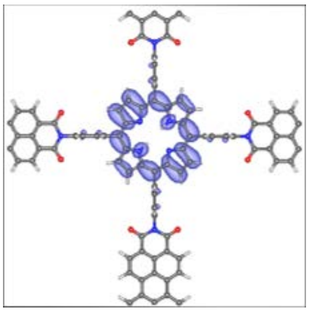<br>LUMO+1
S_r	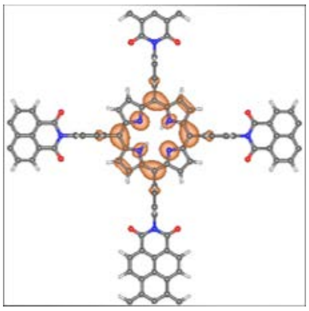<br>HOMO	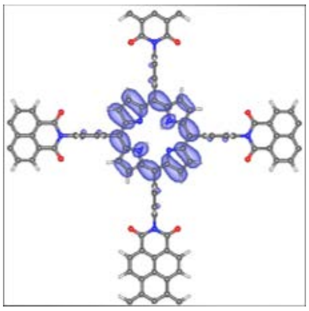<br>LUMO+1
S_s	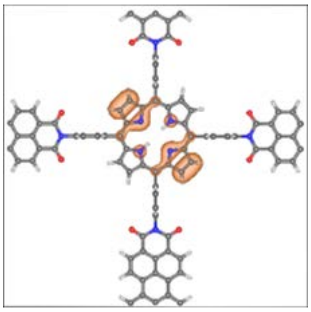<br>HOMO-4	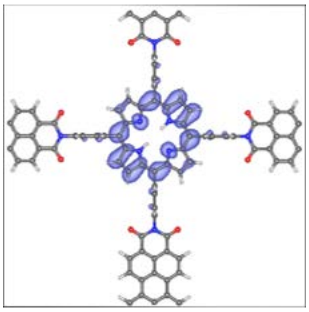<br>LUMO+1′

S_t	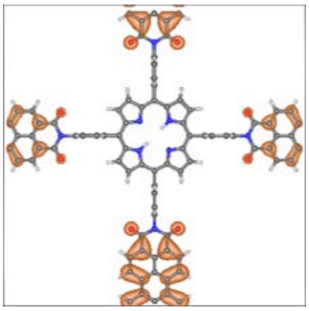<br>HOMO-3	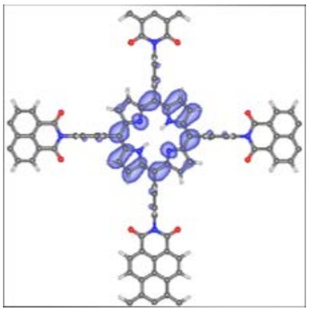<br>LUMO+1′
S_u	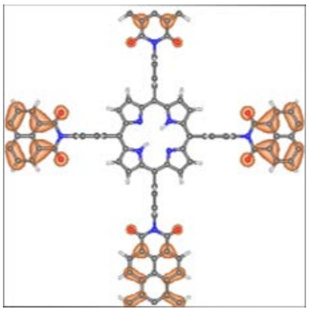<br>HOMO-3′	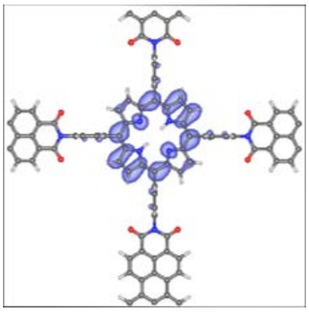<br>LUMO+1′
S_v	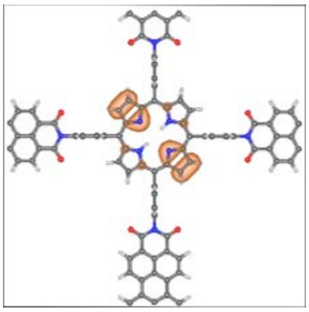<br>HOMO-2	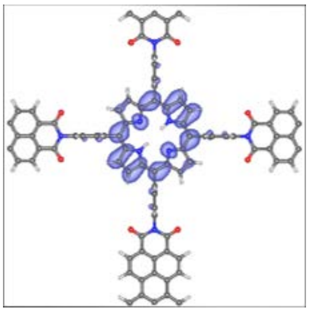<br>LUMO+1′
S_w	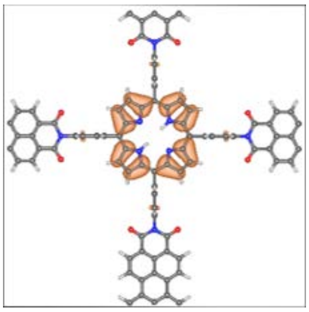<br>HOMO-1	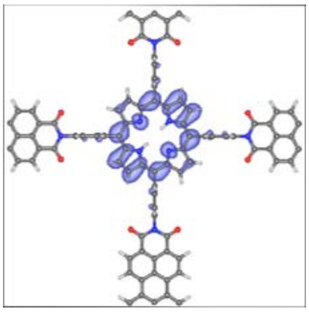<br>LUMO+1′

S_x	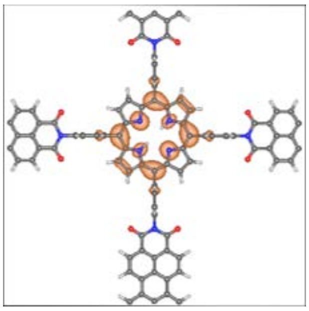 HOMO	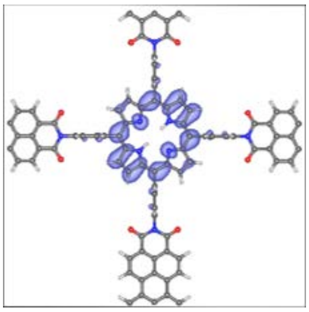 LUMO+1′

Supplementary Table 2. Populations of singly-excited states that were sampled 400 fs after the excitation into S_b, S_d, and S_i states, which are sampled using surface hopping dynamics of NA-MD simulations.

Initial excited state	Electron hole-pair	Population at 400 fs (%)
S_b	GS	0.00
	S_a	3.32
	S_b	13.40
	S_c	13.12
	S_d	36.44
	S_e	2.44
	S_f	0.28
	S_g	0.50
	S_h	2.96
	S_i	7.22
	S_j	18.24
	S_k	1.62
	S_l	0.46
S_d	GS	0.02
	S_a	0.38
	S_b	1.74

	S_c	5.22
	S_d	78.42
	S_e	1.74
	S_f	0.52
	S_g	0.06
	S_h	0.40
	S_i	2.28
	S_j	7.40
	S_k	1.38
	S_l	0.44
S_i	GS	0.04
	S_a	4.26
	S_b	7.24
	S_c	12.88
	S_d	35.00
	S_e	2.10
	S_f	0.40
	S_g	0.68
	S_h	3.18
	S_i	12.24
	S_j	19.78

	S_k	1.80
	S_l	0.40

Supplementary Table 3. List of the vibrational normal modes of COFs around 76 cm^{-1} and 285 cm^{-1} frequencies.

**Wavenumber (cm^{-1})**
64.58
70.12
72.89
73.01
74.02
77.12
82.86
273.62
279.69
282.08
286.26
291.86
295.03
297.58

Supplementary Table 4. Time constants for the population decays of excited states after the PDI-centered excitation (from HOMO-3 to LUMO) depending on various ranges of active space. These time constants were determined from the NA-MD simulations using the DISH algorithm under the excitation energy of 2.4 eV.

Active space	# of active spaces	Time constant (fs)	R^2
HOMO-7 ~ LUMO′	11	179.53	0.97
HOMO-7 ~ LUMO+1′	13	177.30	0.98
HOMO-7 ~ LUMO+2′	15	176.06	0.98
HOMO-7 ~ LUMO+3′	17	173.01	0.97
HOMO-7 ~ LUMO+4′	19	177.30	0.97
HOMO-4 ~ LUMO′	8	181.49	0.98
HOMO-4 ~ LUMO+1′	10	178.57	0.98
HOMO-4 ~ LUMO+2′	12	180.51	0.99
HOMO-4 ~ LUMO+3′	14	182.15	0.99
HOMO-4 ~ LUMO+4′	16	180.83	0.98
HOMO-3 ~ LUMO′	7	215.52	0.97
HOMO-3 ~ LUMO+1′	9	221.24	0.97
HOMO-3 ~ LUMO+2′	11	219.30	0.96
HOMO-3 ~ LUMO+3′	13	216.92	0.97
HOMO-3 ~ LUMO+4′	15	224.72	0.97

Supplementary References

1 Bettelheim, A., White, B. A., Raybuck, S. A. & Murray, R. W. Electrochemical Polymerization of Amino-Substituted, Pyrrole-Substituted, and Hydroxy-Substituted Tetraphenylporphyrins. *Inorg. Chem.* **26**, 1009-1017 (1987).

2 Karimipour, G., Kowkabi, S. & Naghiha, A. New Aminoporphyrins Bearing Urea Derivative Substituents: Synthesis, Characterization, Antibacterial and Antifungal Activity. *Braz. Arch. Biol. Technol.* **58**, 431-442 (2015).

3 Feng, X. *et al.* High-Rate Charge-Carrier Transport in Porphyrin Covalent Organic Frameworks: Switching from Hole to Electron to Ambipolar Conduction. *Angew. Chem. Int. Ed.* **51**, 2618-2622 (2012).

4 Jin, S. B. *et al.* Charge Dynamics in A Donor-Acceptor Covalent Organic Framework with Periodically Ordered Bicontinuous Heterojunctions. *Angew. Chem. Int. Ed.* **52**, 2017-2021 (2013).

5 Fang, Q. R. *et al.* Designed synthesis of large-pore crystalline polyimide covalent organic frameworks. *Nat. Commun.* **5** (2014).

6 Haase, F. *et al.* Tuning the stacking behaviour of a 2D covalent organic framework through non-covalent interactions. *Mat. Chem. Front.* **1**, 1354-1361 (2017).

7 Shao, Y. Y. *et al.* Nitrogen-doped graphene and its electrochemical applications. *J. Mater. Chem.* **20**, 7491-7496 (2010).

8 Ren, S. J. *et al.* Porous, Fluorescent, Covalent Triazine-Based Frameworks Via Room-Temperature and Microwave-Assisted Synthesis. *Adv. Mater.* **24**, 2357-2361 (2012).

9 Yu, S. B. *et al.* A polycationic covalent organic framework: a robust adsorbent for anionic dye pollutants. *Polym. Chem.* **7**, 3392-3397 (2016).

10 Spitler, E. L. & Dichtel, W. R. Lewis acid-catalysed formation of two-dimensional phthalocyanine covalent organic frameworks. *Nat. Chem.* **2**, 672-677 (2010).

11 Qian, H. L., Yang, C. X. & Yan, X. P. Bottom-up synthesis of chiral covalent organic frameworks and their bound capillaries for chiral separation. *Nat. Commun.* **7** (2016).

12 Kresse, G. & Furthmuller, J. Efficient iterative schemes for ab initio total-energy calculations using a plane-wave basis set. *Phys. Rev. B* **54**, 11169-11186 (1996).

13 Kresse, G. & Furthmuller, J. Efficiency of ab-initio total energy calculations for metals and semiconductors using a plane-wave basis set. *Comp. Mater. Sci.* **6**, 15-50 (1996).

14 Blochl, P. E. Projector Augmented-Wave Method. *Phys. Rev. B* **50**, 17953-17979 (1994).

15 Izumi, F. & Momma, K. Three-Dimensional Visualization in Powder Diffraction. *Solid State Phenomena* **130**, 15-20 (2007).

16 Manson, J. E. Preferred Orientation of Platelets in X-Ray Diffractometer Powder Samples. *American Institute of Physics* **26**, 1254 (1955).

17 Vazquez-Molina, D. A. *et al.* Mechanically Shaped Two-Dimensional Covalent Organic Frameworks Reveal Crystallographic Alignment and Fast Li-Ion Conductivity. *J. Am. Chem. Soc.* **138**, 9767-9770 (2016).

18 Henry, E. R. & Hofrichter, J. Singular Value Decomposition - Application to Analysis of Experimental-Data. *Method Enzymol.* **210**, 129-192 (1992).

19 Lagarias, J. C., Reeds, J. A., Wright, M. H. & Wright, P. E. Convergence properties of the Nelder-Mead simplex method in low dimensions. *Siam. J. Optimiz.* **9**, 112-147 (1998).

20 Venkata Chalapathi, V. & Venkata Ramiah, K. Normal vibrations of N, N-dimethylformamide and N, N-dimethylacetamide. *Proc. Indian. Acad. Sci. A* **68**, 109-122 (1968).
