# Supplementary material for: Ultrafast charge transfer coupled with lattice phonons in two-dimensional covalent organic frameworks
Source: Nat Commun. 2019 Apr 23;10:1873. doi: 10.1038/s41467-019-09872-w (PMC6478948; doi:10.1038/s41467-019-09872-w)
Supplement: Supplementary file 3 — Description of Additional Supplementary Files [file 41467_2019_9872_MOESM3_ESM.docx]

**Description of Additional Supplementary Files**

File Name: Supplementary Data 1
Description: Calculated normal mode of 70.12 cm-1 frequency.

File Name: Supplementary Data 2
Description: Calculated normal mode of 72.89 cm-1 frequency.

File Name: Supplementary Data 3
Description: Calculated normal mode of 73.01 cm-1 frequency.

File Name: Supplementary Data 4
Description: Calculated normal mode of 74.02 cm-1 frequency.

File Name: Supplementary Data 5
Description: Calculated normal mode of 77.12 cm-1 frequency.

File Name: Supplementary Data 6
Description: Calculated normal mode of 82.86 cm-1 frequency.

File Name: Supplementary Data 7
Description: Calculated normal mode of 273.62 cm-1 frequency.

File Name: Supplementary Data 8
Description: Calculated normal mode of 279.69 cm-1 frequency.

File Name: Supplementary Data 9
Description: Calculated normal mode of 282.08 cm-1 frequency.

File Name: Supplementary Data 10
Description: Calculated normal mode of 286.26 cm-1 frequency.

File Name: Supplementary Data 11
Description: Calculated normal mode of 291.86 cm-1 frequency.

File Name: Supplementary Data 12
Description: Calculated normal mode of 295.03 cm-1 frequency.

File Name: Supplementary Data 13
Description: Calculated normal mode of 297.58 cm-1 frequency.

File Name: Supplementary Data 14
Description: List of indexes of the singly excited electron-hole pairs in the active space of the KS-DFT orbital.
