# Supplementary figures and images for: Ultrafast charge transfer coupled with lattice phonons in two-dimensional covalent organic frameworks
Source: Nat Commun. 2019 Apr 23;10:1873. doi: 10.1038/s41467-019-09872-w (PMC6478948; doi:10.1038/s41467-019-09872-w)

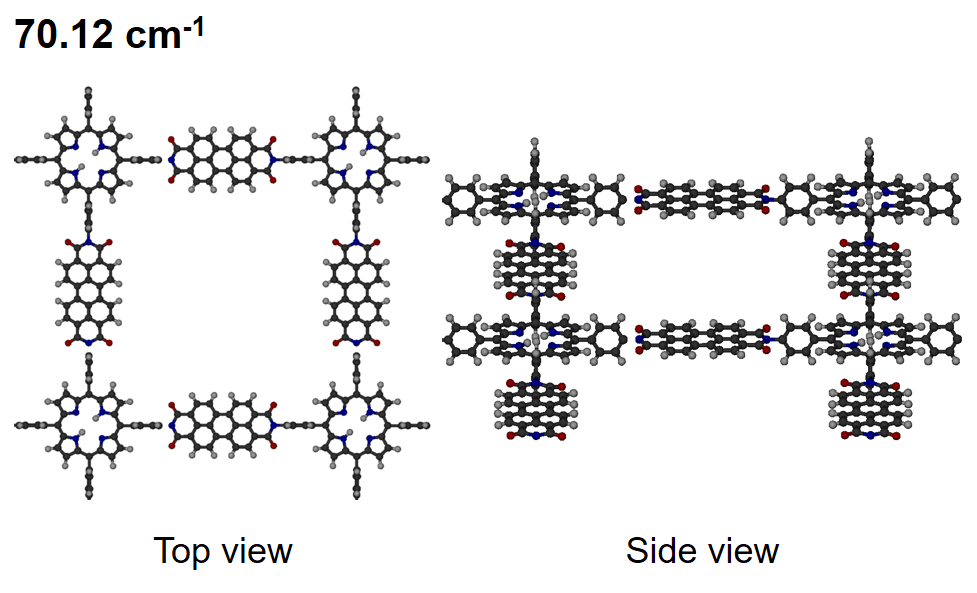

Supplement: Supplementary file 4 — Supplementary Data 1 [file 41467_2019_9872_MOESM4_ESM.gif]

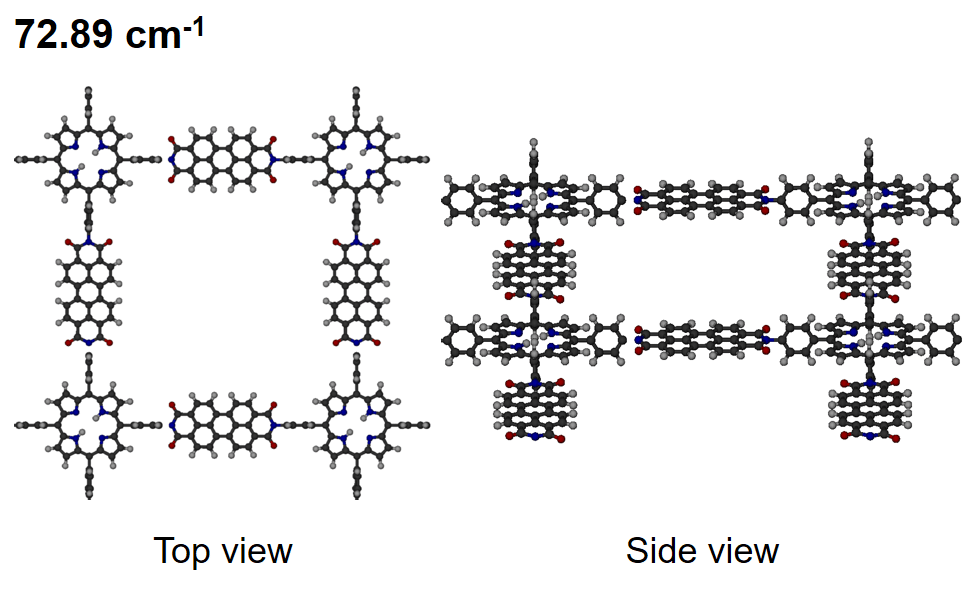

Supplement: Supplementary file 5 — Supplementary Data 2 [file 41467_2019_9872_MOESM5_ESM.gif]

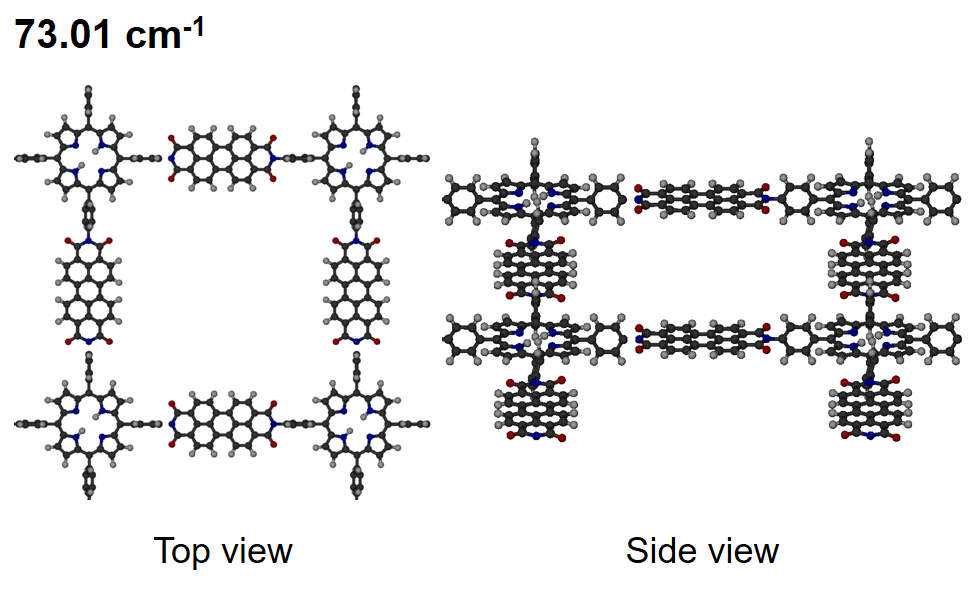

Supplement: Supplementary file 6 — Supplementary Data 3 [file 41467_2019_9872_MOESM6_ESM.gif]

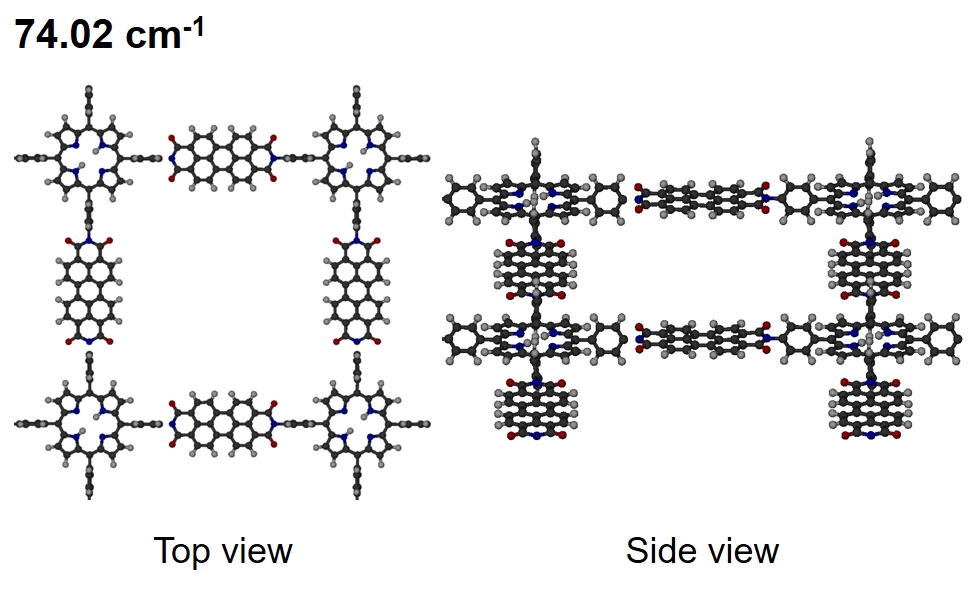

Supplement: Supplementary file 7 — Supplementary Data 4 [file 41467_2019_9872_MOESM7_ESM.gif]

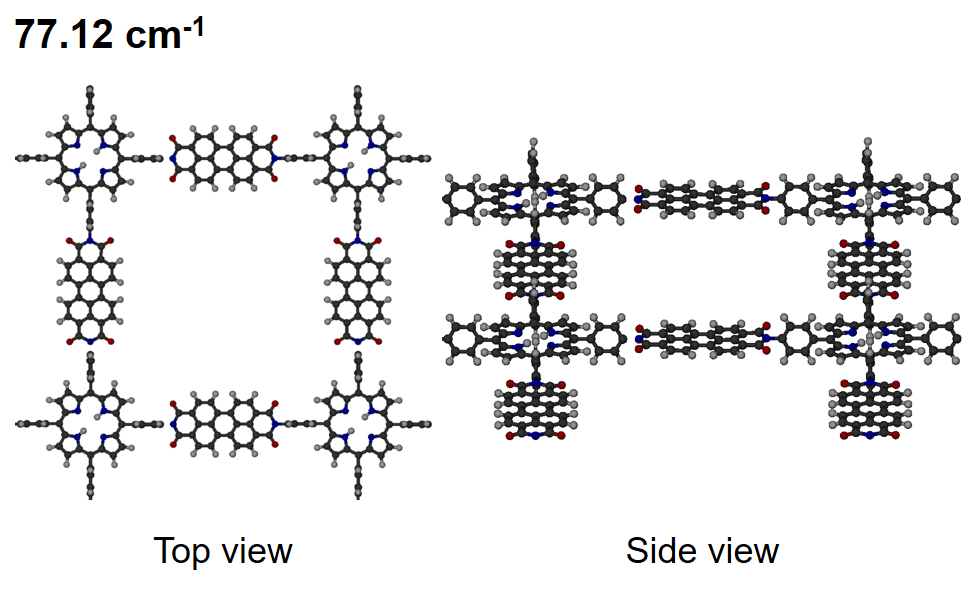

Supplement: Supplementary file 8 — Supplementary Data 5 [file 41467_2019_9872_MOESM8_ESM.gif]

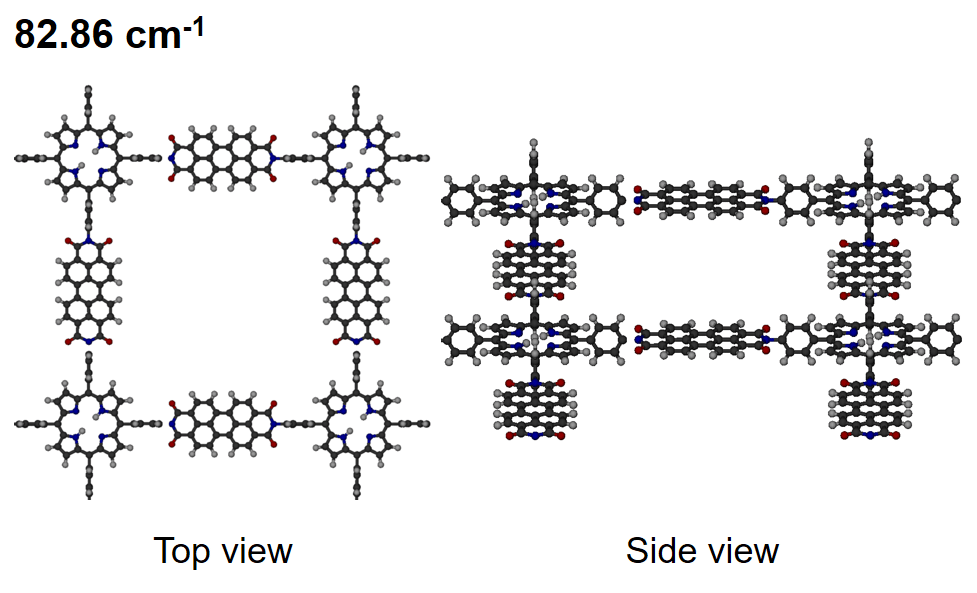

Supplement: Supplementary file 9 — Supplementary Data 6 [file 41467_2019_9872_MOESM9_ESM.gif]

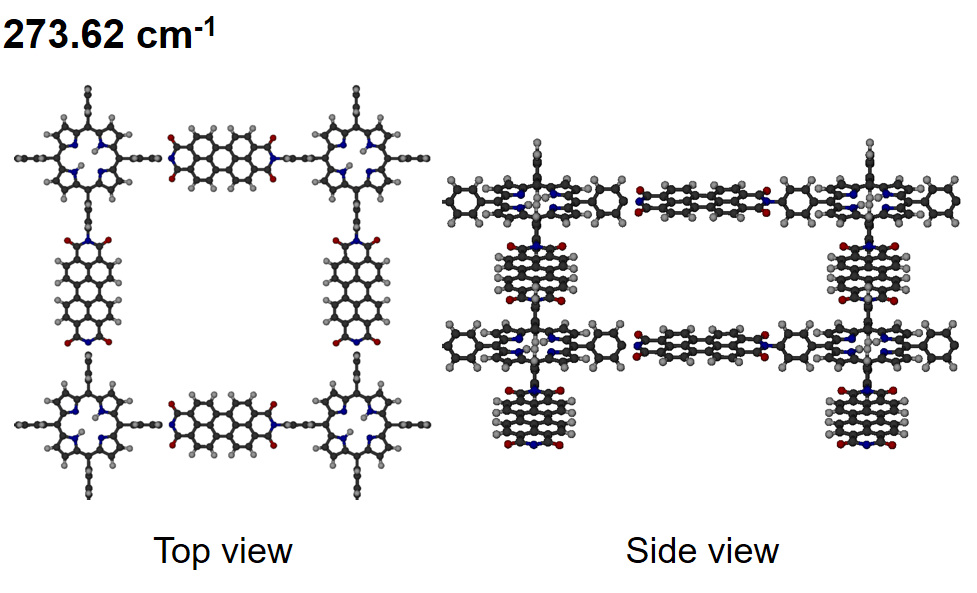

Supplement: Supplementary file 10 — Supplementary Data 7 [file 41467_2019_9872_MOESM10_ESM.gif]

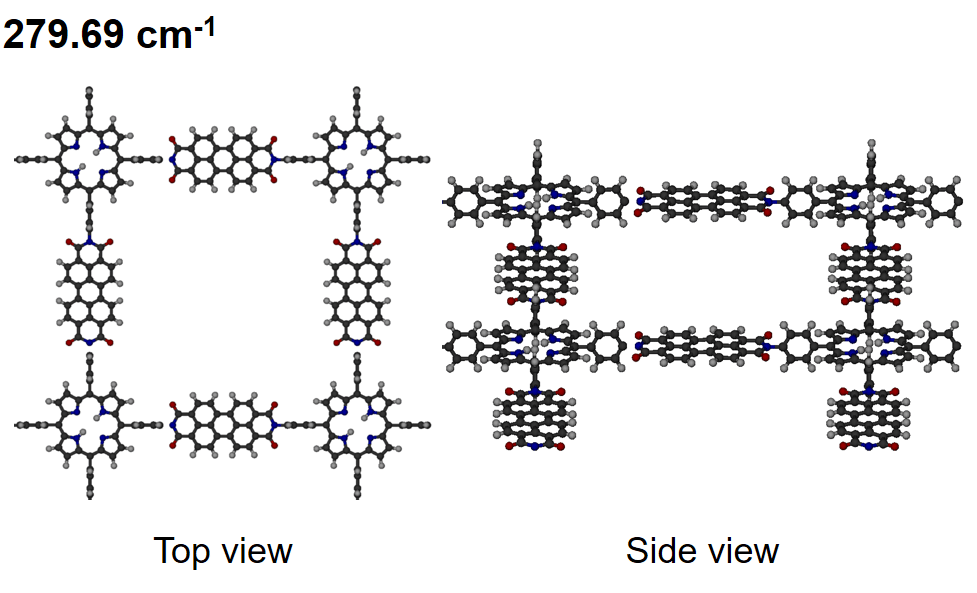

Supplement: Supplementary file 11 — Supplementary Data 8 [file 41467_2019_9872_MOESM11_ESM.gif]

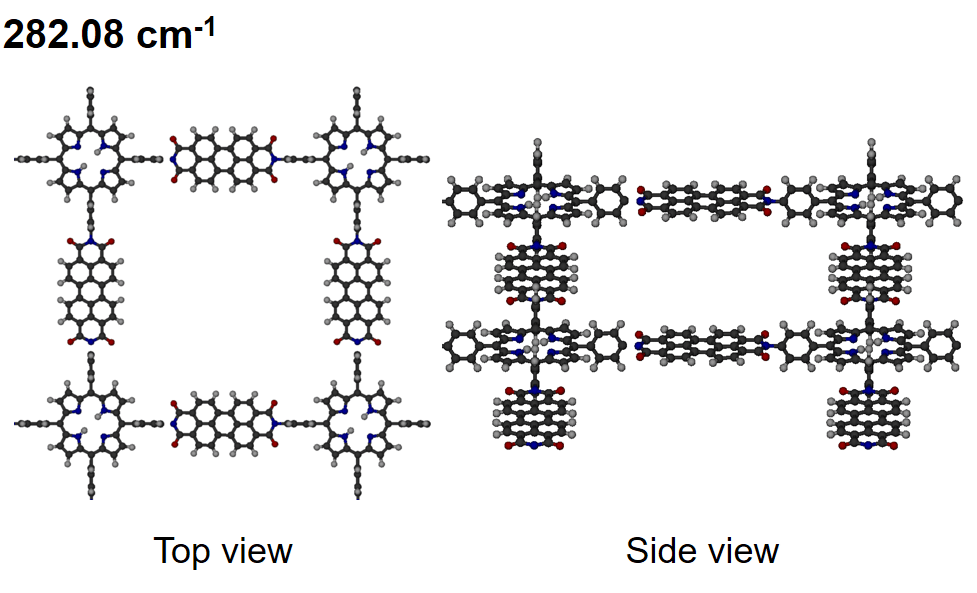

Supplement: Supplementary file 12 — Supplementary Data 9 [file 41467_2019_9872_MOESM12_ESM.gif]

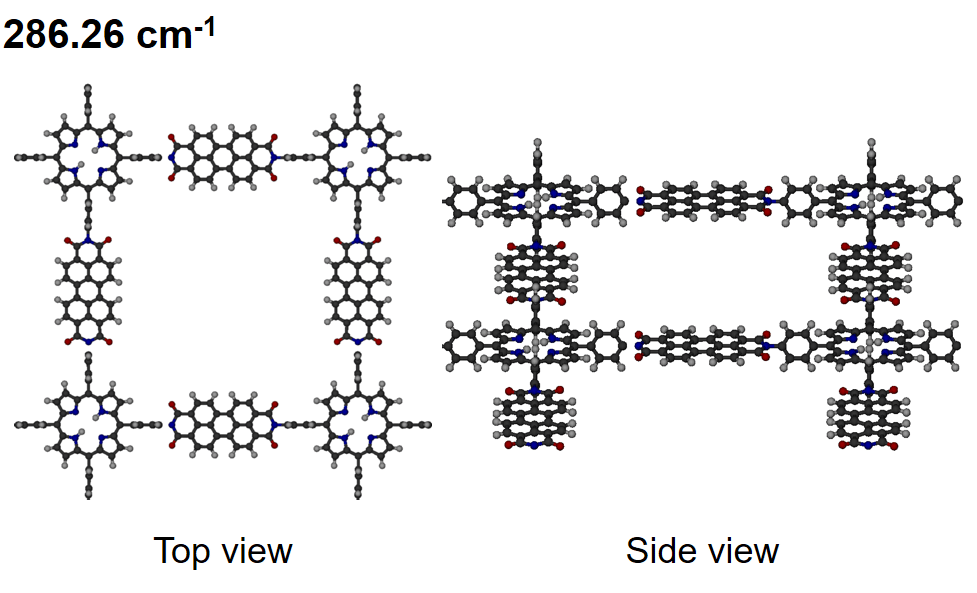

Supplement: Supplementary file 13 — Supplementary Data 10 [file 41467_2019_9872_MOESM13_ESM.gif]

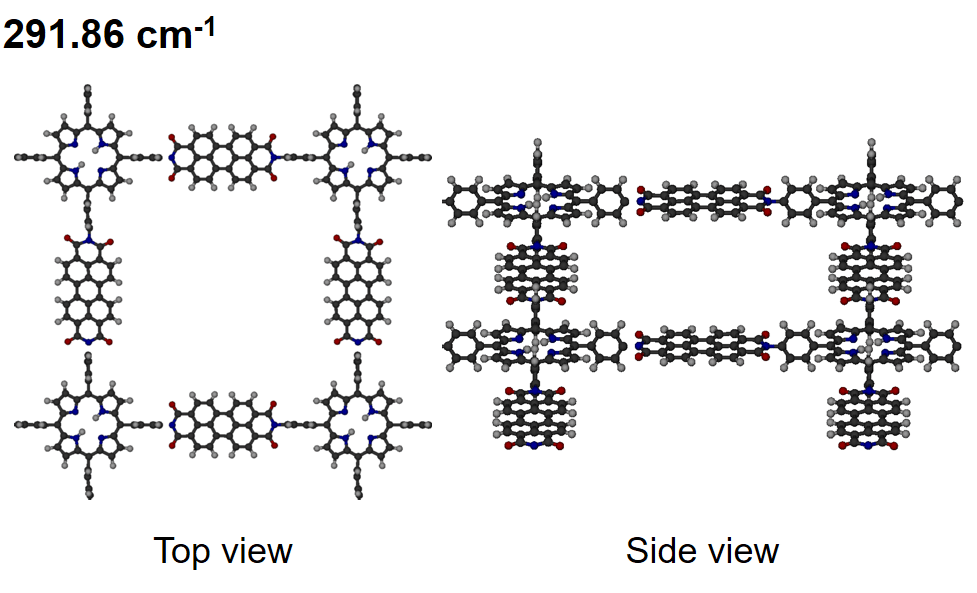

Supplement: Supplementary file 14 — Supplementary Data 11 [file 41467_2019_9872_MOESM14_ESM.gif]

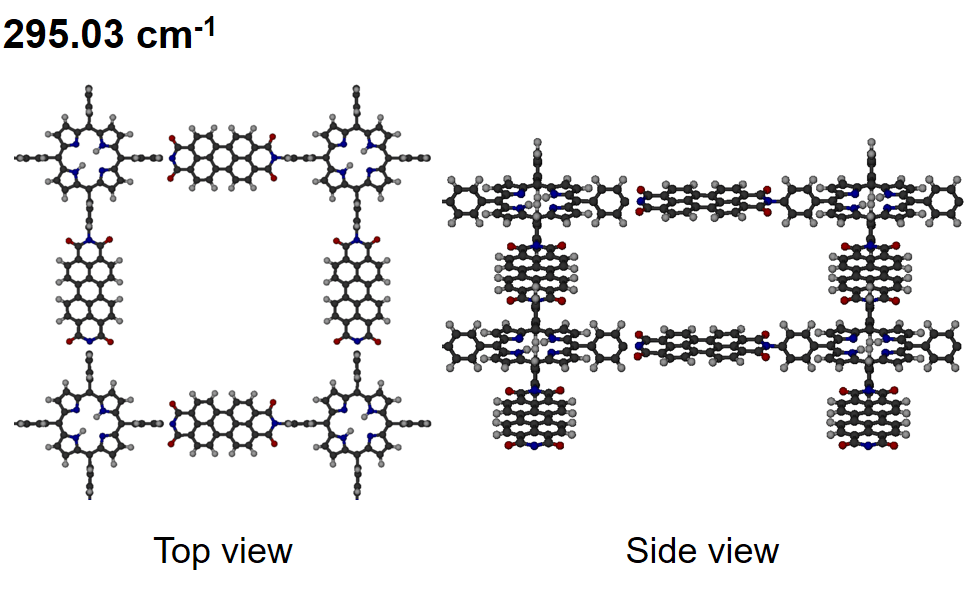

Supplement: Supplementary file 15 — Supplementary Data 12 [file 41467_2019_9872_MOESM15_ESM.gif]

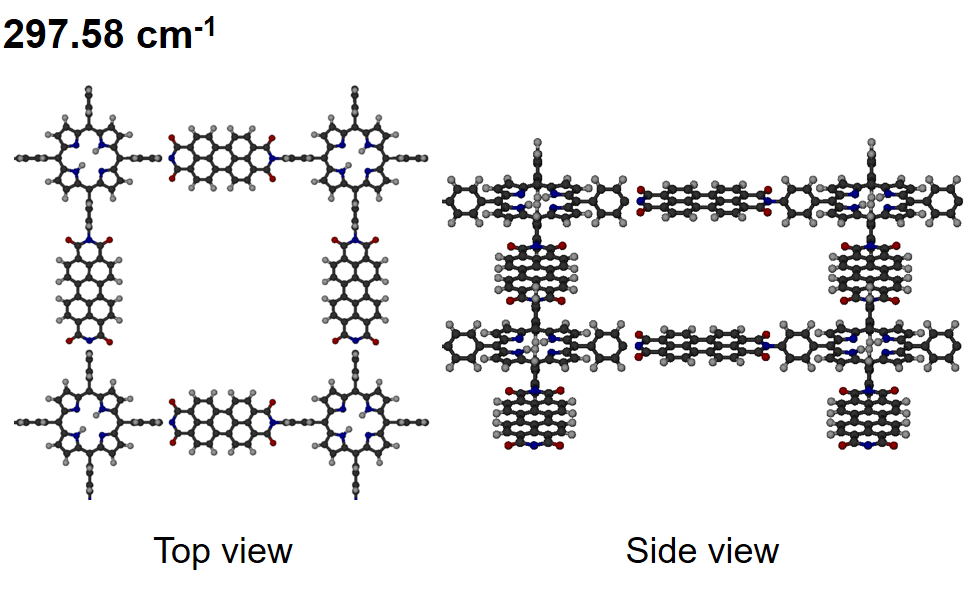

Supplement: Supplementary file 16 — Supplementary Data 13 [file 41467_2019_9872_MOESM16_ESM.gif]
