# Supplementary material for: Ultrafast charge transfer coupled with lattice phonons in two-dimensional covalent organic frameworks
Source: Nat Commun. 2019 Apr 23;10:1873. doi: 10.1038/s41467-019-09872-w (PMC6478948; doi:10.1038/s41467-019-09872-w)
Supplement: Supplementary file 17 — Supplementary Data 14 [file 41467_2019_9872_MOESM17_ESM.pdf]

Table S1. List of indexes of the singly excited electron-hole pairs in the active space of the KS-DFT orbital ranging from the HOMO-4 to LUMO+1′.

Excited state	Hole	Electron
S_a	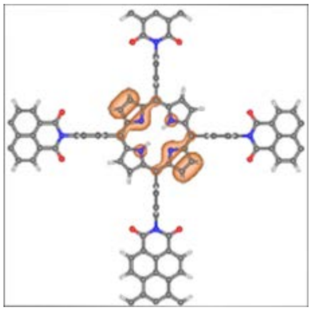 HOMO-4	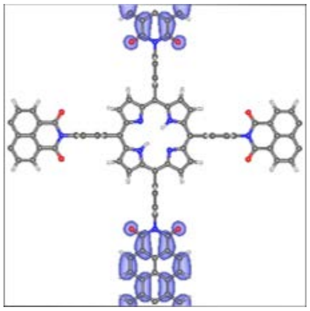 LUMO
S_b	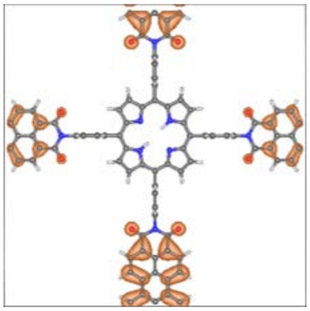 HOMO-3	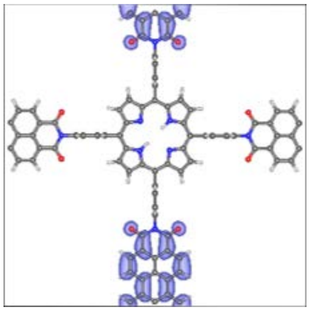 LUMO
S_c	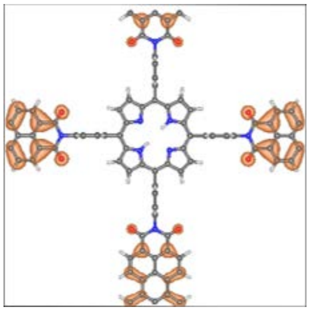 HOMO-3′	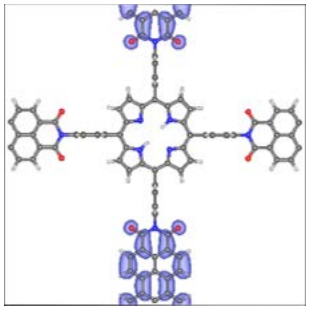 LUMO
S_d	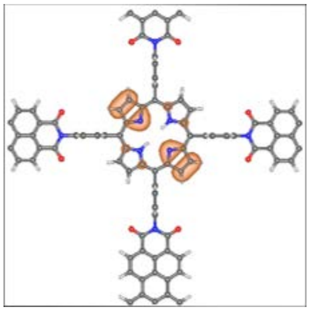 HOMO-2	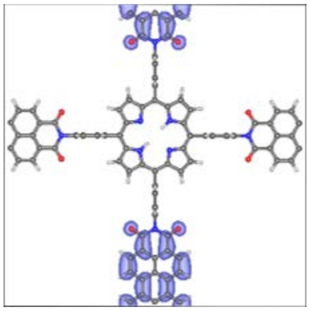 LUMO

S_e	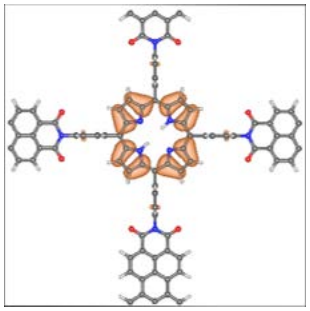<br>HOMO-1	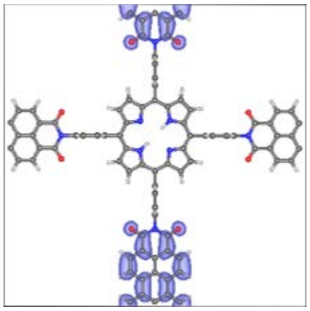<br>LUMO
S_f	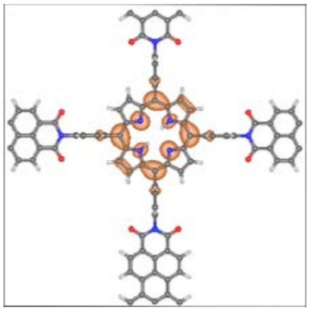<br>HOMO	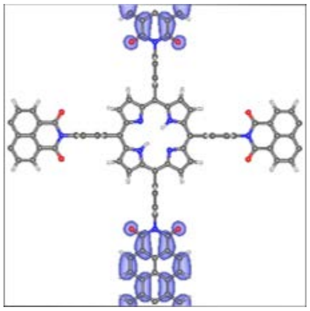<br>LUMO
S_g	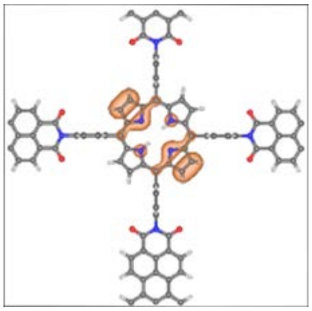<br>HOMO-4	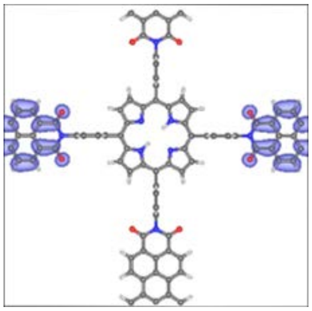<br>LUMO′
S_h	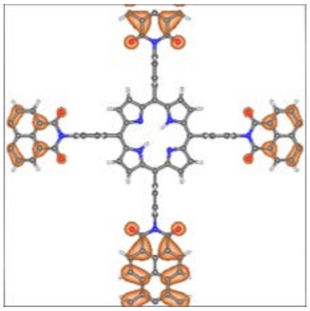<br>HOMO-3	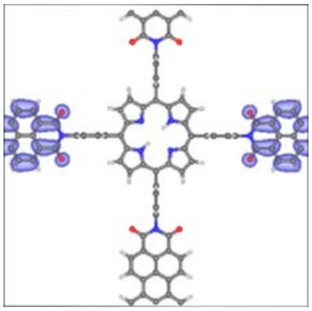<br>LUMO′

S_i	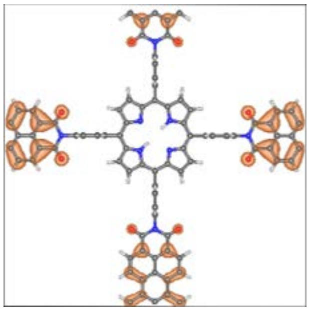<br>HOMO-3′	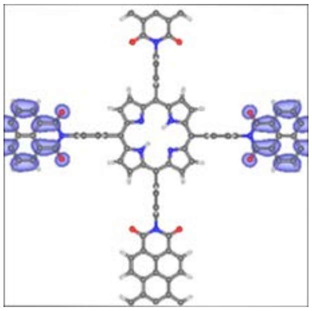<br>LUMO′
S_j	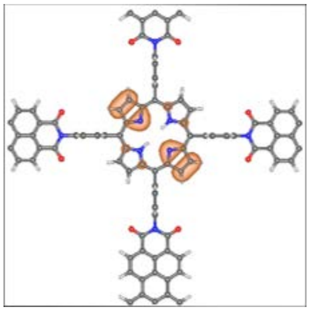<br>HOMO-2	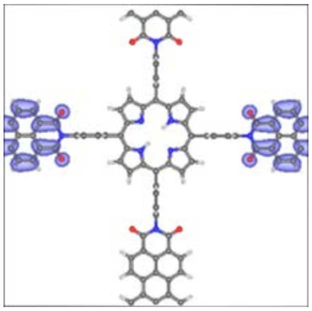<br>LUMO′
S_k	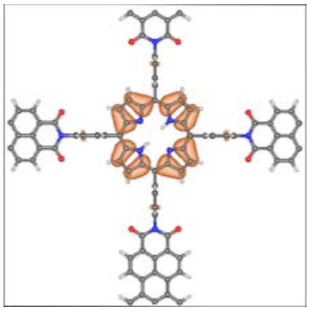<br>HOMO-1	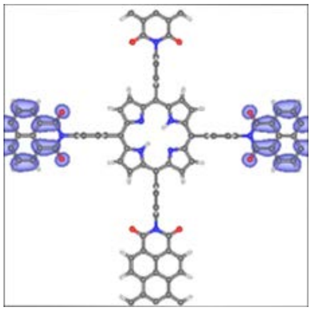<br>LUMO′
S_l	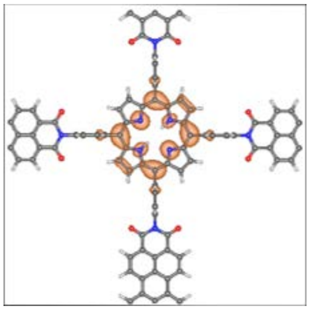<br>HOMO	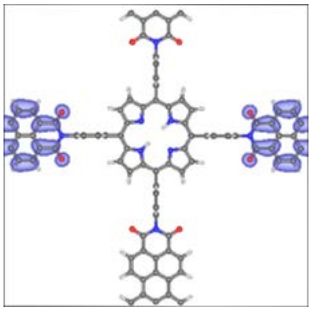<br>LUMO′

S_m	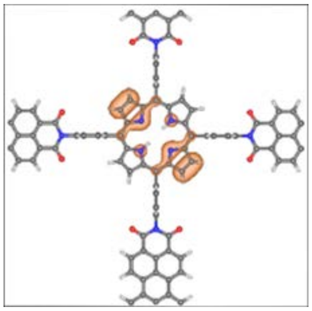<br>HOMO-4	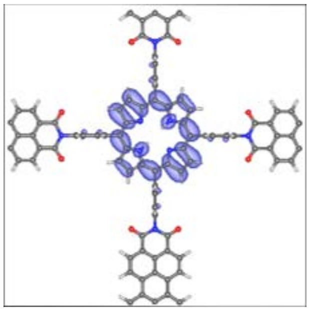<br>LUMO+1
S_n	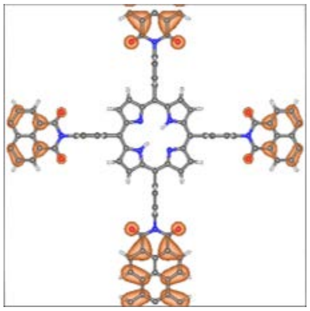<br>HOMO-3	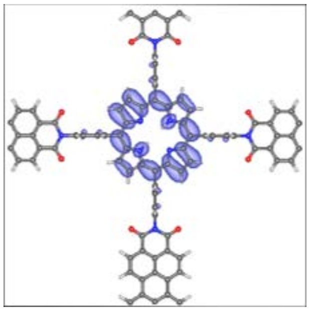<br>LUMO+1
S_o	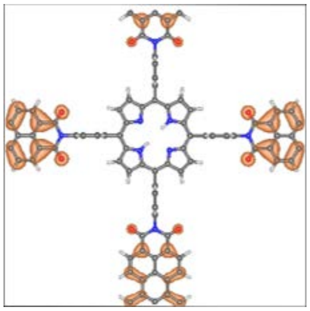<br>HOMO-3′	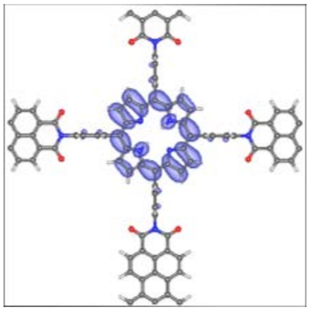<br>LUMO+1
S_p	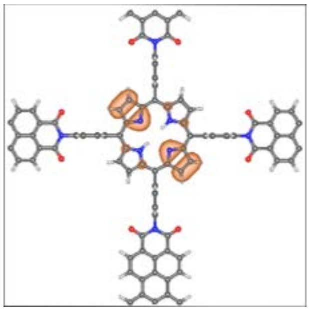<br>HOMO-2	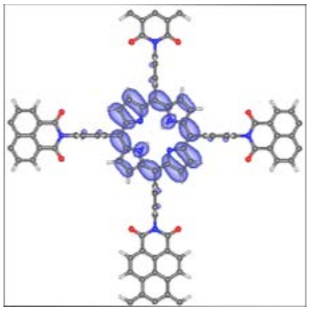<br>LUMO+1

S_q	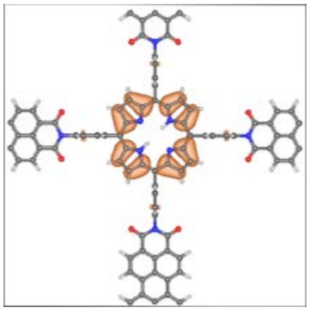<br>HOMO-1	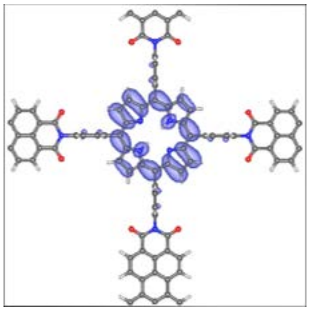<br>LUMO+1
S_r	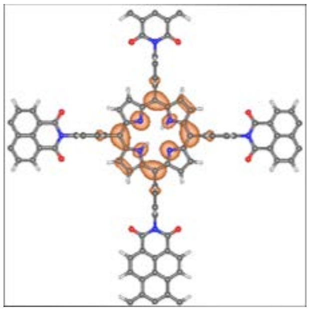<br>HOMO	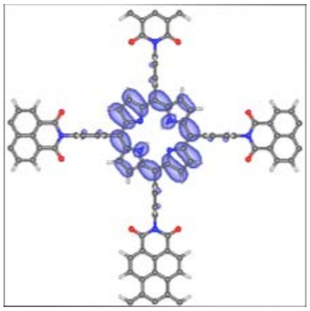<br>LUMO+1
S_s	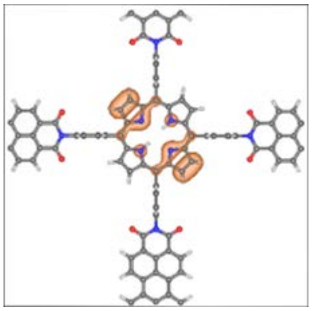<br>HOMO-4	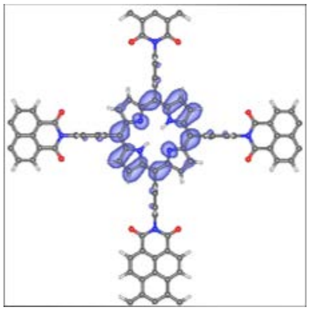<br>LUMO+1′
S_t	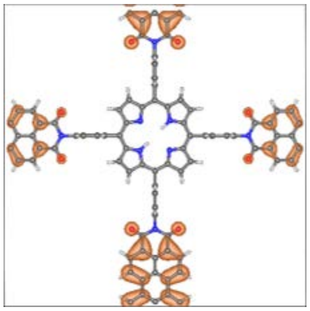<br>HOMO-3	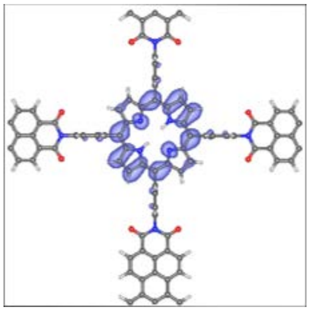<br>LUMO+1′

S_u	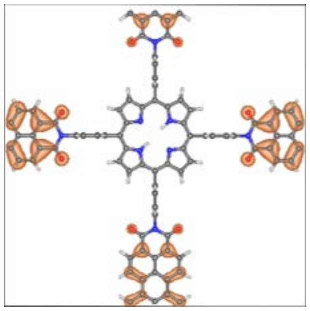<br>HOMO-3′	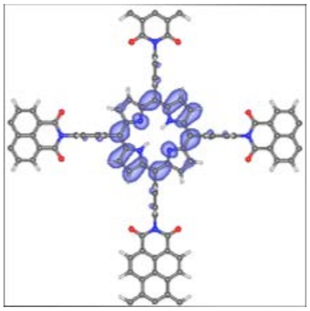<br>LUMO+1′
S_v	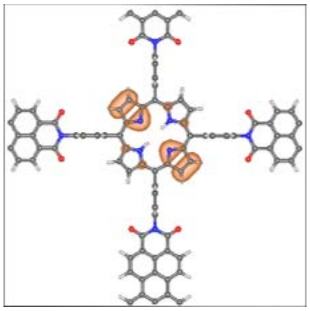<br>HOMO-2	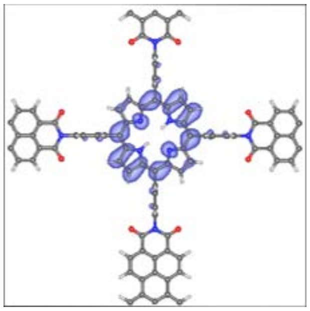<br>LUMO+1′
S_w	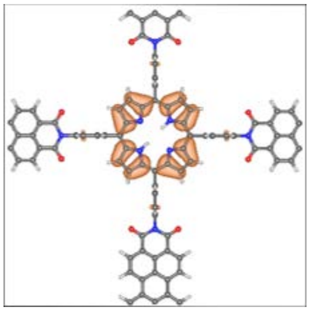<br>HOMO-1	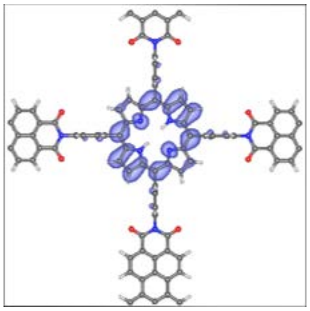<br>LUMO+1′
S_x	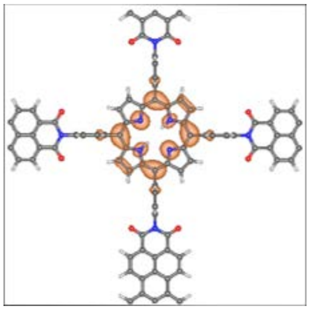<br>HOMO	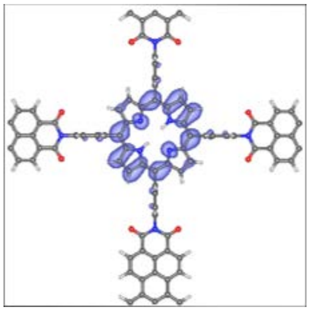<br>LUMO+1′
