# Supplementary material for: Ultrafast charge transfer coupled with lattice phonons in two-dimensional covalent organic frameworks
Source: Nat Commun. 2019 Apr 23;10:1873. doi: 10.1038/s41467-019-09872-w (PMC6478948; doi:10.1038/s41467-019-09872-w)
Supplement: Supplementary file 2 — Peer Review File [file 41467_2019_9872_MOESM2_ESM.pdf]

Reviewers' comments:

Reviewer #1 (Remarks to the Author):

The ms reports on the fs-dynamics of photogenerated charge carriers in novel 2D D-A COFs by combining femtosecond optical spectroscopy and non-adiabatic molecular dynamics simulation. The findings are novel and well supported by theory, and should be of interest of to the community working in COF and other comparable materials for optoelectronic applications. The work is convincing except in few places which I detail bellow. The work is very interesting, but I am not sure that "it will influence thinking in the field". But, there only very few reports using ultrafast spectroscopy to interrogate COFs, MOFs and HOFs. The ms is reasonably well written (except that the Figures of S.I are cited according to the flow of information). The ms merits its publication in Nature Communication after considering the following points:
Specific comments:

1- P5, bottom: in the sentence "The electronic interaction between PDI and free-base porphyrin units is manifested as spectral red shift and broadening of the absorption spectrum of COFs compared with the absorption spectrum of freebase porphyrin, as shown in Figure 1b.", I have few comments:
a – the shift of the band at 420 nm could be the result of scatter from the sample. The other bands at longer wavelengths of absorption, far from scatter, do NOT show any shift). In any case, if the shift is not from scattering, the authors should precise that they refer to the 420 nm band, and to all the bands.

b- It seems that the shape of the COF band in the inset of Figure 1 (almost without intensity at 440-450 nm !) is different form the one shown in the main figure (with the absorption of porphyrin.

2- The excitation at 523 nm of the COF pumps both porphyrin and PDI moieties. The authors should consider this possibility and its implication in the observed dynamics.

3- The authors cite figure S14 (p. 6) before all the figures S1-S13. I see also that several figures in the SI are not cited/used in the ms.

4- The negative signal from 500 to 560 nm, should contain a contribution from the excitation beam (523 nm) (p. 6).

5- For the assignment of the dynamics (P. 6), using a stepwise process, the authors were too fast. Furthermore, if "A" is the initially excited species, we cannot label it as an intermediate. The intermediate is B.

6- The error in the time constant of the first step is too optimistic, taking into account the IRF of the full setup (70 fs).

7- Show in SI a comparison of the spectra to believe the following argument; (p. 7): As can be seen in Figure 2b, the 1st SADS shows strong GSB and SE bands in the spectral region from 490 to 625 nm and these spectral features are in good agreement with the steady-state absorption/emission spectra and broadband TA spectrum of PDI monomers reported previously26-28.

8- As I said, the excitation at 523 nm will bring both unit to their excited states, and therefore, the

authors should reconsider the following statement in p. 7: "Thus, the intermediate A must be associated with PDI units of 2D COFs." Again "A" is not an intermediate.

9- P. 7: The authors state that "The SE is quenched further with the transition from B to C". I really do not see a significant decrease in the SE intensity.

10- P7: indicate the longest time delay of the setup.

11- Again Figure S6b is cited in the text before citing Figs. S1-S5.

12- A figure for comparison should be added in the SI to support the following: "When comparing the SADS of free-base porphyrin with its steady-state emission spectrum (Figure S6b), we can infer that the change of SADS at 610 − 710 nm is likely to be associated with the growth of SE contribution arising from the Qx(0,0) state of free-base porphyrin, which can be reached by Qy-to-Qx internal conversion and vibrational relaxation, as reported previously23,29,30."

13- The following sentence should be considered taking into account that the 523 nm excitation pulse, also excites the porphyrin unit. "Based on the results obtained from the TA measurements on COFs and free-base porphyrin, in the TA spectra of COFs, the quenching of SE at 490 – 625 nm arising from PDI units concurs with the growth of SE at 645 nm arising from free-base porphyrin units. Thus, the A → B transition of COFs with the time constant of 124 fs can be attributed to the ultrafast transfer of excited charge carriers from PDI to free-base porphyrin."

14- As the authors have made the experiment in DMF, it will be interesting to examine the solvent effect in the theoretical results.

My major point is: The reached/excited species of this COF using 523 nm. At this wavelength (figure 1) both units of the COF absorb and then excited. So, the authors should reconsider their analysis and assignment of the observed times. The COF dynamics should be compared to both of porphyrin (alone as it si done) and of PDI (which is lacking) in DMF solutions.

Reviewer #2 (Remarks to the Author):

In this manuscript, all of the calculations and hypotheses are based on the formation of 2D COFs. However, the authors did not provide enough evidence to prove the formation of 2D COF. Since the spectroscopy data are sensitive to the impurities and the packing mode of the resulting materials, these data are meaningless if they cannot confirm the purity of the resultant COF. Therefore, I do not recommend the publication of this manuscript.

1. The gas sorption data and pore size distribution analysis are missing. It's a standard procedure to characterize the structures of COFs. In addition, it's suggested to provide theoretical BET surface area and compared with the experimental data.

2. Judging from the PXRD patterns shown in Fig. S2, it's more likely that crystals of oligomers or linear polymers rather than COFs are formed. The simulated PXRD patterns are completely different compared with the experimental data.

3. The elemental analysis data of PDI-porphyrin COF should be provided.

4. The spectroscopy data of corresponding physical mixtures are suggested to be provided.

Responses to the comments from the reviewer #1

The ms reports on the fs-dynamics of photogenerated charge carriers in novel 2D D-A COFs by combining femtosecond optical spectroscopy and non-adiabatic molecular dynamics simulation. The findings are novel and well supported by theory, and should be of interest of to the community working in COF and other comparable materials for optoelectronic applications. The work is convincing except in few places which I detail below. The work is very interesting, but I am not sure that "it will influence thinking in the field". But, there only very few reports using ultrafast spectroscopy to interrogate COFs, MOFs and HOFs. The ms is reasonably well written (except that the Figures of S.I are cited according to the flow of information). The ms merits its publication in Nature Communication after considering the following points:

→ We appreciate the positive evaluation of our work by the reviewer #1.

1- P5, bottom: in the sentence "The electronic interaction between PDI and free-base porphyrin units is manifested as spectral red shift and broadening of the absorption spectrum of COFs compared with the absorption spectrum of freebase porphyrin, as shown in Figure 1b.", I have few comments:

a – the shift of the band at 420 nm could be the result of scatter from the sample. The other bands at longer wavelengths of absorption, far from scatter, do NOT show any shift). In any case, if the shift is not from scattering, the authors should precise that they refer to the 420 nm band, and to all the bands.

→ It seems that the scale of the figure in the original manuscript was too small to properly demonstrate the spectral shift and broadening. To remedy this problem, we added a new figure (Figure S6) that shows a magnified view of the absorption spectra in the Supplementary Information (SI). In the magnified view around 420 nm shown in Figure S6a, the synthesized COFs exhibit broadening of the absorption band in the Soret region as well as its spectral red shift. These spectral features of COFs, including broadening and spectral red shift of the absorption band, were also observed in a broad Q-band region from 450 nm to 800 nm, as shown in Figure S6b. Therefore, we concluded that the spectral shift in the Soret region is a real feature, not likely an artifact to originate from the unwanted scattering from the sample.

Following the reviewer's suggestion, we specified the absorption maxima of COFs with quantitative values in comparison with those of their precursor molecules. For this purpose, we revised the paragraph of "Femtosecond transient absorption spectroscopy" section in Results, as follows.

"... The intense absorption peak at 424 nm wavelength is mainly attributed to the B state (or Soret band) of free-base porphyrin unit, which has an absorption peak at 419 nm. The broad absorption from 470 nm to 700 nm with the peaks at 484 nm, 518 nm, 560 nm, 592 nm, and 652 nm can be assigned to the absorption arising from the electronic interaction between the $S_0 \rightarrow S_1$ transition of PDI (peaked at 481 nm and 516 nm) and the Q_y and Q_x transitions of free-base porphyrin (peaked at 513, 548, 590, and 646

nm). The electronic interaction between PDI and free-base porphyrin units is manifested as spectral red shift and broadening of the absorption spectrum of COFs compared with the absorption spectrum of free-base porphyrin, as shown in Figure 1b and Figure S6."

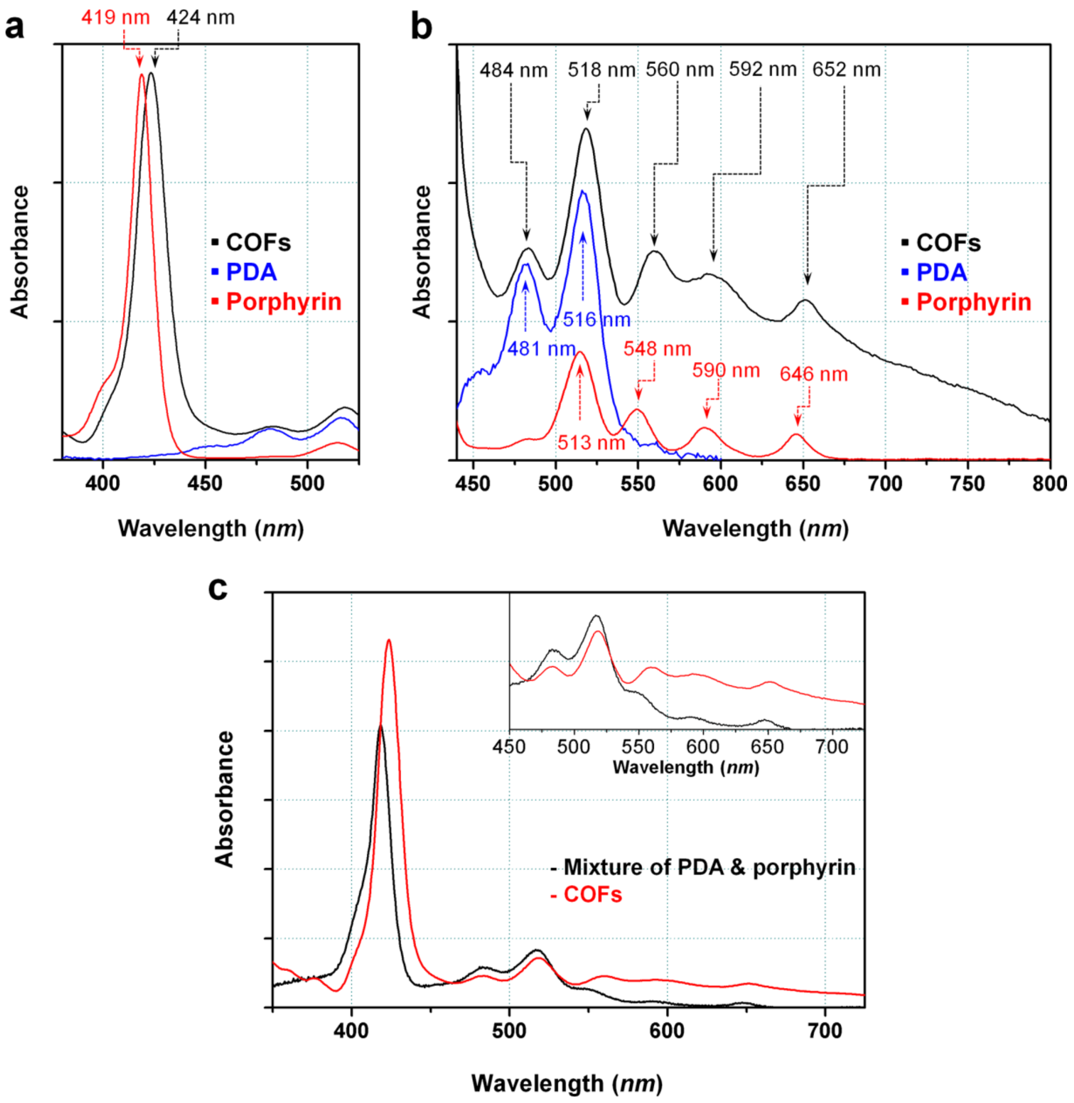

Figure S6. Steady-state absorption spectra of synthesized COFs compared with their precursors. Optical absorption spectrum of COFs dispersed in DMF (black), together with the normalized absorption spectra of their precursor molecules, free-base porphyrin (red) and perylene-3,4,9,10-tetracarboxylic dianhydride called as PDA (blue). (a, b) Absorption spectra of COFs and their precursors in the spectral range (a) from 380 nm to 525 nm, and (b) from 440 nm to 800 nm. The conjugation of PDA and free-base porphyrin moieties through the covalent bonds induces the spectral red shift of absorption maxima as well as broadening of the absorption band up to the near-infrared region. (c) Comparison of absorption spectrum of COFs (red) with that of a physical mixture of their precursor molecules (black). While the

physical mixture has low absorption at 575 – 660 nm, the synthesized COFs have strong absorption in a broad spectral range from 575 nm beyond 700 nm.

b- It seems that the shape of the COF band in the inset of Figure 1 (almost without intensity at 440-450 nm !) is different form the one shown in the main figure (with the absorption of porphyrin.

→ We compared the absorption spectra in the two figures in the Origin software and confirmed that they are identical with each other. I think they might look different because of the difference in the y-axis scale of the two plots. The peak at 424 nm has much higher intensity than other absorption features that we cropped the 424 nm peak in the main figure to show other smaller features better. For clarity, we modified Figure S6 to display the absorption spectra around 420 nm in panel (a) and the absorption spectra around the Q-band region from 440 nm to 800 nm in panel (b).

2- The excitation at 523 nm of the COF pumps both porphyrin and PDI moieties. The authors should consider this possibility and its implication in the observed dynamics.

→ As the referee pointed out, the excitation pulse of 523 nm can be resonant with both the Q_y band of free base porphyrin moiety and the $S_0 \rightarrow S_1$ transition of PDI moiety. Due to the spectral overlap of optical transitions, we cannot completely rule out the possibility that the relaxation of charge carriers generated from the direct excitation of porphyrin unit occurs. Nevertheless, in the separate fs-TA experiment of free-base porphyrin, the relaxation of charge carriers in porphyrin does not induce any significant decay of the TA signal, for example the quenching of stimulated emission at 490 − 625 nm. In addition, according to a previous spectroscopic study of perylene bisimide analogous to the PDI moiety (Kim *et al*, *J. Phys. Chem. A* **2015**, 119, 6275), the relaxation of excited carriers localized in the PDI moiety does not accompany any significant change of TA spectra in the time window up to 2 ps. Considering the results from the fs-TA experiment on free-base porphyrin and the previous spectroscopic study of perylene bisimide, the A → B transition of COFs with the time constant of 124 fs should be assigned to charge carrier transfer between the building units of COFs, rather than relaxation of charge carriers at local chromophores. According to the theoretical linear absorption spectrum of COFs simulated with TD-DFT (see Figure S17 in the revised SI), the optical transition probability of HOMO-3 → LUMO (peaked at 514 nm), which are localized in the PDI moiety, is twice larger than that of HOMO-1 → LUMO+1' (peaked at 513 nm), which are localized in the free-base porphyrin moiety. Therefore, the photoexcitation at 523 nm is likely to dominantly excite the HOMO-3 → LUMO transition in the PDI unit rather than the HOMO-1 → LUMO+1' transition in the porphyrin unit. As a result, the excited-state dynamics observed in the TA spectra of COFs should be mainly associated with the charge carrier transfer from PDI to porphyrin.

To address the reviewer's comment, we added the following paragraph at the end of "Femtosecond transient absorption spectroscopy" section in Results.

"... Meanwhile, the excitation at 523 nm can be resonant with both the $S_0 \rightarrow S_1$ transition of PDI (peaked at 516 nm) and the Q_y transition of free-base porphyrin (peaked at 513 nm). While the relaxation of excited charge carriers generated from direct excitation of free-base porphyrin unit can occur, it cannot account for the large change of TA spectra induced by the A → B transition shown in Figure 2b and 2d. In addition, the relaxation of photo-generated carriers localized in the PDI unit can occur due to the existence of defect sites that trap charge carriers. However, according to a previous TA study of perylene bisimide that is analogous to the PDI moiety (Kim *et al*, *J. Phys. Chem. A* **2015**, 119, 6275), the relaxation of excited population in the S_1 state does not accompany any significant change of TA spectra in the time window up to 2 ps. By considering our fs-TA experiment on free-base porphyrin and the previous spectroscopic study of perylene bisimide, we can confirm that the spectral change accompanying the A → B transition is associated with charge transfer between the building units of COFs, rather than relaxation of charge carriers at local chromophores."

We also added the following paragraph to the "Coherent wavepacket features in spectroscopic data" section in Results.

"…In addition, the excitation pulse at 523 nm can excite the Q_y transition of free-base porphyrin moiety. As shown in Figure S12, the FTPS from the separate fs-TA measurement of free-base porphyrin shows several major peaks, of which vibrational frequencies of 85 cm^{-1} and 321 cm^{-1} are distinctly different from the frequencies of 354, 405, 667, and 866 cm^{-1} observed in the neat DMF. Based on the previously reported resonance Raman and theoretical studies (Minaev *et al*, *Spectrochim. Acta A* **2006**, 65, 308 & Saini, *Spectrochim. Acta A* **2006**, 64, 981), the two peaks at 85 cm^{-1} and 321 cm^{-1} can be assigned to the torsional motion of side phenyl rings and the in-plane stretching of porphyrin, respectively. From the comparison of FTPS of COFs, neat solvent, and free-base porphyrin in the low-frequency region of < 300 cm^{-1}, we found that the COFs exhibit two prominent peaks at 76 cm^{-1} and 285 cm^{-1}, which are absent in the FTPS of neat DMF and free-base porphyrin monomer."

To note the implication of excitation of both PDI and free-base porphyrin moieties from a theoretical perspective, we added the following paragraph in Discussion.

"... In order to examine the excitation condition of fs-TA experiment, the theoretical linear absorption spectrum of COFs was calculated from the KS-DFT levels in conjunction with the ground-state MD simulation. As shown in Figure S17, the excitation at 523 nm can be resonant with both the HOMO-3 → LUMO transition localized in the PDI moiety (peaked at 514 nm) and the HOMO-1 → LUMO+1'

transition localized in the porphyrin moiety (peaked at 513 nm). As the transition probability of HOMO-3 → LUMO is twice larger than that of HOMO-1 → LUMO+1', the photoexcitation at 523 nm is likely to dominantly excite the optical transition localized in the PDI moiety. According to the NA-MD simulation on the charge carrier dynamics of COFs, the population decay of initially excited S_b state, which corresponds to the transition of HOMO-3 → LUMO, leads to concurrent growth of the populations of S_d and S_j states with the time constant of ~180 fs, matching the time scale of the ultrafast quenching of stimulated emission observed in the fs-TA data. ..."

3- The authors cite figure S14 (p. 6) before all the figures S1-S13. I see also that several figures in the SI are not cited/used in the ms.

→ We appreciate the reviewer's careful reading. To improve the readability of main text and SI of the manuscript, we rearranged the order of figures and tables in the SI following the order of appearance in the main text.

4- The negative signal from 500 to 560 nm, should contain a contribution from the excitation beam (523 nm) (p. 6).

→ As the referee pointed out, the TA signal in the probe region from 500 nm to 560 nm may contain the scattering from the excitation pulse due to the spectral overlap between the pump pulse and the broadband probe pulse. Since the magic-angle geometry (54.7º) was employed for the polarizations of pump and probe beams in the fs-TA system, significant amount of scattering of the pump pulse can be reduced by a Glan-Thompson polarizer located right before the detector. In addition, we cautiously positioned the sample cell in order to reduce any unwanted scattering off the surfaces of the cell. Nevertheless, the TA signal in the probe region from 500 nm to 560 nm may contain the scattering of the excitation pulse to some extent. We revised the paragraph of "Femtosecond transient absorption spectroscopy" section in Methods, as follows.

"... To avoid unwanted scattering of the excitation beam mixed into the TA signal, we cautiously positioned the sample cell in order to reduce any unwanted scattering off the surfaces of the cell. ..."

5- For the assignment of the dynamics (P. 6), using a stepwise process, the authors were too fast. Furthermore, if "A" is the initially excited species, we cannot label it as an intermediate. The intermediate is B.

→ Following the reviewer's suggestion, we assigned "A" state to an initially excited species and also changed the terminology of "intermediate" to "state". Considering those changes, we revised a paragraph of "Femtosecond transient absorption spectroscopy" section in Results, as follows.

"... where A is an initially excited state, and B and C are the excited states involved in the dynamics of excited charge carriers. The spectral features of each state are described by species-associated difference spectra (SADS) obtained from the global kinetic analysis, as shown in Figure 2b. ..."

6- The error in the time constant of the first step is too optimistic, taking into account the IRF of the full setup (70 fs).

→ We note that the errors of time constants reported in the manuscript are not the experimental uncertainties but the values of fitting error determined from the global kinetic analysis. To examine the effect of IRF width on the kinetics determined by the fitting, we performed a theoretical modelling of chemical kinetics convoluted with IRFs of various widths. As can be seen in Figure R1 below, the temporal broadening of IRF highly affects the maximum population of initial species generated by the photoexcitation. Since the dynamics with the time constant of 124 fs is well beyond the temporal width of experimentally determined IRF (68 fs), we argue that the initial event observed by the fs-TA measurement is not limited by the IRF of our TA measurement and thus the kinetics determined from our fitting is free from any temporal distortion.

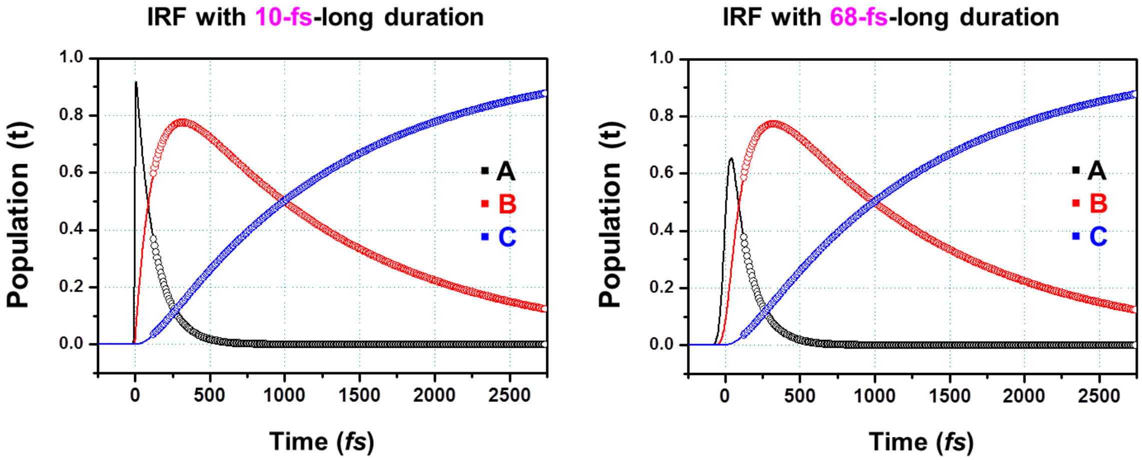

Figure R1. Chemical kinetics convoluted with the various IRF conditions. [Left] The modelled chemical kinetics is convoluted with the 10-fs-long duration of IRF with the Gaussian function. [Right] The modelled kinetics is based on 68-fs-long duration of IRF experimentally determined in this study.

7- Show in SI a comparison of the spectra to believe the following argument; (p. 7): As can be seen in Figure 2b, the 1st SADS shows strong GSB and SE bands in the spectral region from 490 to 625 nm and these spectral features are in good agreement with the steady-state absorption/emission spectra and broadband TA spectrum of PDI monomers reported previously26-28.

→ Following the reviewer's suggestion, we revised the following figure in SI (Figure S8) to show the steady-state absorption and emission spectra of PDI monomers together.

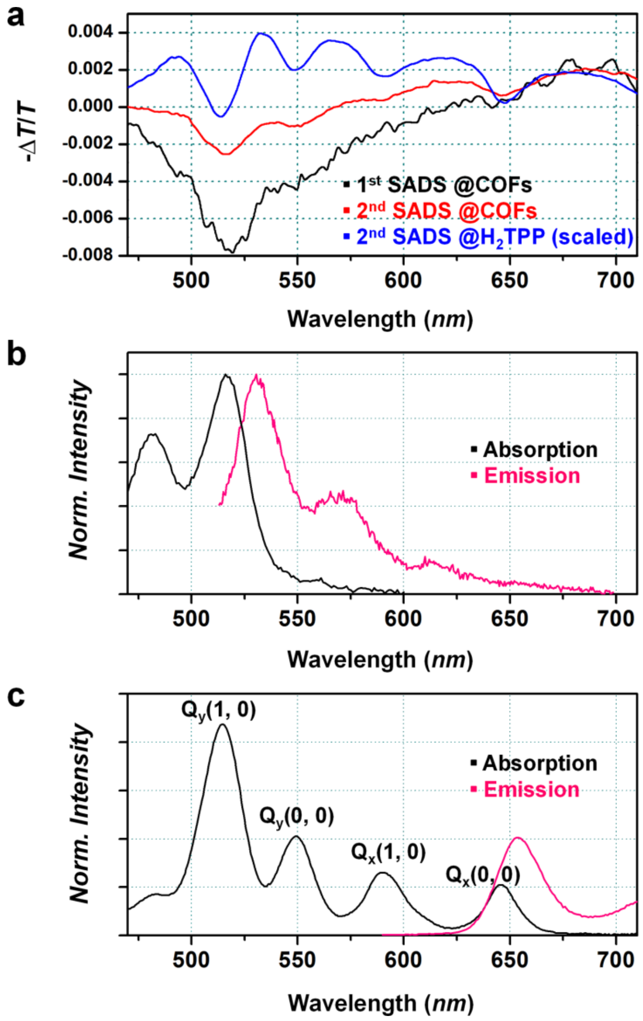

Figure S8. Species-associated difference spectra (SADS) of COFs and free-base porphyrin and steady-state absorption and emission spectra of precursor molecules. (a) 1st SADS and 2nd SADS of COFs are compared with 2nd SADS of free-base porphyrin (H_2TPP). (b) Linear absorption spectrum of PDI monomer (black line), together with the fluorescence emission spectrum (magenta line) measured with the diluted solution and the excitation at 483 nm. (c) Absorption (black line) and emission (magenta line) spectra of free-base porphyrin. The fluorescence emission spectrum was measured with the

excitation at 400 nm. The front and back numbers in parenthesis are the vibrational quantum numbers of the dominant vibrational mode in the upper and lower electronic states, respectively.

We also revised the paragraph of "Femtosecond transient absorption spectroscopy" section in Results, as follows.

"... As can be seen in Figure 2b, the 1st SADS shows strong GSB and SE bands in the spectral region from 490 to 625 nm and these spectral features are in good agreement with the steady-state absorption/emission spectra (Figure S8) and broadband TA spectrum of PDI monomers reported previously[26-28]. ..."

8- As I said, the excitation at 523 nm will bring both unit to their excited states, and therefore, the authors should reconsider the following statement in p. 7: "Thus, the intermediate A must be associated with PDI units of 2D COFs." Again "A" is not an intermediate.

→ The point of this comment is in line with that of comment #2. This argument in the section of Results was based on the comparison of the 1st SADS of COFs and the steady-state absorption/emission spectra of PDI monomer, as follows;

"... As can be seen in Figure 2b, the 1st SADS shows strong GSB and SE bands in the spectral region from 490 to 625 nm and these spectral features are in good agreement with the steady-state absorption/emission spectra (Figure S8) and broadband TA spectrum of PDI monomers reported previously[26-28]. ... "

Since the 1st SADS involves the prominent stimulated emission (SE) feature arising from the PDI moiety relative to the excited state absorption or SE feature from the porphyrin, we expected that the initially excited state (A) is likely to be associated with PDI units of 2D COFs. Nevertheless, we toned down this argument in order to consider the implication for the excitation of PDI and free-base porphyrin moieties by the excitation at 523 nm. The contents about the implication of co-photoexcitation were added in Results and Discussion (See Response to comment #2).

"... Thus, the A species is likely to be associated with PDI units of 2D COFs. ..."

9- P. 7: The authors state that "The SE is quenched further with the transition from B to C". I really do not see a significant decrease in the SE intensity.

→ To address the reviewer's concern, we added a new figure (Figure S9) which shows the difference between the 2nd SADS and the 3rd SADS. As the referee pointed out, the spectral shape of the 2nd SADS is similar to that of the 3rd SADS. However, the transition from the 2nd SADS to the 3rd SADS with the time constant of 1.25 ps was obviously observed in the time-dependent amplitude change of TA spectra

determined from the global kinetic analysis based on the SVD. As shown in Figure 2b, this transition involves the spectral change in the probe window covering from 490 to 625 nm that corresponds to the stimulated emission signal. Thus, we stated that the SE signal is indeed quenched with the 2nd SADS → 3rd SADS transition.

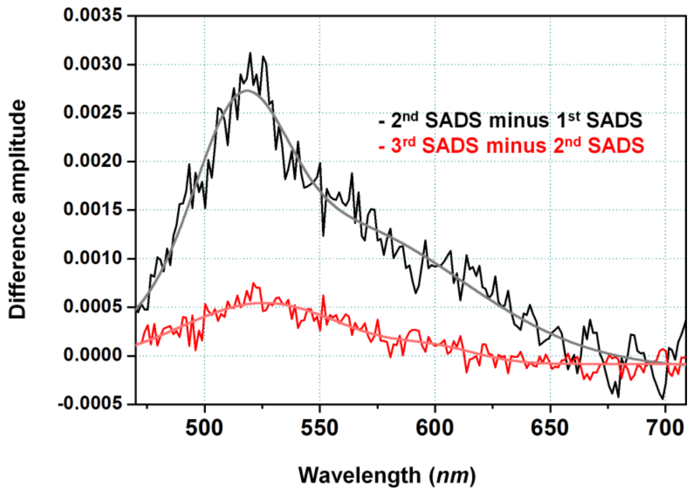

Figure S9. Double difference spectra between temporally adjacent species-associated difference spectra (SADS) of COFs. The difference of 2nd SADS and 1st SADS was scaled by a factor of 0.5 for clear comparison with that of 3rd SADS and 2nd SADS.

We also revised the paragraph of "Femtosecond transient absorption spectroscopy" section in Results, as follows.
"... The SE at 490 − 625 nm is quenched further with the transition from B to C as can be seen in the spectral and temporal changes from the 2nd SADS to 3rd SADS shown in Figure 2b and Figure S9. ..."

10- P7: indicate the longest time delay of the setup.

→ Following the reviewer's suggestion, we provided the information about the time delay of TA data in the "Femtosecond transient absorption spectroscopy" section in Methods, as follows.

"... The broadband TA data were measured at various time delays in the time range from -440 fs to 2728 fs with a time step of 8 fs. ..."

11- Again Figure S6b is cited in the text before citing Figs. S1-S5.

→ We appreciate reviewer's careful reading. To improve the readability of the manuscript, we rearranged the order of figures and tables in the SI to match the order of appearance in the main text.

12- A figure for comparison should be added in the SI to support the following: "When comparing the SADS of free-base porphyrin with its steady-state emission spectrum (Figure S6b), we can infer that the change of SADS at 610 − 710 nm is likely to be associated with the growth of SE contribution arising from the Qx(0,0) state of free-base porphyrin, which can be reached by Qy-to-Qx internal conversion and vibrational relaxation, as reported previously23,29,30."

→ Following the reviewer's suggestion, we revised Figure S8 shown below by including the steady-state absorption and emission spectra of free-base porphyrin monomers.

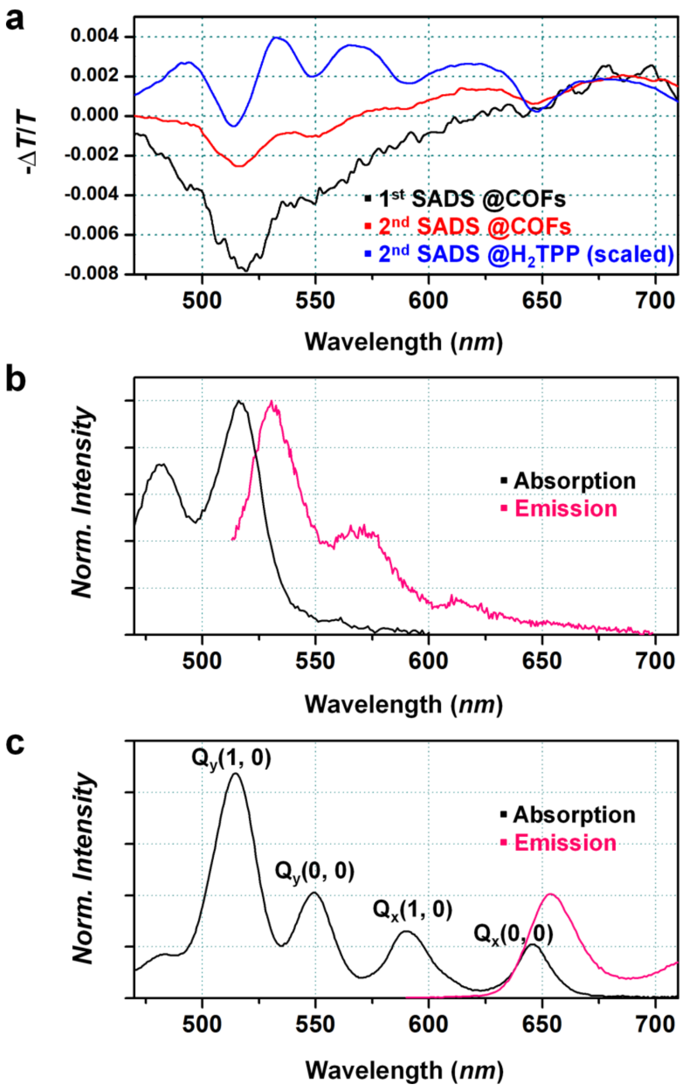

Figure S8. Species-associated difference spectra (SADS) of COFs and free-base porphyrin and steady-state absorption and emission spectra of precursor molecules. (a) 1st SADS and 2nd SADS of COFs are compared with 2nd SADS of free-base porphyrin (H_2TPP). (b) Linear absorption spectrum of PDI monomer (black line), together with the fluorescence emission spectrum (magenta line) measured

with the diluted solution and the excitation at 483 nm. (c) Absorption (black line) and emission (magenta line) spectra of free-base porphyrin. The fluorescence emission spectrum was measured with the excitation at 400 nm. The front and back numbers in parenthesis are the vibrational quantum numbers of the dominant vibrational mode in the upper and lower electronic states, respectively.

We also revised the paragraph of "Femtosecond transient absorption spectroscopy" section in Results, as follows.

"... When comparing the SADS of free-base porphyrin with its steady-state emission spectrum (Figure S8), we can infer that the change of SADS at 610 − 710 nm is likely to be associated with the growth of SE contribution arising from the $Q_x(0,0)$ state of free-base porphyrin, which can be reached by Q_y-to-Q_x internal conversion and vibrational relaxation, as reported previously[23,29,30]. ..."

13- The following sentence should be considered taking into account that the 523 nm excitation pulse, also excites the porphyrin unit. "Based on the results obtained from the TA measurements on COFs and free-base porphyrin, in the TA spectra of COFs, the quenching of SE at 490 – 625 nm arising from PDI units concurs with the growth of SE at 645 nm arising from free-base porphyrin units. Thus, the A → B transition of COFs with the time constant of 124 fs can be attributed to the ultrafast transfer of excited charge carriers from PDI to free-base porphyrin."

→ The point of this comment is in line with that of comment #2. To address the reviewer's comment, we added the following paragraph to the end of "Femtosecond transient absorption spectroscopy" section in Results.

"... Meanwhile, the excitation at 523 nm can be resonant with both the $S_0 \rightarrow S_1$ transition of PDI (peaked at 516 nm) and the Q_y transition of free-base porphyrin (peaked at 513 nm). While the relaxation of excited charge carriers generated from direct excitation of free-base porphyrin unit can occur, it cannot account for the large change of TA spectra induced by the A → B transition shown in Figure 2b and 2d. In addition, the relaxation of photo-generated carriers localized in the PDI unit can occur due to the existence of defect sites that trap charge carriers. However, according to a previous TA study of perylene bisimide that is analogous to the PDI moiety (Kim *et al*, *J. Phys. Chem. A* **2015**, 119, 6275), the relaxation of excited population in the S_1 state does not accompany any significant change of TA spectra in the time window up to 2 ps. By considering our fs-TA experiment on free-base porphyrin and the previous spectroscopic study of perylene bisimide, we can confirm that the spectral change accompanying

the A → B transition is associated with charge transfer between the building units of COFs, rather than relaxation of charge carriers at local chromophores."

14- As the authors have made the experiment in DMF, it will be interesting to examine the solvent effect in the theoretical results.

→ We absolutely agree with the reviewer's comment. The solvation dynamics coupled with the photoinduced charge transfer has been a central theme in the field of physical chemistry over many years. Even if the theoretical study of solvent effect is beyond the scope of this paper, we are highly motivated to do that in the future.

My major point is: The reached/excited species of this COF using 523 nm. At this wavelength (figure 1) both units of the COF absorb and then excited. So, the authors should reconsider their analysis and assignment of the observed times. The COF dynamics should be compared to both of porphyrin (alone as it is done) and of PDI (which is lacking) in DMF solutions.

→ The main concern of the reviewer is that the excitation pulse can be resonant with the electronic transitions of both PDI and free-base porphyrin moieties. This issue is also related with the reviewer comments #2, #8, and #13. The reviewer suggests that, to resolve this issue, the fs-TA data should be compared with those of free-base porphyrin and PDI. Actually, even before implementing our spectroscopic experiments, we already recognized the necessity of the fs-TA data for the constituents of synthesized COFs. First, we searched literature in order to get the information on the spectroscopic evidences of the constituent molecules of COFs. As both free-base porphyrin and PDI are well known as light-harvesting chromophores, they had been already studied by various spectroscopic methods. Soon we found that the excited state dynamics of free-base porphyrin was systematically studied by Zewail and co-workers using fs-TA and fluorescence up-conversion spectroscopies (Zewail *et al*, *J. Phys. Chem. A* **2002**, 106, 9837). Since that study was implemented with a probe of a narrow bandwidth, we performed a fs-TA experiment with a broadband probe, as shown in the present manuscript. In contrast, the excited-state dynamics of perylene bisimide that is almost identical to the PDI moiety was already studied by Kim and co-workers using the fs-TA system with IRF of 40 fs equipped with the broadband detection system (Kim *et al*, *J. Phys. Chem. A* **2015**, 119, 6275). Based on the previous spectroscopic study on perylene bisimide, we confirmed that the relaxation of excited population in the S_1 state does not accompany any significant change of TA spectra in the time window up to 2 ps. Considering our own fs-TA experiment on free-base porphyrin and the previous spectroscopic study of perylene bisimide, we can confirm that the ultrafast dynamics of COFs accompanying the prominent change of TA spectra is solely attributed to the charge transfer between the building units of COFs, rather than relaxation of charge carriers at local

chromophores. In the originally submitted manuscript, we should have cited the appropriate reference on the TA study of perylene bisimide (Kim *et al*, *J. Phys. Chem. A* **2015**, 119, 6275) and discussed the implication of the excitation of both PDI and free-base porphyrin moieties. Following the reviewer's comment, we revised the original contents and also added the discussions for the implication of the excitation of both PDI and free-base porphyrin moieties. The details about the revisions are described in the responses to the reviewer comments #2, #8, and #13.

<u>Responses to the comments from the reviewer #2</u>

In this manuscript, all of the calculations and hypotheses are based on the formation of 2D COFs. However, the authors did not provide enough evidence to prove the formation of 2D COF. Since the spectroscopy data are sensitive to the impurities and the packing mode of the resulting materials, these data are meaningless if they cannot confirm the purity of the resultant COF. Therefore, I do not recommend the publication of this manuscript.

1. The gas sorption data and pore size distribution analysis are missing. It's a standard procedure to characterize the structures of COFs. In addition, it's suggested to provide theoretical BET surface area and compared with the experimental data.

→ Following the reviewer's suggestion, we thoroughly characterized the porosity of synthesized COFs using gas sorption isotherms and added a new figure (Figure S5) containing nitrogen-adsorption isotherms and pore size distribution of COFs to the SI. The measured nitrogen-adsorption isotherms exhibit reversible uptake for gas molecules with a type IV isotherm indicative of mesopores. The Brunauer-Emmett-Teller (BET) surface area calculated from the gas sorption data is 309.4 m^2/g, which belongs to the region of mesoporous materials such as COFs or MOFs. We also checked the pore size distribution (PSD) of COFs calculated from the isotherm data. PSD exhibits a distribution peaked at ~3.86 nm as shown in the inset of Figure S5 and the pore size measured from the experiment is in agreement with the one typically predicted from the theoretically modelled structure of COFs.

We added the following paragraph to the "Characterization of covalent organic frameworks" section in supplementary information (SI), as indicated in the annotated version.

" ...

Porosity measurements. The porosity measurements were performed using the ASAP 2420 system (Micromeritics). The Brunauer-Emmett-Teller (BET) surface area of COFs was found to be 309.4 m^2/g. The nitrogen sorption isotherm measured at 77 K exhibits a type IV isotherm indicative of mesopores. The high adsorption at a high relative pressure (P/P_0 ~1) further corresponds to the presence of a mesoporous sample (Figure S5). Moreover, the curve of pore size distribution reveals a pore diameter of ~3.86 nm indicative of uniformly mesoporous COFs as shown in the inset of Figure S5. Overall, the results from the porosity measurements are in good agreement with those from the previously reported mesoporous COFs in literature[10,11].

..."

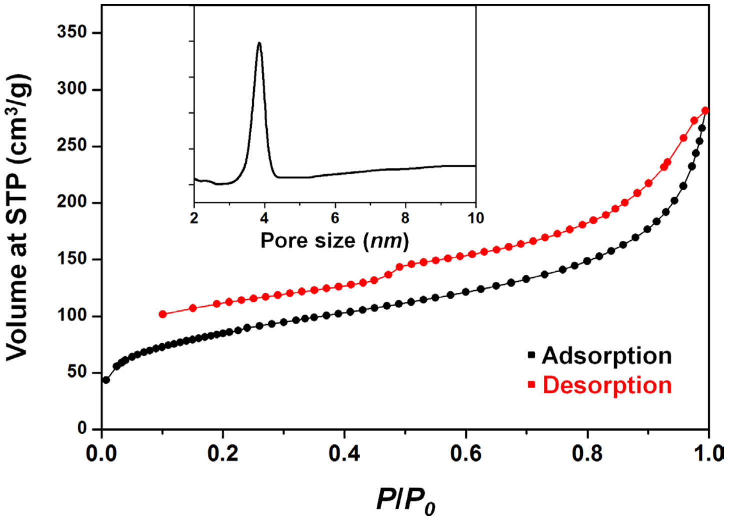

Figure S5. Nitrogen-adsorption isotherms and pore size distribution of COFs. Nitrogen adsorption-desorption isotherm of COFs was measured at 77 K. Inset shows pore size distribution of COFs.

2. Judging from the PXRD patterns shown in Fig. S2, it's more likely that crystals of oligomers or linear polymers rather than COFs are formed. The simulated PXRD patterns are completely different compared with the experimental data.

→ To extract structural information on the unit cell of COFs, we performed the Pawley refinement that is described in the "Simulation of powder X-ray diffraction patterns of covalent organic frameworks" section of SI as follows;

"... Based on this single-layer structure, the lattice parameters of eclipsed structure (space group $P1$, a = 29.84 ± 0.01 Å, b = 29.84 ± 0.01 Å, c = 3.65 ± 0.01 Å, $\alpha = \beta = \gamma$ = 90°) and staggered structure (space group $P1$, a = 29.84 ± 0.01 Å, b = 29.84 ± 0.01 Å, c = 7.30 ± 0.01 Å, $\alpha = \beta = \gamma$ = 90°) were determined by Pawley refinement of the PXRD pattern. ..."

If the crystals containing aggregated oligomers or linear polymers were dominant in the powder sample, as suggested by the reviewer, the Bragg positions of the PXRD pattern should not be reproduced by the Pawley refinement based on the theoretically modelled structures of COFs. However, according to the revised Figure S2, the refined X-ray profile shows excellent agreement with the experimental PXRD pattern. The good fidelity of the refined profile confirms the assignment of the observed reflections. In addition to the PXRD, the selected area electron diffraction (SAED) image, as shown in Figure S1b, clearly displays high-resolution Bragg spots that correspond to the interlayer distances between single layers of COFs, again confirming the crystallinity of COFs. The interlayer distance of 3.3 Å observed in

the SAED is consistent with the high-angle signal around 25º in the PXRD. The original manuscript has this argument described in the caption of Figure S1 as follows;

"... Selected area electron diffraction (SAED) image and profile plot of the area indicated by the magenta rectangle show a lattice spacing of 0.330 nm (d = 1/3.029 nm), which is close to the distance between 2D layers obtained by powder X-ray diffraction (PXRD) patterns of COFs. ..."

To avoid any ambiguity about the structural interpretation of PXRD pattern, we revised (1) the paragraph of "Simulation of powder X-ray diffraction patterns of covalent organic frameworks" section in SI as follows and (2) Figure S2 to show the observed X-ray diffraction profile together with the theoretical X-ray diffraction profile reconstructed from the Pawley refinement.

1) "... Based on the difference plot shown in Figure S2a, the calculated profile from the Pawley refinement based on the co-existence of eclipsed and staggered structures agrees well with the observed experimental PXRD pattern. The good fidelity of the refined profile confirms the assignment of the observed reflections. ..."

2) Revised Figure S2

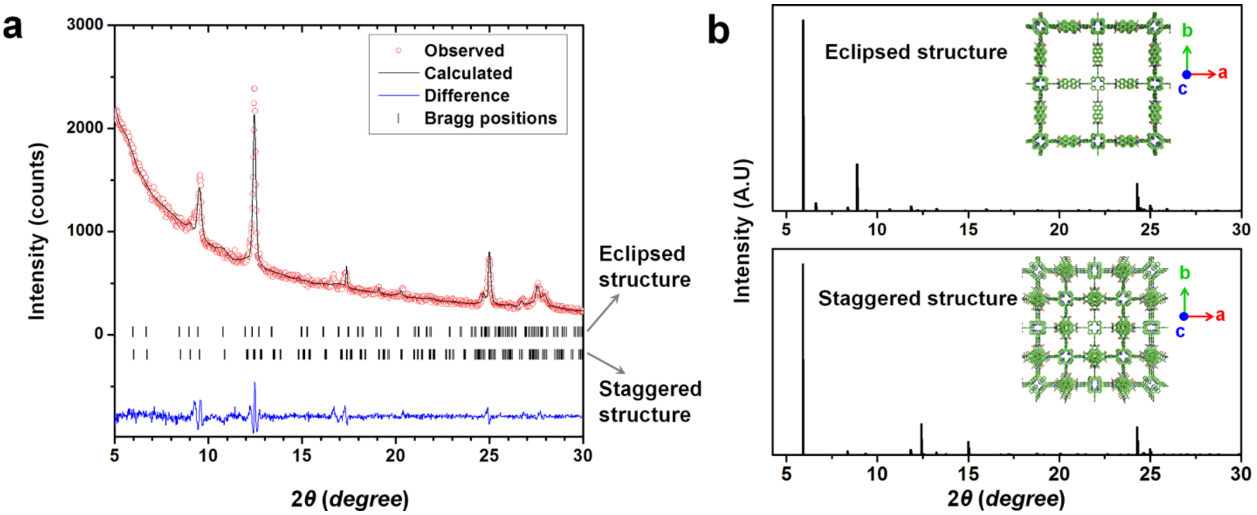

Figure S2. Powder X-ray diffraction (PXRD) patterns of COFs. (a) Experimental PXRD pattern of COFs. To confirm the assignment of the observed reflections, we performed the Pawley refinement of the experimental PXRD pattern (red circles) based on the co-existence of eclipsed and staggered structures shown in (b). The refined profile (black line) shows excellent agreement with the experimental PXRD pattern, leaving negligibly small discrepancy between the experimental and theoretically refined patterns

(blue line). The positions of Bragg reflections are marked by ticks for eclipsed (upper) and staggered (lower) structures. (b) Simulated PXRD patterns using the two different periodic orderings, eclipsed and staggered conformations, of COFs. In each panel, the molecular frameworks of stacked 2D sheets, constructed from the quantum mechanical optimized structure, are visualized along the direction of *c*-axis perpendicular to the paper plane. The relative intensities of Bragg peaks in the simulated PXRD patterns are different from those in the experimental PXRD pattern, which implies the preferred orientation of powder sample with anisotropic packing of crystallites.

3. The elemental analysis data of PDI-porphyrin COF should be provided.

→ Following the reviewer's suggestion, in the revised manuscript, we provide the contents about the elemental analysis in the "Characterization of covalent organic frameworks" section of SI.

At the last section in Supplementary note 2.

"...

Elemental analysis. The elemental analysis was performed using the FLASH EA-2000 Organic Elemental Analyzer (Thermo Scientific). The experimental data agree well with the expected analysis values; Anal. Calculated for $(C_{92}H_{42}N_8O_8)_n$: C 79.64; H 3.03; N 8.08 %. Found 78.65; H 3.08; N 8.24 %."

4. The spectroscopy data of corresponding physical mixtures are suggested to be provided.

→ Following the reviewer's suggestion, we compared the absorption spectrum of synthesized COFs with that of a physical mixture of their precursor molecules and added the corresponding data in Figure S6. According to the spectroscopic data, the synthesized COFs have the character of panchromatic absorption covering from near-UV to near-IR region up to 800 nm. In contrast, in the absorption spectrum of the physical mixture of PDA and free-base porphyrin in Figure S6c, the lowest absorption band is located at 646 nm, which is exactly the same wavelength as the peak position of the $Q_x(0, 0)$ band of free-base porphyrin. Therefore, we confirm that the conjugation of PDA and free-base porphyrin moieties through covalent bonds induces spectral red shift of absorption maxima as well as broadening of the absorption band up to the near-infrared region.

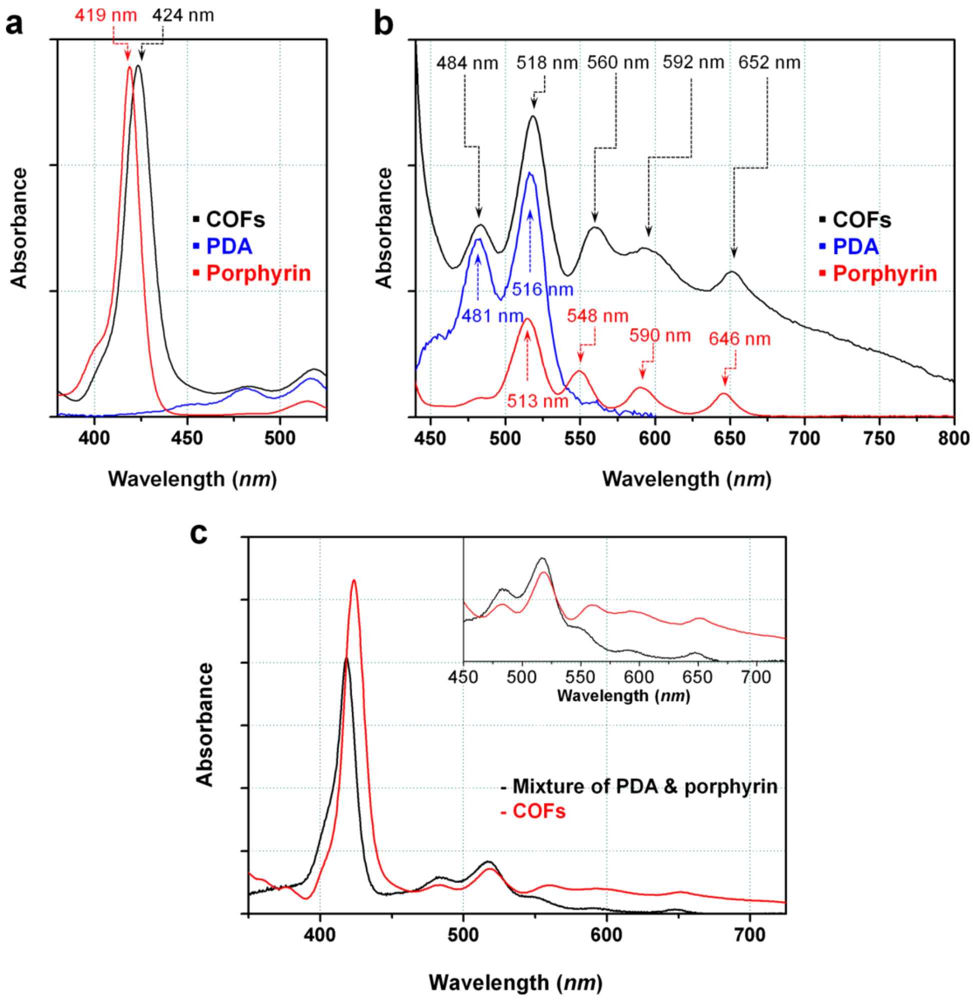

Figure S6. Steady-state absorption spectra of synthesized COFs compared with their precursors. Optical absorption spectrum of COFs dispersed in DMF (black), together with the normalized absorption spectra of their precursor molecules, free-base porphyrin (red) and perylene-3,4,9,10-tetracarboxylic dianhydride called as PDA (blue). (a, b) Absorption spectra of COFs and their precursors in the spectral range (a) from 380 nm to 525 nm, and (b) from 440 nm to 800 nm. The conjugation of PDA and free-base porphyrin moieties through the covalent bonds induces the spectral red shift of absorption maxima as well as broadening of the absorption band up to the near-infrared region. (c) Comparison of absorption spectrum of COFs (red) with that of a physical mixture of their precursor molecules (black). While the physical mixture has low absorption at 575 – 660 nm, the synthesized COFs have strong absorption in a broad spectral range from 575 nm beyond 700 nm.

Reviewers' comments:

Reviewer #1 (Remarks to the Author):

In this revised version, the authors have made strong arguments, providing precise explanation, and new data to support the discussion, in addition to adding new relevant references, and better description of the data and figures. They well addressed the points raised in my previous comments on the initial manuscript, and I am very satisfied with the provided explanation. I am also satisfied with the appropriateness and validity of the statistical analysis they provided, regarding the time constats of the involved photoevents.

So, I suggest to accept to publish this revised version in Nature Communications.

Abderrazzak Douhal

Reviewer #2 (Remarks to the Author):

The revised manuscript is improved. However, some issues are still needed to be addressed.
1. The sorption isotherm and pore size distribution of the COF indicates the formation of the mesoporous structure, which is in consistent of the modelled structure. But, obviously, there remains some impurities or defects judged from these data, they should carefully discuss the influence of these defects on their ultrafast photophysic properties.

2. The discussion and conclusion about the structure simulation is misleading. In Figure S2, the agreement of the refined X-ray profile with the PXRD pattern in Figure S2 cannot guarantee their conclusions, since the simulated PXRD patterns (Figure S2b) are completely different with the experimental data. Even in Figure S2a, the bragg positions of eclipsed structure and staggered structure obviously did not appears in the experimental data. The refined X-ray profile can only suggest the possible cell parameters and space groups. But they use P1 space group without any symmetry to construct the modelled structure, the refined data can always fit the experimental data as long as the cell parameters are reasonable. In addition, they should provide the PXRD data of the monomers.

Relatively, the formation of the mesopore with 3.9 nm diameter confirmed by sorption measurement is a more direct evidence. The direct observation of the ordered lattice attributing to the porous structure in TEM images will be a stronger evidence. Currently, the SAED image only reveals the interlayer distance is 3.3 Å but do not show any information on the ordered tetragonal structure.

Responses to the comments from the reviewer #1

In this revised version, the authors have made strong arguments, providing precise explanation, and new data to support the discussion, in addition to adding new relevant references, and better description of the data and figures. They well addressed the points raised in my previous comments on the initial manuscript, and I am very satisfied with the provided explanation. I am also satisfied with the appropriateness and validity of the statistical analysis they provided, regarding the time constats of the involved photoevents.

So, I suggest to accept to publish this revised version in Nature Communications.

→ We greatly appreciate the positive evaluation by the reviewer.

Responses to the comments from the reviewer #2

The revised manuscript is improved. However, some issues are still needed to be addressed.

1. The sorption isotherm and pore size distribution of the COF indicates the formation of the mesoporous structure, which is in consistent of the modelled structure. But, obviously, there remains some impurities or defects judged from these data, they should carefully discuss the influence of these defects on their ultrafast photophysic properties.

→ Following the reviewer's suggestion, we added the following discussion to the "Discussion" section in the main text.

" …

While the pore size of COFs determined from the nitrogen isotherm shown in Figure S5 is close to that of theoretically modelled structure shown in Figure S2 (see Supplementary Note 3 in the SI for details), we cannot completely rule out the presence of defects such as mislocation of building units in a 2D layer or less-ordered stacking between the 2D layers. Since the defects in the 2D layer may retard or block the excitons initially formed in the PDI moieties from being split into charge carriers (that is, electrons and holes) through charge transfer between PDI and porphyrin, the spectroscopically measured rates of charge transfer can be regarded as the lower limit for the defect-free material. Considering the prominent change of TA spectra associated with the ultrafast charge transfer between the building units in an individual 2D layer, the observed dynamics is highly unlikely to originate from the defects in a 2D layer. The analyses of TEM images and PXRD pattern show that the synthesized COFs adopts the staggered structure along the *c*-axis with slipping along *a*-axis and/or *b*-axis, as shown in Figure S2b. Such staggered inter-layer structure would result in inefficient charge migration between the stacked 2D layers. Moreover, considering that the TA measurement was conducted for the COF sample dispersed in solution phase, it is possible that the stacked layers in the solid phase can be separated into much thinner stacks or even single

layers. All these considerations lead us to conclude that the electrons and holes generated from the photoinduced charge transfer are likely to undergo charge recombination only within individual 2D layers. … ”

2. The discussion and conclusion about the structure simulation is misleading. In Figure S2, the agreement of the refined X-ray profile with the PXRD pattern in Figure S2 cannot guarantee their conclusions, since the simulated PXRD patterns (Figure S2b) are completely different with the experimental data. Even in Figure S2a, the bragg positions of eclipsed structure and staggered structure obviously did not appears in the experimental data. The refined X-ray profile can only suggest the possible cell parameters and space groups. But they use P1 space group without any symmetry to construct the modelled structure, the refined data can always fit the experimental data as long as the cell parameters are reasonable. In addition, they should provide the PXRD data of the monomers.

Relatively, the formation of the mesopore with 3.9 nm diameter confirmed by sorption measurement is a more direct evidence. The direct observation of the ordered lattice attributing to the porous structure in TEM images will be a stronger evidence. Currently, the SAED image only reveals the interlayer distance is 3.3 Å but do not show any information on the ordered tetragonal structure.

→ Regarding the Pawley refinement, we note that the molecular structure of repeat units, which was optimized by the DFT calculation, allows only the P1 space group. In other words, we did not choose the P1 space group intentionally or arbitrarily. While the exact crystal structure cannot be determined unambiguously by Pawley refinement, it should be noted that two-dimensional COFs generally tend to have weak ordering when they are stacked. Nevertheless, as the reviewer correctly pointed out, the mesopore of 3.9 nm diameter in the 2D layer and the inter-layer separation of 3.3 Å distance are evident from the nitrogen isotherm measurement and the SAED image, respectively.

To follow the reviewer's suggestion, we tried to measure high-resolution TEM (HR-TEM) images that can provide direct evidence for the rectangular structure of 2D layers with the help of two additional authors who have expertise in HR-TEM. The TEM measurement on COFs was challenging because the organic material was easily damaged by the electron beam of 300 keV energy, even with a low dose. Nevertheless, fortunately, we were able to capture HR-TEM images that show the regularity in the 2D layers of COFs, and thus we added those images as new figures in the SI (Figure S1d – S1i). It can be clearly seen that the spacing between periodic lines is about 1.0 nm and its FT image shows Bragg spots, which correspond to (220) planes. In addition, we obtained the Fourier-transformed (FT) image with Bragg spots for the lines with the spacing of 1.5 nm, which corresponds to (200), or (020), planes, although its HR-TEM image is too noisy to observe the 1.5-nm spacing in real space. The 1.5 nm spacing corresponds to half the distance between the nearest perylene moieties that are periodically aligned along

a-axis or *b*-axis in the 2D layers. The 1.0 nm spacing corresponds to half the distance between the farthest perylene moieties (along the diagonal) of the smallest rectangle formed by 2D layers stacked in the staggered conformation (that is, the diagonal length of the rectangle is 1.5 nm times the square root of 2). Thus, these TEM images strongly support the staggered structure along the *c*-axis with slipping along *a*-axis and/or *b*-axis.

Since the TEM measurement supports the staggered structure, we examined which structure between the eclipsed and staggered structures works better for the Pawley refinement, as shown in Figure S2. The result shows that the PXRD pattern is indeed more consistent with the mixed staggered structures with the A-stacking, B-stacking, and AB-stacking.

Thus, considering the analyses of TEM images and PXRD pattern, we can conclude that the synthesized COFs have a staggered conformation. As discussed in our response to the first comment, the absence of signature for the inter-layer charge migration in the femtosecond TA spectra is consistent with the staggered structure.

We added the following short discussion on the structure of COFs to "Femtosecond transient absorption spectroscopy" section in Results.

"… The porous structure of COFs was characterized by transmission electron microscopy (TEM), nitrogen isotherm, and powder X-ray diffraction (PXRD). The spacing between 2D layers is clearly visualized by a selected area electron diffraction (SAED) image, and the line spacing was determined to be 0.330 nm as shown in Figure S1. The pore size distribution from the nitrogen isotherm measurement shows a peak at 3.9 nm, which is similar to the distance between the farthest perylene moieties (along the diagonal) of the smallest rectangle unit in a 2D layer. HR-TEM images show 1.5 nm and 1.0 nm line spacings, which correspond to (200), or (020), and (220) planes, respectively. As discussed in the Supplementary Note 2, HR-TEM data support the staggered structure along the *c*-axis with slipping along *a*-axis and/or *b*-axis. Moreover, the PXRD pattern is also more consistent with the staggered structure as shown in Figure 1a. …"

A more detailed description regarding the structure determination including the new TEM results was added to "Supplementary Note 2" in the SI.

"… HR-TEM images shown in Figure S1d – S1i clearly show the regular pattern in the 2D layers. In particular, from the TEM image shown in Figure S1e, it can be learned that the spacing between periodic lines is about 1.0 nm and its FT image shown in Figure S1f has Bragg spots, which correspond to (220) planes. The 1.0 nm spacing corresponds to half the distance between the farthest perylene moieties (along the diagonal) of the smallest rectangle formed by 2D layers stacked in the staggered conformation (that is,

the diagonal length of the rectangle is 1.5 nm times the square root of 2). In addition, we obtained the Fourier-transformed (FT) image (Figure S1i) with Bragg spots for the lines with the spacing of 1.5 nm, which corresponds to (200), or (020), planes, although its HR-TEM image in Figure S1h is too noisy to observe the 1.5-nm spacing in real space. The 1.5 nm spacing corresponds to half the distance between the nearest perylene moieties that are periodically aligned along a-axis or b-axis in the 2D layers. Thus, these TEM images strongly support the staggered structure along the *c*-axis with slipping along *a*-axis and/or *b*-axis.

Also, the following description regarding the structure determination including the new TEM results was added to “Supplementary Note 3” in the SI.

“… Since the TEM measurement supports the staggered structure, we examined which structure between the eclipsed and staggered structures works better for the Pawley refinement as shown in Figure S2a – S2b. The result shows that the PXRD pattern is indeed more consistent with the mixed staggered structures with the A-stacking, B-stacking and AB-stacking. Thus, considering the analyses of TEM images and PXRD pattern, we can conclude that the synthesized COFs have a staggered conformation. As discussed in the main text, the absence of signature for the inter-layer charge migration in the femtosecond TA spectra is consistent with the staggered structure.”

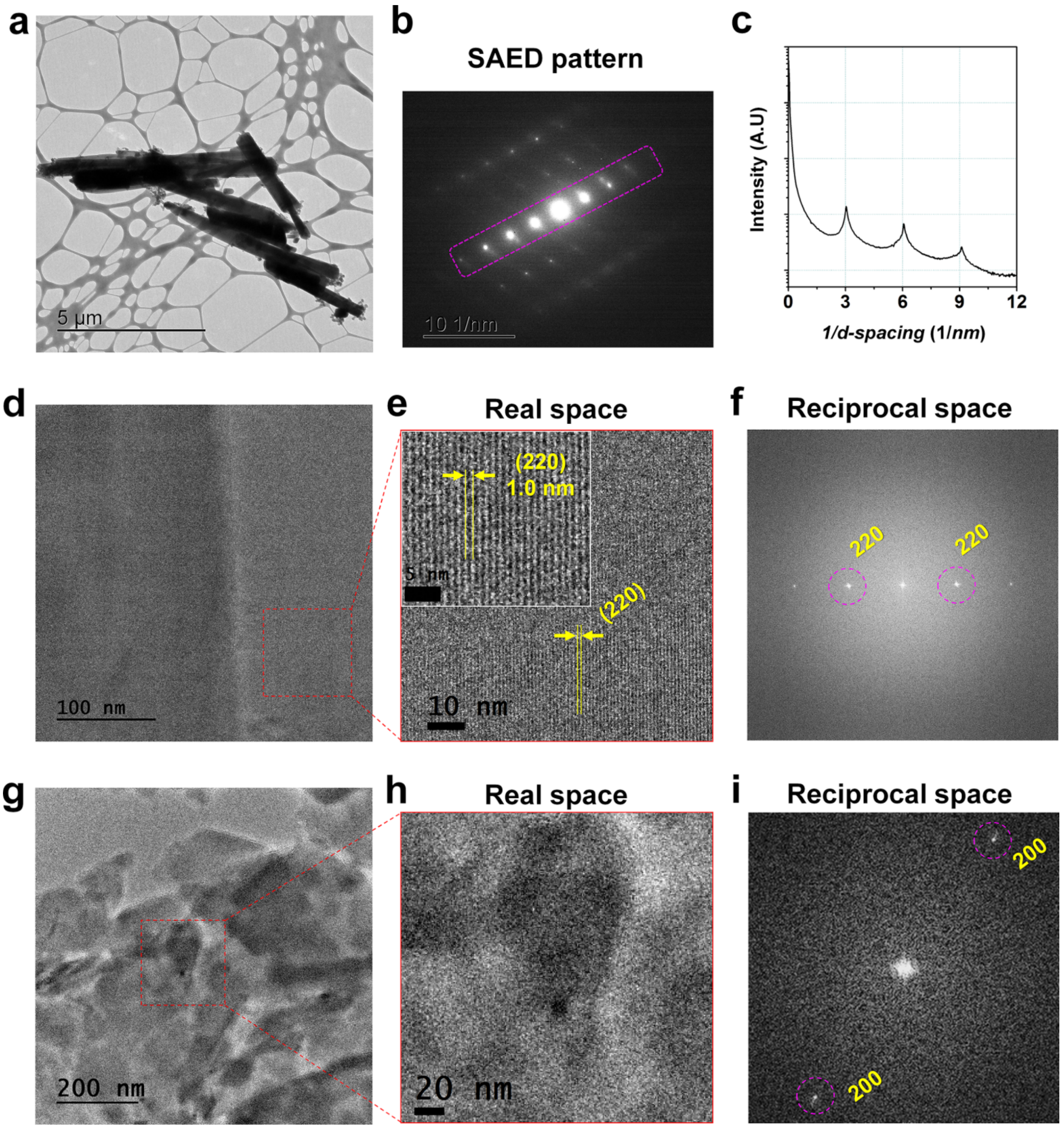

Figure S1. Transmission electron microscopy (TEM) measurement of COFs. (a) TEM image of COFs in powder form. (b) A selected area electron diffraction (SAED) image. (c) The profile plot of the area indicated by the magenta rectangle in (b). The lattice spacing (*d*-spacing) of 0.330 nm (= 1/3.029 nm) is close to the distance between 2D layers obtained by powder X-ray diffraction (PXRD) patterns of COFs. (d) A high-resolution TEM (HR-TEM) image of COFs for (220) planes. (e) A magnified view of (d). Periodic lines corresponding to the (220) planes are visible. The upper left inset shows a further

magnified view. The line spacing is about 1.0 nm. (f) Fourier-transform (FT) of the HR-TEM image in (e). The Bragg peaks for (220) planes with 1.06 nm spacing are seen. (g) A HR-TEM image of COFs for (200), or (020), planes. (h) A magnified view of (g). (i) FT of the HR-TEM image in (h). The Bragg peaks for (200), or (020), planes with 1.5 nm spacing are seen.

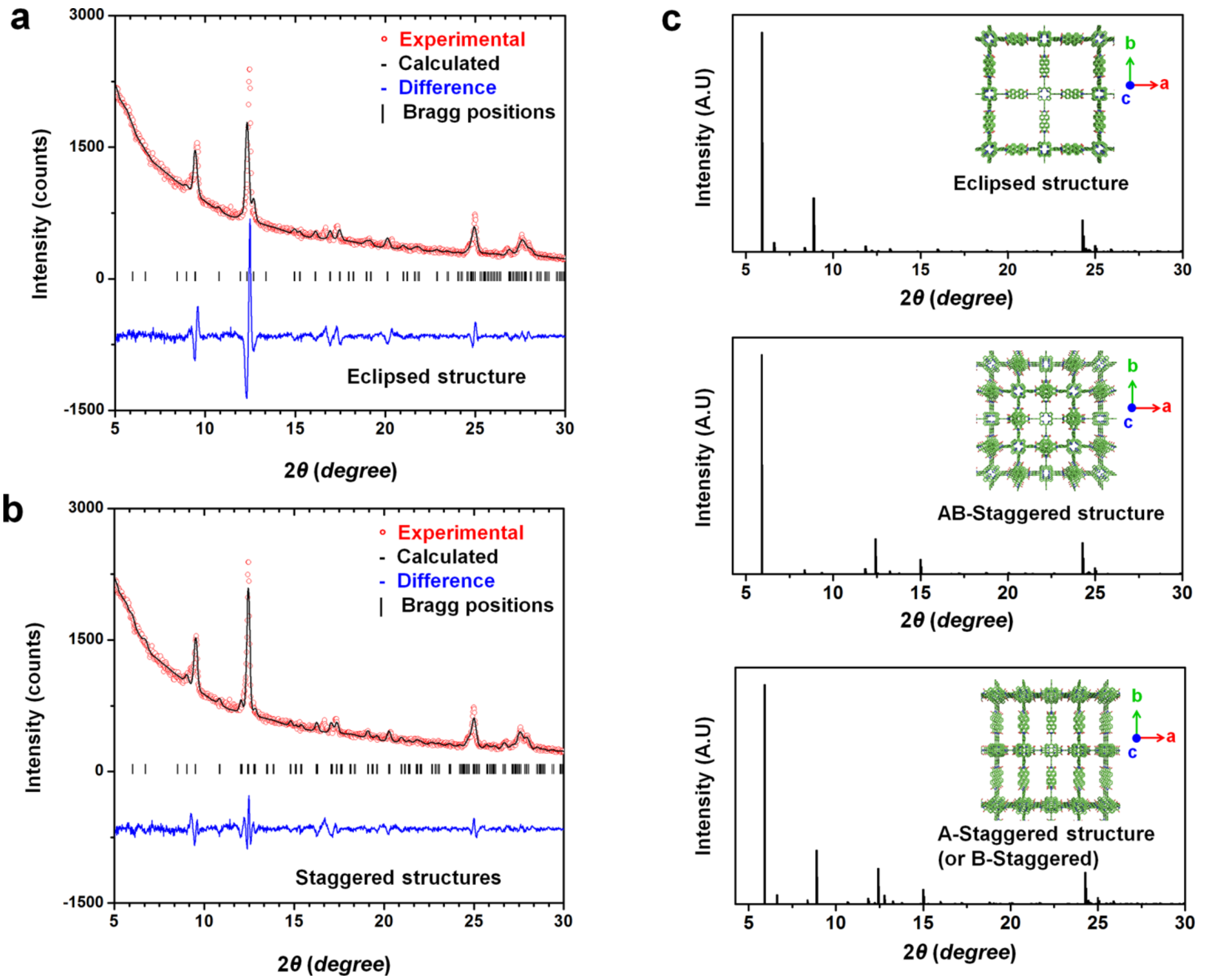

Figure S2. Powder X-ray diffraction (PXRD) patterns of COFs. (a, b) Experimental PXRD pattern of COFs. To confirm the assignment of the observed reflections, we performed the Pawley refinement of the experimental PXRD pattern (red circles) based on the (a) eclipsed and (b) mixed staggered structure, as shown in (c). The positions of Bragg reflections are marked by ticks for each of the eclipsed and staggered structures. The refined profile (black line) with the mixed staggered structure shows better agreement with the experimental PXRD pattern, leaving smaller discrepancy (blue line) between the experimental and theoretically refined patterns. This observation is consistent with the HR-TEM images shown in Figure S1. (c) Simulated PXRD patterns using the three different periodic orderings, i.e. eclipsed, staggered (AB stacking), and staggered (A or B stacking) conformations, of COFs. In each panel, the molecular frameworks of stacked 2D sheets, constructed from the quantum mechanical optimized structure, are visualized along the direction of *c*-axis perpendicular to the paper plane. The relative intensities of Bragg peaks in the simulated PXRD patterns are different from those in the experimental

PXRD pattern, which implies the preferred orientation of powder sample with anisotropic packing of crystallites.

REVIEWERS' COMMENTS:

Reviewer #2 (Remarks to the Author):

The revised manusript now can be accepted for publication.
